# SH3BP2 deficiency in skeletal muscle leads to neuromuscular junction abnormalities and decreased muscle strength

**DOI:** 10.3389/fnmol.2026.1985823

**Published:** 2026-10-05

**Authors:** Bhola Shankar Pradhan, Krzysztof M. Bernadzki, Yauhen Bandaruk, Anna Siudzińska, Teresa De Cicco, Małgorzata Sotomska, Marta Gawor, Margareta Jabłońska, Joanna Krzemień, Grzegorz Chodaczek, Said Hashemolhosseini, Tomasz J. Prószyński

**Affiliations:** 1 Łukasiewicz Research Network—PORT Polish Center for Technology Development, Wrocław, Poland; 2 Nencki Institute of Experimental Biology, Polish Academy of Sciences, Warsaw, Poland; 3 Institute of Biochemistry, Friedrich-Alexander University Erlangen-Nürnberg, Erlangen, Germany

**Keywords:** acetylcholine receptors, AChR clustering, dystrobrevin, neuromuscular junctions, SH3BP2, skeletal muscle

## Abstract

Assembly and stabilization of a high density of acetylcholine receptors (AChRs) at the neuromuscular junction postsynaptic domain is fundamental for proper synaptic transmission. Here, we report the identification of a scaffold protein, SH3BP2, that exhibits polyvalent and direct interactions with the dystrophin-glycoprotein complex (DGC) and AChR pentamers. Loss-of-function experiments in cultured myotubes revealed that SH3BP2 promotes formation of AChR assemblies in muscle cells. Similarly, muscle-specific deletion of SH3BP2 in mice impairs AChR clustering during embryonic development and causes multiple neuromuscular junction morphological and ultrastructural abnormalities in adult animals. Mutant mice also exhibit defects in synaptic transmission, muscle strength, and general fitness. Our study identifies a novel role for SH3BP2 in modulating the neuromuscular junction in skeletal muscles.

## Introduction

The proper organization of the neuromuscular junction (NMJ) is a complex process and requires maintaining the high density of the acetylcholine receptors (AChR) on the muscle fiber surface beneath the motor neuron termini (Li et al., 2018; Tintignac et al., 2015). Postsynaptic machinery assembly begins early in embryonic development, with muscle fibers spontaneously forming AChR clusters on their surface. As motor axons approach developing muscle fibers, they selectively innervate a subset of the prepatterned AChR clusters, whereas the non-innervated clusters are progressively dispersed (Lin et al., 2001; Yang et al., 2001). After proper junctions are formed, motor neurons regulate postsynaptic organization by secreting postsynaptic regulators, including acetylcholine (ACh), which activates AChR while promoting its internalization, and agrin, which stimulates postsynaptic development and counterbalances neurotransmission effects (Lin et al., 2001; Samuel et al., 2012; Sanes and Lichtman, 2001; Yang et al., 2001). At the postsynaptic site, agrin interacts with Lrp4-MuSK complexes, leading to MuSK-dependent phosphorylation of downstream components, including Dok-7 and the β subunit of the AChR pentamer (Friese et al., 2007; Inoue et al., 2009; Kim et al., 2008; Li et al., 2018; Okada et al., 2006). AChR phosphorylation provides an additional binding site for the major AChR clustering protein rapsyn (Borges et al., 2008; Huebsch and Maimone, 2003). Rapsyn also binds the α AChR subunit and aggregates receptors through oligomerization and phase separation (Lee et al., 2009; Li et al., 2016; Xing et al., 2021).

The dystroglycan glycoprotein complex (DGC) is a macromolecular transmembrane complex that stabilizes membrane and peripheral proteins (including postsynaptic components) by linking them to the extracellular matrix (ECM) and the cytoskeleton (Ervasti and Campbell, 1993). Mutations in several DGC proteins often cause severe muscular dystrophies characterized by muscle weakness and muscle mass loss, and are often fatal (Belhasan and Akaaboune, 2020; Grady et al., 1997b; Grady et al., 2000; Kim et al., 2019). However, the molecular functions of DGC components remain incompletely understood. α-dystrobrevin-1 (αDB1) is a cytoplasmic component of the DGC that plays an important role in AChR organization at the NMJ (Belhasan and Akaaboune, 2020; Grady et al., 1999; Grady et al., 2000). It has been established that the synaptic functions of αDB1 depend on phosphorylation of the C-terminally located tyrosine residues (Grady et al., 2003; Schmidt et al., 2011). However, the molecular mechanisms by which αDB1 and its phosphorylation regulate postsynaptic machinery remain unclear.

Here, we report that αDB1 phosphorylation at Y713 recruits a scaffold protein, SH3BP2. This protein has been implicated in signaling regulation in immune and bone cells (Chen et al., 2012; Deckert et al., 1998; Levaot et al., 2011b; Ueki et al., 2001), but its function in skeletal muscle has not been reported. We show that SH3BP2 is enriched in the muscle postsynaptic machinery and interacts with several DGC components. At the same time, SH3BP2 forms polyvalent interactions with AChR pentamers, promoting their aggregation. Muscle-specific SH3BP2 knockout leads to abnormal NMJ organization and function. Mutant mice exhibit impairments in synaptic transmission, muscle strength, and overall fitness.

## Materials and methods

### Ethics

Procedures using animals were conducted in compliance with the ethics committee guidelines for animal experimentation and regulations at the Łukasiewicz–PORT, the Nencki Institute of Experimental Biology, Poland (permissions 709/2015, 723/2015, 065/2020/P1, 066/2020/P1, 085/2021/DZ/NZP/P1, 058/2023/NZP), and the Friedrich-Alexander University Erlangen-Nürnberg, Germany (permissions I/39/EE006, TS-07/11).

### Animals

All the mice used in this study had a C57BL/6 genetic background (RRID: IMSR_JAX:000664). Myf5-Cre mice (RRID: IMSR_JAX:007893) were a kind gift from Markus Ruegg (University of Basel, Switzerland). Conditional SH3BP2 KO mouse strain (Sh3bp2tm1c(KOMP)Wtsi; RRID: MGI:6150898) with the loxP sites flanking exons 6 and 7 of the SH3BP2 gene was from the Canadian Mouse Mutant Repository at The Hospital For Sick Children (Toronto, Canada; Bradley et al., 2012) and was crossed with Myf5-Cre mice (RRID: IMSR_JAX:007893) to generate muscle-specific knockout of SH3BP2 (SH3BP2 mKO). This study used both male and female mice. Muscle strength and animal fitness experiments were performed on males. The animals were kept in the animal house on a 12-h light/dark cycle with free access to food and water. To reduce animal stress during the experiments, handling was conducted for 5 consecutive days prior to the procedures. No animals were excluded from the study.

### Antibodies and staining

All primary antibodies used in this study are shown in Supplementary Table 2. For immunofluorescence studies, the following secondary antibodies were used: goat anti-rabbit IgG (H + L) Alexa Fluor 488 (Thermo Fisher Scientific Cat# A-11034, RRID: AB_2576217), Alexa Fluor 546 (Thermo Fisher Scientific Cat# A-11035, RRID: AB_2534093) or Alexa Fluor 568 (Thermo Fisher Scientific Cat# A-11011, RRID: AB_143157); goat anti-mouse IgG (H + L) conjugated with Alexa Fluor 488 (Thermo Fisher Scientific Cat# A28175, RRID: AB_2536161) or Alexa Fluor 568 (Thermo Fisher Scientific Cat# A-11004, RRID: AB_2534072). The secondary antibodies for Western blotting were HRP-conjugated polyclonal anti-IgG antibodies (R and D Systems Cat# FAB7076S, RRID: AB_3652201 and Cell Signaling Technology Cat# 7074, RRID: AB_2099233). To visualize the AChR in the myofibers, myotubes, we used fluorescently labeled α-bungarotoxin (B35451 or B13422; Thermo Fisher Scientific, RRID: AB_2617152). Alexa Fluor 546 Phalloidin (A22283, Thermo Fisher Scientific) was used to visualize F-actin, and fasciculin II (F-225; Alomone Labs, labeled with the FluoReporter FITC kit from Thermo Fisher Scientific) was used to label AChE.

### Plasmids and siRNA

The EGFP–αDB1 (mouse) plasmid and the CMV-Rapsyn- mCherry plasmid were described previously (Alvarez-Suarez et al., 2021; Grady et al., 2003). The plasmids encoding the full-length α1, β1, δ, and ε nAChR subunits were kindly provided by Dr. Lin Mei (Case Western Reserve University, USA). SH3BP2 (mouse) and its domains (PH, PR, and SH2) were subcloned into a modified pCDNA3.1 backbone containing a FLAG, GFP, or Myc tag. SH3BP2 was also cloned into pBiFC-VC155 (Shyu et al., 2006), a gift from Chang-Deng Hu (RRID: Addgene_22011), and αDB1 was cloned into pBiFC-VN173 (Shyu et al., 2006), a gift from Chang-Deng Hu (RRID: Addgene_22010). The full-length syntrophin-1 (mouse), a cytoplasmic loop of AChRα (mouse; amino acid 317–428), a cytoplasmic loop of AChRγ (mouse; amino acid 330–476), and the cytoplasmic domain of β-dystroglycan (mouse; amino acid 773–893) were cloned into the EGFP-N2 plasmid. For protein purification from bacteria, we cloned the cytoplasmic domain of AChRα (mouse; amino acid 317–428) with or without mCherry into pGEX-4 T1 (Cytiva 289,545–49; Merck). GFP-SH3BP2 with or without the intrinsic disorder domain (amino acid 164–449) into pEBG-GST81, a gift from David Baltimore (RRID: Addgene_22227). Knockdown experiments were performed with the following siRNAs: siRNA1 (SI02739478, Qiagen) and siRNA2 (SI02692193, Qiagen), targeting SH3BP2, and a non-targeting control siRNA (12,935,300, Invitrogen).

### Protein complex purification and mass spectrometry analysis

For identification of SH3BP2-interacting proteins, HEK293 cells overexpressing TAP-GFP-SH3BP2 were lysed in lysis buffer (50 mM Tris–HCl [pH 8.0], 150 mM NaCl, 1% Igepal CA-630 [NP-40], 0.5% sodium dodecyl sulfate [SDS], 10% glycerol, 1 mM dithiothreitol [DTT], 1 mM NaF) supplemented with Mini Protease Inhibitors cocktail (11,873,580,001, Roche) and incubated with Dynabeads M-270 Epoxy (14311D, Invitrogen) coated with Rabbit IgG (I5006-10MG, SIGMA) overnight with rotation at 4 °C. The next day, the beads were washed four times with wash buffer (50 mM Tris–HCl [pH 8.0], 500 mM NaCl, 1% NP-40, 0.5% SDS, 1 mM DTT, 1 mM NaF, and Mini Protease Inhibitors Cocktail). The beads enriched with TAP-GFP-SH3BP2 were equilibrated with C2C12 cells lysis buffer (50 mM Tris–HCl [pH 8.0], 150 mM NaCl, 0.1% NP-40, 10% glycerol, 1 mM DTT, 1 mM NaF, and Mini Protease Inhibitors cocktail) and incubated with C2C12 (RRID: CVCL_0188) myotubes lysates overnight at 4 °C with rotation. Next day, the beads were washed three times with C2C12 cells wash buffer (50 mM Tris–HCl [pH 8.0], 150 mM NaCl, 0.1% NP-40, 1 mM DTT, 1 mM NaF, and Mini Protease Inhibitors cocktail), equilibrated with the TEV buffer (50 mM Tris–HCl [pH 8.0], 150 mM NaCl, 0.5% NP-40, 0.5 mM EDTA, 1 mM DTT, and 1 mM NaF) and proteins were eluted by incubating the beads in TEV buffer containing TEV protease (T4455, Sigma Aldrich) overnight in 4 °C. The eluted proteins were precipitated with Trichloroacetic acid, and the pellet was washed with ice-cold acetone. Precipitates were analyzed by SDS-PAGE and silver staining. The same samples were submitted to the MS Bioworks facility (USA) for protein identification.

### Immunoprecipitation

Synthetic biotinylated proteins were attached to Streptavidin-coupled Dynabeads M-280 (11205D, Life Technologies) in binding buffer (50 mM Tris–HCl, 150 mM NaCl, 50 mM NaH_2_PO_4_, 10 mM imidazole, 0.1% NP40, 10% glycerol, 10 mM β-mercaptoethanol, EDTA-free mini protease inhibitor cocktail (Roche), 1 mM phenylmethylsulphonyl fluoride, pervanadate, pH 8.0). After three washes with the same buffer, beads were incubated with purified proteins in the binding buffer or with an extract from HEK293 cells lysed with the binding buffer. After incubation, beads were washed three times with the binding buffer, and proteins were eluted in 2x Laemmli sample buffer with 50 mM DTT by boiling at 95 °C for 10 min. For co-immunoprecipitation experiments, Protein G Dynabeads (G 10004D, Invitrogen) were coated with the indicated antibodies according to the manufacturer’s protocol. Beads were incubated overnight at 4 °C with HEK293 cell extracts expressing the indicated proteins. After three washes with the lysis buffer, proteins were eluted in 2x Laemmli sample buffer with 50 mM DTT by boiling at 95 °C for 10 min. The precipitation of surface and total AChR from myotubes was performed as described previously (Bernadzki et al., 2017). For the precipitation of surface AChR, the myotubes were incubated with BTX-biotin (B1196, Invitrogen) for 5 min at 37 °C and washed three times with PBS, followed by lysis of myotubes in lysis buffer (100 mM Hepes pH 7.4, 150 mM NaCl, 1 mM PMSF, 0.5% NP-40, 1% CHAPS). For precipitation of total AChR, myotubes were lysed in lysis buffer and incubated with BTX-biotin for 30 min at 4 °C. NeutrAvidin beads (29,200, Thermo Fisher Scientific) were incubated with lysates overnight at 4 °C. The beads were washed three times in lysis buffer, and proteins were eluted by boiling in 2x Laemmli sample buffer for 10 min.

### Western blot

For Western blotting, lysates or protein precipitates were mixed with sample buffer, boiled for 10 min, resolved by SDS-PAGE, and transferred to nitrocellulose membranes (66,485, Pall Corporation) using Trans-Blot Turbo (1,704,270, Bio-Rad). Membranes were incubated in TBST (20 mM Tris–HCl, 150 mM NaCl [pH 7.6], and 0.1% Tween 20) containing 5% milk for 1 h, then incubated with antibodies in the blocking buffer overnight at 4 °C. The membranes were washed with TBST and incubated with appropriate horseradish peroxidase-conjugated secondary antibodies. The membranes were washed with TBST, developed with Clarity Chemiluminescent Substrate (1,705,060, Bio-Rad), imaged on photographic film (CL-XPosure, Thermo), and digitized using a scanner (Epson), or digitally acquired on the Bio-Rad ChemiDoc XRS Imaging System.

### Cell culture and transfection

C2C12 myoblasts were cultured and differentiated as described previously (Pezinski et al., 2020). Briefly, C2C12 cells (91031101, Sigma; RRID: CVCL_0188) were cultured on 0.2% gelatin-coated plates in Dulbecco’s modified Eagle’s medium (670087, DMEM, Gibco™) containing 20% fetal bovine serum supplemented with penicillin, streptomycin, and fungizone. Then, cells were differentiated on Petri dishes or 8-well Permanox chamber slides (177445, Sigma-Aldrich) precoated with a 10 μg/mL solution of laminin 111 (23017–015, Invitrogen) in differentiation medium (DMEM supplemented with 2% horse serum (26050088, Thermo Fisher Scientific). For the agrin-induced AChR clustering experiment, 8-well Permanox chamber slides were coated with 0.2% gelatin instead of laminin, and myotubes were treated with 10 nM recombinant rat Z’- spliced agrin (550-AG-100/CF, Biokom) for 12 h. For fluorescence AChR cluster analysis, differentiated cells were fixed for 7 min in 4% PFA at room temperature, washed in PBS, and blocked with 2% goat serum and 0.5% Triton X- 100 for 30 min. The myotubes were stained using Alexa Fluor 555-coupled bugarotoxin (B35451, Thermo Fisher Scientific) and Alexa Fluor 633 Phalloidin (A22284, Thermo Fisher Scientific) for F-actin. Cells were imaged on a Zeiss Cell Observer spinning-disk confocal microscope, and images were processed using Fiji (RRID: SCR_002285). For Western blot analysis and protein precipitation experiments, differentiated myotubes were lysed with ice-cold lysis buffer (50 mM Tris–HCl, 150 mM NaCl, 50 mM NaH_2_PO_4_, 10 mM imidazole, 0.1% NP40, 10% glycerol, protease inhibitor cocktail, and 50 mM DTT, pH 8.0), and the lysis suspension was incubated on a rotary shaker for 1 h at 4 °C and centrifuged at 13,000 x g at 4 °C. The supernatant was stored at −20 °C. For qRT-PCR, cells were treated with Trisure reagent (BIO-38032, Bioline), and RNA was isolated according to the manufacturer’s recommendations. The knockdown of SH3BP2 in C2C12 (91031101, Sigma; RRID: CVCL_0188) myotubes was performed as previously described (Gingras et al., 2016). Briefly, cells were transfected with siRNA targeting SH3BP2 or non-targeting control siRNA using Lipofectamine RNAiMAX (13778075, Invitrogen) on day three after differentiation induction, following the manufacturer’s recommendation. On day five after differentiation induction, cells were fixed with 4% PFA or treated with Trisure reagent (BIO-38032, Bioline) for qRT-PCR analysis, lysed with Lysis buffer for Western blot analysis, or subjected to coprecipitation experiments. HEK293 cells (CRL-1573, ATCC; RRID: CVCL_0045) were cultured in DMEM (670087, Gibco™) containing 10% fetal bovine serum supplemented with penicillin, streptomycin, and Fungizone. Transient transfections were performed with Lipofectamine 2000 (11668019, Invitrogen), according to the manufacturer’s instructions. HEK293 cells (CRL-1573, ATCC; RRID: CVCL_0045) were lysed using the same method as C2C12 cells.

### RNA extraction, reverse transcription, and PCR

The total RNA was extracted from myotubes using Trisure Reagent (BIO-38032, Bioline) following the manufacturer’s recommendations. About 1 μg of RNA was used for cDNA preparation using the High-Capacity cDNA Reverse Transcription Kit (4,374,966, Applied Biosystems). qRT-PCR was performed in three technical replicates using Fast SYBR Green Master Mix (4,309,155, Thermo Fisher Scientific) on the Step One Plus instrument (Thermo Fisher Scientific). For the qRT-PCR, the following primers were used: 5′- GAAGACTATGAGAAGGTGCCG −3′ and 5′- AAGATTCATCCC ACACGACC-3′ specific to SH3BP2, and 5′-GGCCTTCCGTGTTC CTAC-3′ and 5′-TGTCATCATACTTGGCAGGTT-3′ specific to GAPDH.

### Immunofluorescence microscopy

For immunostaining of muscle fibers, either mice were perfused with 4% PFA or isolated muscles were fixed in 4% PFA for 60 min at 4 °C. After washing in PBS, tissues were incubated with blocking buffer (2% BSA and 2% goat serum in PBS with 0.1% Triton X-100) for 30 min or 2 h in the case of embryonic diaphragms. The fibers were then incubated with primary antibodies in PBS with 0.1% Tween 20 (PBST) at 4 °C overnight, followed by three washes in PBST. Next, tissues were incubated at room temperature for 1 h with fluorescent secondary antibodies, followed by a 30 min incubation with BTX–A555 (B35451, Life Technologies; RRID: AB_2617152) Coverslips were mounted on slides with FluorSave reagent (Calbiochem) or Fluoromount mounting medium (F4680, Merck). For the Tibialis tissue cross-section, images were acquired using a spinning-disk confocal microscope (Zeiss). Images were processed with Fiji software (RRID: SCR_002285).

### *In vivo* electroporation

For electroporation of the Tibialis anterior muscle, mice were anesthetized with isoflurane inhalation and received an intraperitoneal injection of Metacam (0.4 mL/kg) twice daily for 3 days or Ketamine/Xylazine, and intraperitoneal injection of Buprenorphine 0.075 mg/kg was performed twice daily for 3 days. Anesthetized mice were injected with 30 μg of plasmid DNA (1 μg/μL in PBS) into the muscle using a Hamilton syringe. The muscle was electroporated with 10 pulses of electrical current (150 V/cm, 20 ms each) at 1 s intervals using an ECM-830 electroporator (45-0052INT, BTX Harvard Apparatus). Lidocaine gel was applied to the skin after electroporation. Mice were sacrificed 7 days after the electroporation.

### Synthetic peptides and recombinant proteins

The synthetic peptides corresponding to αDB1 sequence (Y713: DEST–Biotin–Ahx–TQPEDGNYENESVRQ-NH2; Y713-P: DEST–Biotin–Ahx–TQPEDGNpYENESVRQ-NH2; Y730: DEST–Biotin– Ahx–RQLENELQLEEYLKQKLQDE-NH2; and Y730P: DEST– Biotin–Ahx–RQLENELQLEEpYLKQKLQDE-NH2; where NH2 denotes peptide amidation, ‘p’ indicates phosphorylation of the tyrosine residue, and Ahx indicates a linker) were purchased from Lifetein. The peptides covering the cytoplasmic domain of AChRα (P1: Biotin-NTHHRSPSTHIMPEWVRKVFIDT; P2: Biotin-VFID TIPNIMFFSTMKRPSRDKQ; P3: Biotin-SRDKQEKRIFTEDIDISD ISGKP; P4: Biotin-ISGKPGPPPMGFHSPLIKHPEVK; P5: Biotin-HP EVKSAIEGVKYIAETMKSDQE; P6: Biotin-KSDQESNNAAEEWK YVAMVMDH) were purchased from Lifetein, USA. The GST-cytAChRα was purified from BL21 cells (RRID: CVCL_BL21). Transformed BL21 *E. coli* were grown in 200 mL of LB medium at 18 °C. The cells at an optical density between 0.4 and 0.6 were induced with 0.5 mM isopropyl 1-thio-β- galactopyranoside (IPTG) for 4 h at 18 °C. The bacteria were collected and lysed by sonication for 1 min in a high-salt buffer containing 500 mM NaCl, 25 mM Tris, pH 7.4, 5 mM DTT supplemented with protease inhibitors (11,873,580,001, Sigma) and phosphatase inhibitors (4,906,845,001, Sigma). The extract was then centrifuged at 10,000 g, and the supernatant was collected. About 30 μL of equilibrated glutathione beads (17–0756-01, GE Healthcare) were incubated with the supernatant overnight at 4 °C. After three washes with high salt buffer, proteins were eluted in elution buffer (10 mM glutathione, 500 mM NaCl, 25 mM Tris, pH 7.4). SH3BP2-Flag was expressed in HEK293 cells and purified using anti-FLAG agarose beads (A2220-5ML, Sigma-Aldrich) and eluted with FLAG peptide (F4799-4MG, Sigma-Aldrich) following the manufacturer’s recommendations.

### Muscle cross-sections, NMJ analysis, and creatine kinase activity

Mice were sacrificed by cervical dislocation, and the Tibialis anterior muscles were collected, embedded in OCT (05–9,801, Bio Optica), frozen in isopentane cooled with liquid nitrogen, and stored at −80 °C. Muscle 10 μm-thick cryosections were generated using a Leica CM 3050 S cryostat, collected on Ultra Plus microscope slides (10,149,870, Thermo Fisher Scientific), dried, and stored at −20 °C. For NMJ analysis, mice were either perfused with 4% PFA or dissected muscles were fixed in 4% PFA for 1 h at 4 °C. After washing in PBS, muscle fiber bundles were teased and stained with bungarotoxin to visualize AChR, Fasciculin II to visualize AChE, and with the indicated antibodies. Images of NMJs were acquired at 14-bit depth, 1002×1004 frame size, 63x magnification, 1x zoom, and a 0.27 μm z-stack interval and analyzed using the “NMJ-morph” ImageJ (RRID: SCR_003070) plugin (Jones et al., 2016). NMJ fragmentation was also confirmed by manual scoring. The blood was collected from male mice, allowed to clot by incubation at room temperature for 1 h, then centrifuged (10,000 rpm for 10 min at 4 °C). Serum was collected, and creatine kinase activity was analyzed with the Creatine Kinase Activity Assay Kit (MAK116-1KT, Sigma Aldrich) using the manufacturer’s recommendation.

### Grip strength test, voluntary running wheel, and treadmill experiment

Grip strength tests were performed as described before (Zhao et al., 2017). The strength was measured using a Grip Strength Meter (47,200, Ugo Basile) for all four paws (SH3BP2 mKO). Mice were allowed to grasp the grid connected to the meter, and gentle pulling was applied until the grid was released; the maximal force was recorded. The entire procedure was repeated 10 times per animal, with an interval of 1 min between repetitions. The mean force from 10 repetitions was normalized to body weight. For voluntary running, mice were placed in cages with upright running wheels (1800/50, Ugo Basile) for 12 h during the dark phase with free access to water and food. The distance run by each mouse was recorded. Treadmill performance, used to assess physical endurance, was performed as described before (Guo et al., 2020). Briefly, mice were familiarized with the treadmill (57,630, Ugo Basile) for three consecutive days. In the experiment, mice were placed on a treadmill and allowed to run at 10 m/min for 5 min. Subsequently, the speed was increased by 1 m/min every minute until the mice became exhausted, but no longer than 50 min. Exhaustion was defined as the mouse stopping for 15 s despite an aversive stimulus provided in the form of an air puff. The maximal speed, running time, and running distance were recorded at the end of the experiment.

### Muscle twitch and tetanic force measurement

The twitch and tetanic force measurements were performed using an Aurora Scientific 1300A device, as described before (Zhao et al., 2017). Mice (body weight 30 g-39 g) were anesthetized with isoflurane inhalation, followed by intraperitoneal injection of Buprenorphine (0.1 mg/kg), and placed on a 37 °C heating pad. The mice’s left knees were stabilized by clamping the knee, and the feet were fixed to the footplate connected to the transducer. The footplate angle was set to 17°. For muscle stimulation, the Tibialis anterior muscle was stimulated by placing a needle electrode subcutaneously and delivering 5 mA for 0.2 ms. Ten repetitions of stimulation and force recording were performed at 30 s intervals. For nerve stimulation, the sciatic nerve was surgically exposed and stimulated using two needle electrodes placed in close proximity. For the single twitch experiment, the nerve was stimulated at 5 mA for 0.2 ms, and the generated force was recorded in 10 repetitions at 30-s intervals. To determine tetanic force, the muscle or sciatic nerve was stimulated at 50, 100, and 125 Hz for 300 ms, with 10 repetitions at 2-min intervals.

### Electrophysiological recording

Electrophysiological recordings from the diaphragm-phrenic nerve preparations were performed as described before (Eiber et al., 2019). The diaphragm-phrenic nerve preparations were kept in Liley’s solution and gassed with 95% O_2_ and 5% CO_2_ at room temperature. At the beginning of the experiment, the compound muscle action potential (CMAP) was recorded using a micropipette with a tip diameter of approximately 10 μm filled with bathing solution. The electrode was then positioned 100 μm above the muscle surface, and the CMAP was recorded. Intracellular recordings were performed in a nerve-dependent (EPP) manner with electrodes introduced into single fibers. Two intracellular electrodes (resistance 10–15 MΩ) were inserted within 50 μm of the NMJs under visual inspection. At most NMJs, 50–100 spontaneous quantal events were recorded during 1 min. Decrements in EPPs were calculated using the mean of the first and last five recordings. pCLAMP 10 (RRID: SCR_011323) was used for recordings and analysis.

### Electron microscopy

For electron microscopy, mice were perfused in 4% paraformaldehyde with 1% glutaraldehyde. Tibialis anterior muscles were collected and stained with bungarotoxin to visualize AChR. Small muscle specimens enriched in NMJs were dissected under a fluorescent microscope. Collected muscle fragments were washed with PBS, post-fixed with 2.5% OsO_4_, stained *en bloc* with 2% uranium acetate, dehydrated in graded concentrations of ethanol, and embedded in Agar low viscosity resin (AGR1078, Agar Scientific). Semi-thin sections were cut using a glass knife, collected on glass slides, stained with 1% toluidine blue, and inspected with a light microscope to identify NMJs. Thin sections were cut from selected locations using a diamond knife and placed on copper grids for analysis using a STEM detector in a Zeiss Auriga SEM/FIB microscope with an accelerating voltage of 20 kV.

### Statistical analysis

GraphPad Prism version 10.0 (RRID: SCR_002798) was used to perform statistical analysis. Data were analyzed using an unpaired Student t-test (two tails) to compare two groups. For a multiple factorial analysis of variance, one-way or two-way ANOVA was used, unless indicated otherwise in a figure legend. Data were provided as mean ± SEM, unless otherwise indicated in the figure legend. A *p*-value <0.05 was considered statistically significant.

## Results

### SH3BP2 interacts with α-dystrobrevin-1 in a phospho-dependent manner

We previously reported a proteomics list of prospective αDB1-binding partners recruited to the phosphorylated Y713 of αDB1, and SH3BP2 scaffold protein (and its interacting adapter protein Grb2) were among them (Figure 1A; Gingras et al., 2016). SH3BP2 contains an N-terminal pleckstrin homology (PH) domain, a centrally located SH3-binding proline-rich region, and a C-terminal Src homology 2 (SH2) domain (Figure 1B). It also contains a predicted intrinsic disorder (ID) domain, suggesting potential self-interactions. The functions of SH3BP2 remain poorly characterized, and its role in skeletal muscle has not been investigated.

**Figure 1:**
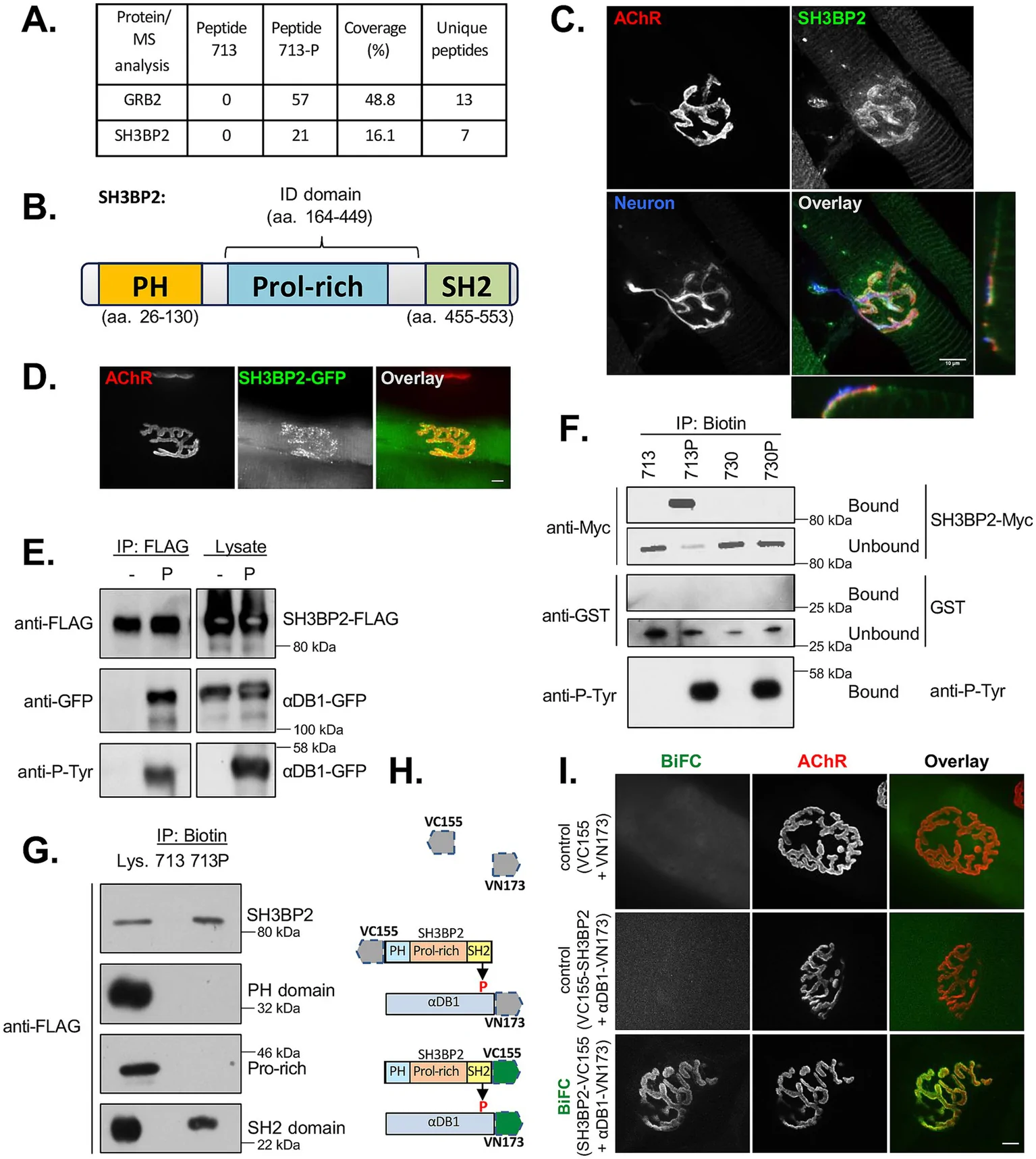
SH3BP2 interacts with phosphorylated αDB1 and localizes to the NMJ. **(A)** Mass spectrometry identifies SH3BP2 as an interactor of the phospho-Y713 peptide of αDB1 (Gingras et al., 2016). MS/MS analysis shows spectra for SH3BP2 and GRB2 identified using phosphorylated Y713-P and control Y713 peptides. **(B)** Schematic illustration of SH3BP2 domains. ID, intrinsically disordered domain. **(C)** SH3BP2 was localized to the pre- and postsynaptic compartments of the NMJ. Side views of XZ and YZ are also presented. The blue channel shows the motor neuron terminal (Neuron), visualized by combined neurofilament and synaptophysin immunofluorescence. Scale bar 10 μm **(D)**. Localization of SH3BP2-GFP at the NMJ in electroporated Tibialis muscle. Scale bar 10 μm. **(E)** SH3BP2-FLAG coprecipitates (IP) phosphorylated αDB1-GFP from HEK293 cell lysate in the presence of pervanadate (P). **(F)** Phosphorylated Y713 (Y713P) αDB1 peptide precipitates purified SH3BP2-MYC, suggesting a direct interaction. Unphosphorylated Y713, Y730, and phosphorylated 730-P peptides of αDB1 were used as negative controls. Purified GST provides unrelated protein control. Precipitation of anti-P-Tyr antibody was used to confirm peptide phosphorylation. **(G)** 713-P αDB1 peptide precipitates full-length SH3BP2-FLAG and the FLAG-tagged SH2 domain but not the PH domain or proline-rich domain (Pro-rich) of SH3BP2 from HEK293 cell extract. **(H,I)** A BiFC signal of SH3BP2–VC155 with αDB1–VN173 at the NMJ. Fluorescence complementation was observed when the two Venus fragments were fused C′-terminally to αDB1 and SH3BP2, close to the interaction sites. No BiFC was observed when the VC155 fragment was located at the N′-terminal of SH3BP2, demonstrating BiFC signal specificity. Scale bar 10 μm.

To study SH3BP2 localization in skeletal muscle, we immunostained teased fibers from the Tibialis anterior. Strong SH3BP2 immunoreactivity was detected at the NMJ postsynaptic machinery, which overlapped with AChR (Figure 1C). This pattern was similar to that previously reported for αDB1 (Peters et al., 1998). To verify SH3BP2 localization, we electroporated the Tibialis muscle with plasmids encoding GFP-SH3BP2 or GFP alone as a control. As expected, GFP did not localize to the NMJ (Supplementary Figure 1), but GFP-SH3BP2 was strongly enriched at the junctions, although in a more punctate pattern (Figure 1D).

To validate the interaction between SH3BP2 and phosphorylated αDB1, we performed a co-immunoprecipitation experiment of SH3BP2-FLAG and GFP-αDB1 overexpressed in HEK293 cells. To induce αDB1 phosphorylation, we co-transfected cells with a dominant-active Src kinase mutant and treated them with the phosphatase inhibitor pervanadate, as described previously (Gingras et al., 2016). As expected, SH3BP2-FLAG co-precipitated GFP-αDB1 from pervanadate-treated cell extract (Figure 1E). Next, we used biotinylated synthetic αDB1 peptides containing a centrally located tyrosine (Y713) in either phosphorylated or unphosphorylated states. We used the peptides to coat magnetic beads and tested their ability to precipitate purified SH3BP2-MYC protein. Only the phosphorylated Y713P peptide efficiently precipitated SH3BP2-MYC (Figure 1F). As a control, we performed a similar experiment with αDB1 peptides corresponding to the Y730 site; however, we observed no affinity for SH3BP2-MYC (Figure 1F). None of the peptides precipitated the purified GST protein, which served as an additional negative control. On the other hand, both Y713-P and Y730-P precipitated anti-phosphotyrosine antibody, indicating that they were phosphorylated (Figure 1F). These experiments demonstrate that SH3BP2 interacts directly and specifically with the phosphorylated form of Y713 of αDB1.

To examine which of the SH3BP2 domains is responsible for interaction with phosphorylated αDB1, we expressed full-length SH3BP2-FLAG and three domains of SH3BP2 (i.e., PH domain, a proline-rich domain, and SH2 domain) fused to FLAG individually in HEK293 cells and tested for their interaction with either phosphorylated or unphosphorylated Y713 αDB1 peptides. Only the SH2 domain with FLAG was precipitated by the Y713-P peptide of αDB1 (Figure 1G). This observation is consistent with the known function of SH2 domains, which mediate sequence-specific interactions with phosphorylated tyrosine residues. To confirm that the interaction between SH3BP2 and αDB1 also occurs in skeletal muscles, we performed bimolecular fluorescence complementation (BiFC) experiments. To this end, we electroporated the Tibialis muscle with plasmids expressing SH3BP2 and αDB1 fused to nonfluorescent parts of the Venus protein. If two proteins of interest interact and bring split Venus sequences into close proximity, the functional protein can fold and become fluorescent via bimolecular complementation (Aittaleb et al., 2017). As a control, we expressed fragments of Venus alone, but no BiFC signal was observed at the NMJ (Figures 1H,I). Similarly, no Venus fluorescence was detected when the VC155 tag was placed at the N-terminus of SH3BP2. In contrast, co-electroporation of SH3BP2 with the C-terminally attached VC155 and αDB1 with VN173 attached to the C-terminal side containing phosphorylated tyrosine residues led to a strong BiFC signal at the NMJ, which overlapped with AChR (Figures 1H,I). Therefore, SH3BP2 and αDB1 appear to interact at the NMJ.

To determine the SH3BP2 protein domains involved in NMJ localization, we electroporated the Tibialis muscle of WT mice with plasmids expressing GFP-tagged PH, Proline-rich, and SH2 domains or GFP-tagged truncation constructs missing the N-terminal PH domain or C′-terminal SH2 domain. Both truncation constructs were strongly enriched at the NMJ postsynaptic machinery (Figure 2). However, the protein missing the PH domain was more evenly distributed at the postsynaptic machinery, and the construct lacking the SH2 domain had a more punctate distribution. Additionally, fibers expressing the PH-proline-rich construct contained protein aggregates (Figure 2). Surprisingly, all three GFP-tagged individual domains of SH3BP2 also concentrated at the NMJ. The proline-rich domain showed the most even staining, the PH domain the most punctate staining, and the SH2 domain showed a relatively weak signal at the NMJ. Our results suggest that all three domains of the SH3BP2 adapter protein must interact with postsynaptic components that mediate their recruitment to the junction.

**Figure 2:**
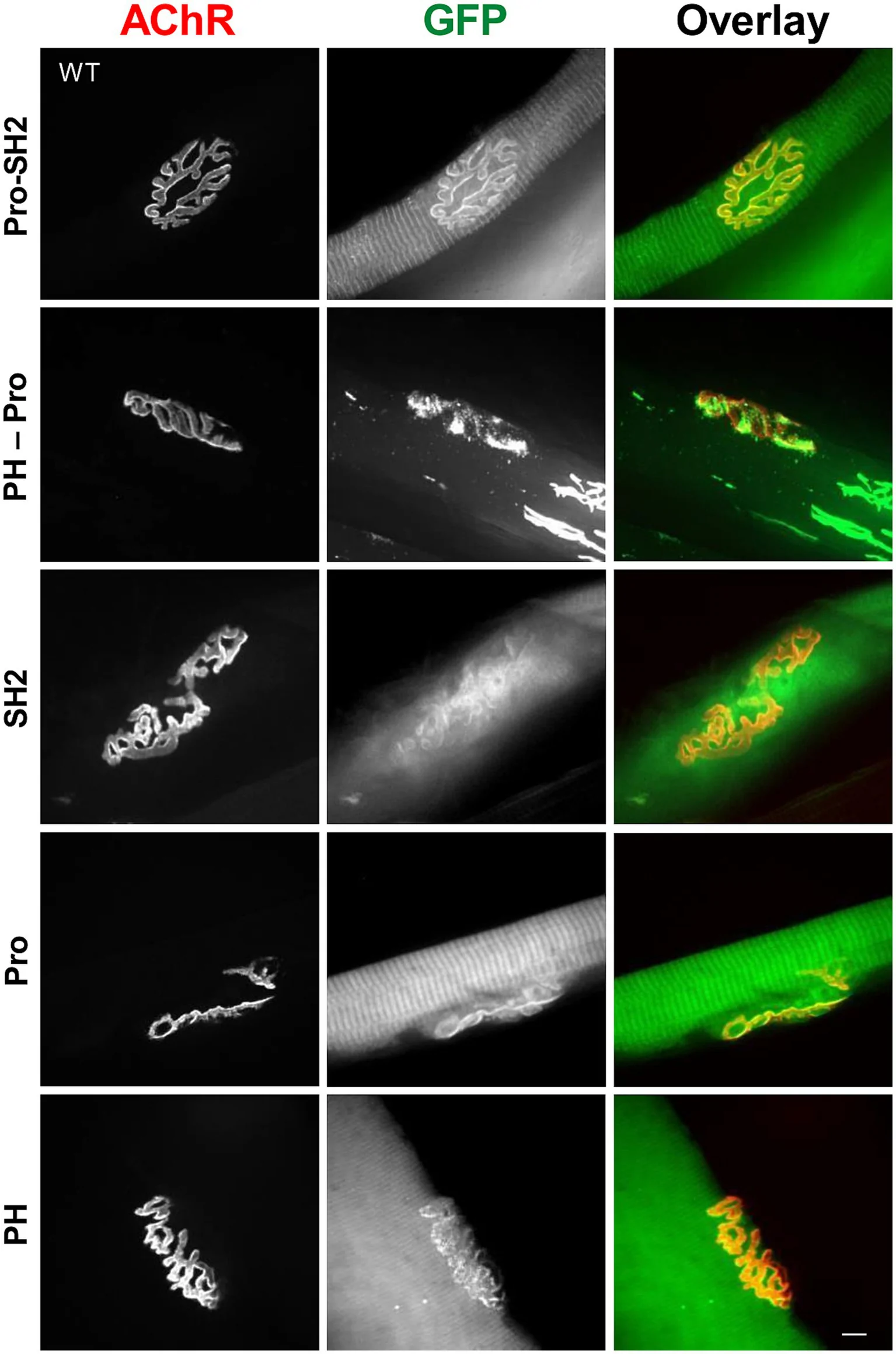
NMJ localization of SH3BP2 truncation proteins. Each SH3BP2 domain fused to GFP can be targeted to the junction. The Tibialis anterior muscles were electroporated with plasmids expressing GFP-tagged PH domain (PH), proline-rich domain (Pro), SH2 domain (SH2), or a truncated version of SH3BP2 protein missing either the SH2 domain (PH–Pro) or the PH domain (Pro–SH2). Scale bar 10 μm.

### Depletion of SH3BP2 impairs AChR clustering in cultured myotubes

Since SH3BP2 was localized along with αDB1 to the NMJ’s postsynaptic machinery, we examined whether SH3BP2 plays a role in the organization of AChRs. To this end, we knocked down SH3BP2 expression in C2C12 differentiated myotubes using siRNAs validated to reduce SH3BP2 mRNA and protein levels (Supplementary Figures 2A,B). To induce AChR clustering, myotubes were grown on laminin-111-coated surfaces or treated with soluble agrin. AChR clusters formed in the cultured myotubes represent many aspects of AChR assemblies *in vivo* (Bruneau et al., 2005a; Bruneau et al., 2005b; Gingras et al., 2016; Kummer et al., 2004). The control laminin-grown myotubes transfected with non-targeting siRNA formed a large number of small plaque-like AChR assemblies and many topologically complex, pretzel-shaped AChR clusters with F-actin-enriched podosomes (Proszynski et al., 2009) located at the sites of AChR perforations (Figure 3A). On the other hand, the agrin-treated control myotubes exhibit numerous small immature AChR clusters (Figure 3B). Depletion of SH3BP2 in the laminin-111-treated myotubes led to a significant reduction in both the number of complex clusters and the total number of AChR clusters (Figures 3A,C,D). SH3BP2 silencing in myotubes treated with agrin resulted in more than a 50% reduction in the total number of AChR clusters compared to the control (Figures 3B–D). A similar effect was apparent with another siRNA (Figures 3C,D), arguing against the off-target effects of the siRNAs. We hypothesized that impaired AChR clustering upon SH3BP2 depletion could result from decreased AChR production by myotubes, compromised exocytosis, or the cells’ inability to cluster AChR into larger assemblies. To test these three possibilities, we performed AChR precipitation experiments in which we labeled surface receptors by incubating live cells with biotin-conjugated bungarotoxin or by adding bungarotoxin to the cell homogenate, thereby labeling total AChR. The amounts of surface and total AChR subunits α and δ precipitated with streptavidin magnetic beads were comparable between the control and SH3BP2-depleted myotubes (Figure 3E). These results suggest that the absence of SH3BP2 does not affect AChR expression or its transport to the cell surface. Instead, myotubes lacking SH3BP2 exhibit a reduced ability to cluster receptor molecules at the plasma membrane.

**Figure 3:**
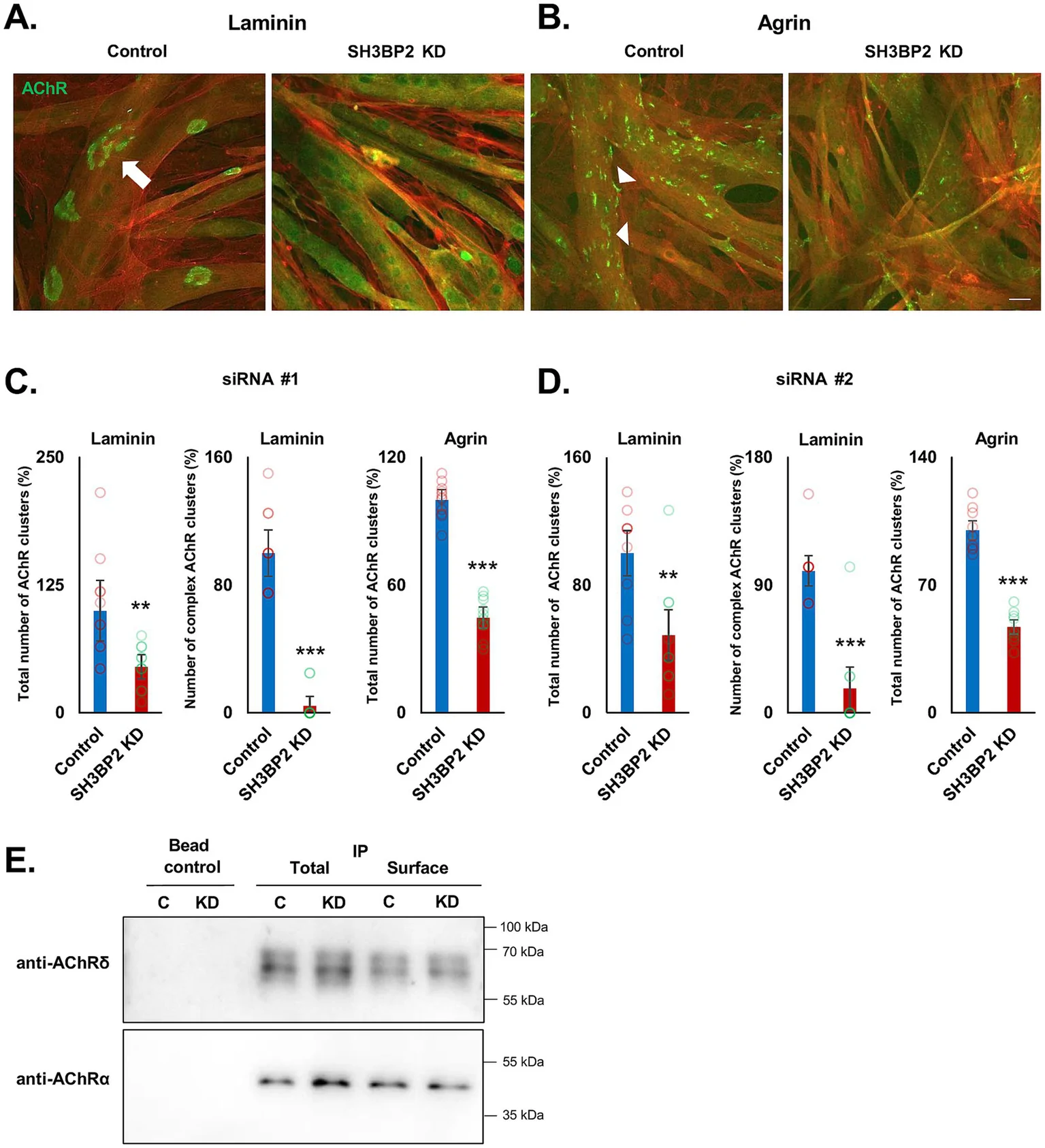
Depletion of SH3BP2 in myotubes affects AChR clustering. **(A,B)** Effect of SH3BP2 knockdown on AChR clustering in C2C12 myotubes grown on laminin-111 **(A)** or treated with soluble agrin **(B)**. The arrow indicates an AChR cluster with complex morphology, and arrowheads point to small clusters induced by agrin. F-actin, stained with phalloidin, is shown in red as a counterstain. Control—non-targeting siRNA. Scale bar 50 μm. **(C,D)** The quantification of the total number of AChR clusters and the number of complex clusters (for laminin-111) in myotubes transfected with scramble siRNA (control) and SH3BP2-targeting siRNA #1 **(C)** and #2 **(D)**. The data were normalized to control. Results are shown as mean ± SEM; two-tailed t-test; *n* = 3 for siRNA1 and *n* = 5 for siRNA2; **p < 0.01, ***p < 0.001 **(E)** Depletion of SH3BP2 in C2C12 myotubes did not affect the total AChR levels or surface AChR levels as detected by pull-down of AChR using biotin-bungarotoxin conjugates and neutravidin agarose. Beads control—precipitations from cell extract without bungarotoxin. C, control; KD, SH3BP2 knockdown.

### SH3BP2 interacts with DGC complexes and AChR subunits α and γ

Our results demonstrated that SH3BP2 localizes to the NMJ, interacts with αDB1, and regulates AChR clustering. To gain insight into the underlying mechanisms, we isolated SH3BP2-interacting proteins from differentiated myotubes and identified them by mass spectrometry. To this end, we coated magnetic beads with purified SH3BP2 and incubated them with a laminin-grown C2C12 myotube homogenate. After washing, the beads were suspended in sample buffer, and the isolated proteins were resolved by SDS-PAGE (Figure 4A) and identified by mass spectrometry. Among the identified proteins were several postsynaptic proteins, such as the α- and γ-subunits of the AChR, Low-density lipoprotein receptor-related protein 4 (Lrp4), and components of the dystroglycan complex, including utrophin, dystrophin, and α- and β-syntrophins (Figure 4B; Supplementary Table 1). To validate some of these interactions, we performed co-immunoprecipitation experiments. First, we sought to determine whether SH3BP2 interacts with identified DGC components. As shown in Figure 4C, syntrophin-1-GFP co-precipitated with SH3BP2-FLAG when the two proteins were expressed in HEK293 cells. Next, we extended our analysis to β-dystroglycan, which provides a scaffold for the assembly of the entire DGC. This protein was not detected by mass spectrometry among precipitated SH3BP2 interactors. However, β-dystroglycan is a transmembrane component of the DGC, and such proteins are often underrepresented in proteomics experiments due to solubilization problems. Our results show that β-dystroglycan-GFP co-precipitated SH3BP2-FLAG from HEK293 cell lysate (Figure 4D). Therefore, SH3BP2 appears to interact with several components of the DGC. This property of SH3BP2 was similar to that of its binding partner, the adapter protein Grb2, which has been shown to interact with β-dystroglycan, αDB1, and AChR (Colledge and Froehner, 1997; Gingras et al., 2016; Yang et al., 1995).

**Figure 4:**
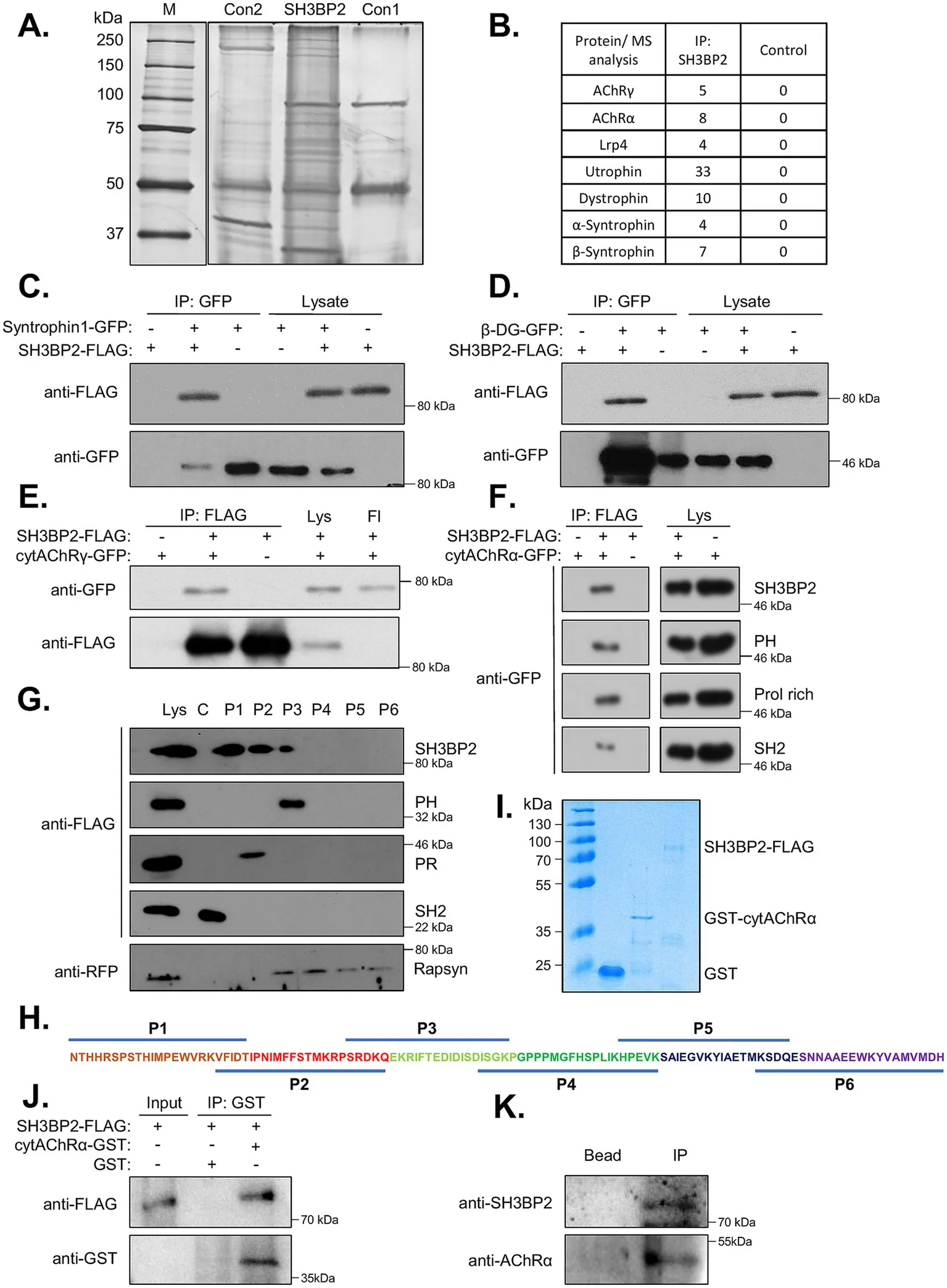
SH3BP2 interacts with syntrophin-1, β-dystroglycan, and AChR subunits. **(A)** Silver staining of the SDS-PAGE gel with proteins co-purified with SH3BP2 for mass spectrometry analysis. Con1 shows a control 1—TAP-tag-GFP-SH3BP2 purified from HEK293 cells without exposure to C2C12 lysate; Con 2 represents a control 2—IgG Dynabeads incubated with myotube extract without precoating with SH3BP2 protein; M, molecular weight marker. **(B)** Selected proteins that were specifically co-purified with TAP-tag-GFP-SH3BP2 and identified by MS/MS analysis. IP: SH3BP2 and Control columns show the number of peptides identified for each protein, indicated in the left column. The control column shows the cumulative number of peptides identified in both control samples. **(C)** Syntrophin-1 coprecipitates SH3BP2-FLAG from HEK293 cell extract. **(D)** A β-dystroglycan cytoplasmic domain fused to GFP coprecipitates SH3BP2-FLAG from a HEK293 cell homogenate. **(E)** SH3BP2-FLAG coprecipitates the cytoplasmic domain of AChR γ-subunit fused to GFP from HEK293 cell lysate. Lys, lysate of transfected HEK293 cells; Fl, unbound fraction. **(F)** SH3BP2-FLAG coprecipitates cytAChRα-GFP from HEK293 cell extract. Similarly, each domain: the PH domain (PH), proline-rich (PR), and SH2 domain (SH2) fused to FLAG coprecipitated cytAChRα from HEK293 cells lysate (Lys). **(G)** Coprecipitation of SH3BP2-FLAG, its individual domains fused to FLAG or Rapsyn-Cherry (positive control) from HEK293 cell extract with synthetic peptides covering the cytoplasmic domain of AChRα depicted in **(H)**. Biotinylated peptides were coupled to streptavidin Dynabeads and used to assess their ability to precipitate the indicated proteins. **(I)** Safe blue staining of purified GST, GST-cytAChRα, and SH3BP2-FLAG. **(J)** Coprecipitation experiment showing direct interaction of purified SH3BP2-FLAG protein with the GST-cytAChRα. Precipitation was performed using an anti-GST antibody, and GST precipitation in the presence of SH3BP2-FLAG served as a negative control. **(K)** Endogenous AChR coprecipitates endogenous SH3BP2 from the C2C12 myotubes lysate. Surface AChR was labeled in live C2C12 myotubes with biotin-bungarotoxin and precipitated from cell homogenates with neutravidin agarose. Beads—precipitates without bungarotoxin.

Our mass spectrometry analysis suggested interactions between SH3BP2 and AChR subunits α and γ. Since SH3BP2 is a cytoplasmic protein, its interaction should be mediated by the cytoplasmic domains of the receptor subunits. Each AChR subunit has four transmembrane domains (TMDs), with two terminal domains exposed to the extracellular environment and two cytoplasmic domains. However, the cytoplasmic loops between TMD I and II are believed to be too short to recruit any interacting proteins (seven and eight amino acids in mouse AChR α and γ subunits, respectively). Therefore, to test the interaction of SH3BP2 with AChR, we expressed in HEK293 cells the GFP-tagged cytoplasmic loops between TMD III and IV of AChRα (111 amino acids) and AChRγ (146 amino acids; named cytAChRα-GFP and cytAChRγ-GFP). Both proteins were co-precipitated by SH3BP2-FLAG from cell homogenates (Figures 4E,F). We decided to focus our subsequent analysis on the AChRα sequence, as it is the obligatory subunit present in two copies within the AChR pentamer. In contrast, the γ subunit is present in pentamers during embryonic development and is exchanged for the adult ε subunit in the postnatal life (Mishina et al., 1986). To determine which domain of SH3BP2 binds to AChRα, we performed co-immunoprecipitation assays using the cytAChRα-GFP and the PH, Proline-rich, and SH2 domains of SH3BP2 fused to FLAG. Interestingly, all three domains of the SH3BP2 protein interacted with cytAChRα (Figure 4F). This result is consistent with our previous observation that all three SH3BP2 domains can be recruited to the NMJ postsynaptic machinery independently (Figure 2). To map the binding sites on the AChRα sequence, we examined interactions between SH3BP2 and its domains with biotin-conjugated synthetic peptides that cover the entire AChRα cytoplasmic loop in a partially overlapping manner (Figures 4G,H). The full-length SH3BP2-FLAG protein was precipitated from HEK293 extract by magnetic streptavidin beads coated with peptides P1, P2, and P3 but not P4, P5, and P6, suggesting that the interaction with SH3BP2 occurs at the N′-terminal part of the cytAChRα (Figures 4G,H). Interestingly, each SH3BP2 domain was recruited by a different peptide: the SH2 domain by P1, the Proline-rich domain by P2, and the PH domain by P3 (Figure 4G). This suggests that SH3BP2 has three binding sites on AChRα, with each site interacting with distinct domains of SH3BP2. As a control, we assessed the peptides’ ability to precipitate mCherry-tagged rapsyn, which was also expressed in HEK293 cells. Rapsyn is a scaffold protein that directly interacts with AChRα(Lee et al., 2009). As expected, the rapsyn fusion protein was also precipitated by the peptides. However, its interaction was specific for peptides P3-P6, which cover the C-terminal fragment of the cytAChRα (Figures 4G,H).

To examine if the interaction between AChRα and SH3BP2 is direct, we purified GST or GST-cytAChRα from bacteria (Figure 4I) and SH3BP2-FLAG from HEK293 cells. We precipitated proteins from a mixed-protein solution using anti-GST-coated Dynabeads. As shown in Figure 4J, GST-cytAChRα, but not GST alone, coprecipitated SH3BP2-FLAG, suggesting a direct interaction between the two proteins. To confirm that SH3BP2 interacts with AChR in myotubes, we incubated live myotubes with biotin-conjugated bungarotoxin for 5 min to label surface AChRs. We then precipitated them using NeutrAvidin beads added to the cell lysate. Western blot analysis of isolated proteins showed that AChR co-precipitates endogenous SH3BP2 (Figure 4K).

Collectively, our experiments revealed that SH3BP2 binds multiple components of the DGC and that at least one interaction, with αDB1, is direct. Additionally, we showed that SH3BP2 binds to at least two AChR subunits and that its interaction with AChRα is direct and polyvalent.

### Abnormal NMJ organization in mice with SH3BP2 ablation in skeletal muscles

Next, we asked whether SH3BP2 regulates the NMJ postsynaptic machinery *in vivo*. We generated mice with SH3BP2 deletion in skeletal muscles by crossing SH3BP2 fl/fl mice with Myf5-Cre knock-in mice expressing Cre under the control of skeletal muscle-specific *Myf5* regulatory elements (Tallquist et al., 2000; Figure 5A).

**Figure 5:**
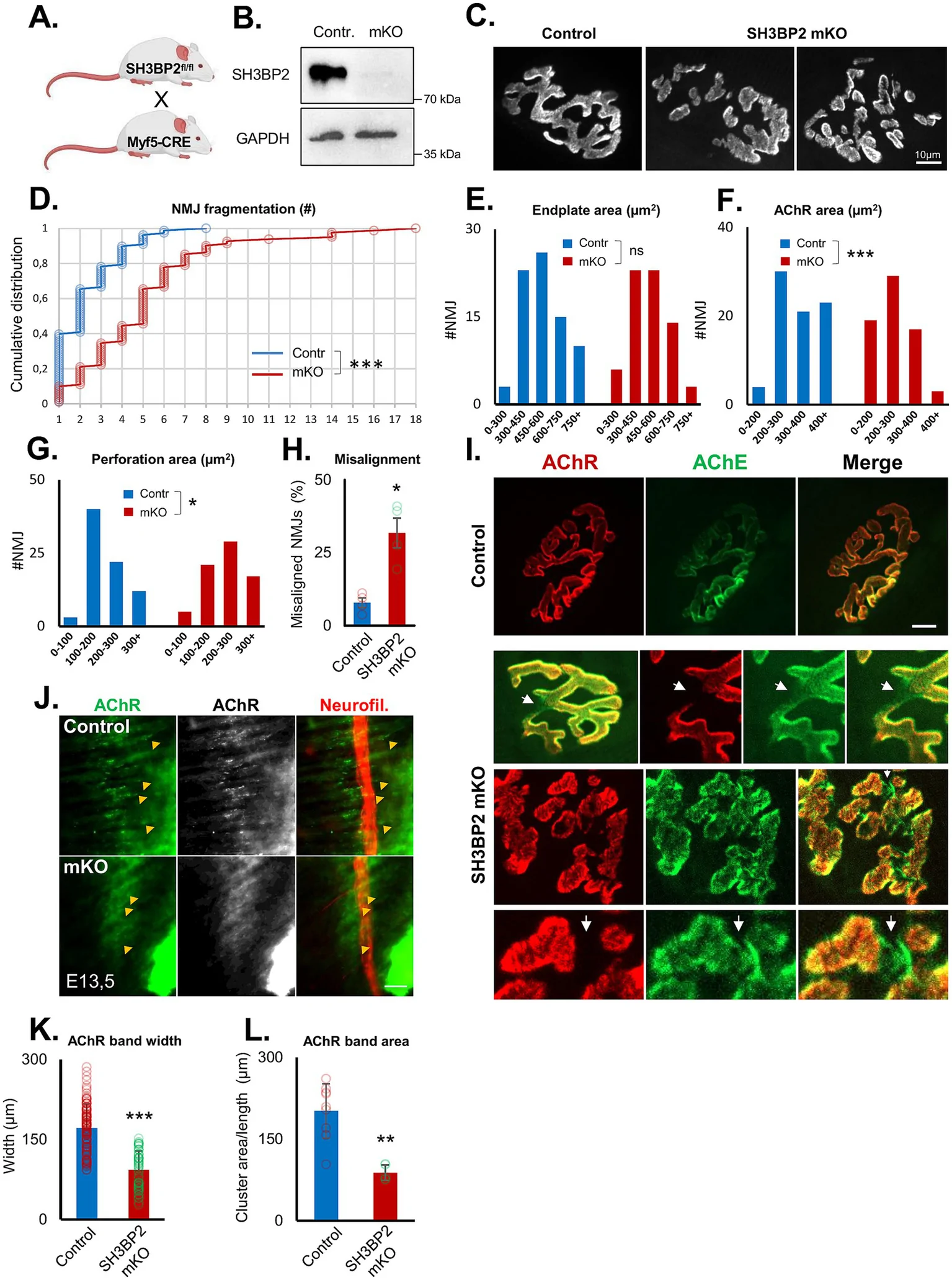
Abnormal NMJ organization in SH3BP2 mKO mice. **(A)** Strategy for generation of muscle-specific SH3BP2 knockout mice (mKO). Created in BioRender. Pradhan, B. (2026), https://BioRender.com/xr71usr. **(B)** Western blot analysis shows that SH3BP2 protein levels are substantially decreased in SH3BP2 mKO skeletal muscles. **(C)** Examples of NMJs in Soleus muscle from control and muscle-specific SH3BP2 KO mice. **(D)** Increased fragmentation of the NMJ postsynaptic machinery in the Soleus muscle of SH3BP2 mKO mice shown as the cumulative distributions (vertical axis) of NMJ fragments (horizontal axis). Kolmogorov–Smirnov test; *n* = 4 mice per genotype; more than 75 NMJs analyzed. **(E–G)** Histograms showing the distribution of various NMJ parameters from Soleus muscles (in μm^2^). **(E)** Endplate area, **(F)** AChR occupied area, and **(G)** perforation area of control and mSH3BP2 KO mice. Chi-square test; *n* = 4 mice per genotype; more than 65 NMJs analyzed. **(H)** Quantification of NMJs with misaligned AChR and AChE in Soleus muscle. Results are mean ± SEM; unpaired t-test; *n* = 4 mice per group; more than 100 NMJs were analyzed per genotype. **(I)** Misalignment of AChR and AChE in Soleus muscle in control (the upper panel) and SH3BP2 mKO mice (lower panels). Arrows show areas containing AChE without AChR. Scale bar 10 μm. **(J)** SH3BP2 mKO mice form fewer AChR clusters at E13,5 diaphragm. Arrows point to individual AChR aggregates. Neurofilament staining is shown in red to visualize axon bundles and small axonal processes approaching AChR clusters. Scale bar 50 μm. **(K)** Quantification of the width of the AChR cluster-containing band. **(L)** Quantification of the area occupied by AChR clusters. Results are mean ± SD; One-way ANOVA (K) and U Mann–Whitney test (L); *n* = 3 mice per group; *p < 0.05, **p < 0.0, ***p < 0.001.

Homozygous SH3BP2 fl/fl mice bearing the Myf5-Cre transgene (SH3BP2 mKO) were viable and born in accordance with the Mendelian ratio. Western blot analysis of Tibialis muscle homogenate revealed a substantial reduction of SH3BP2 expression (Figure 5B). The residual expression likely arises from non-muscle cells or incomplete excision of floxed SH3BP2 sequences.

Microscopy analysis of muscle fibers from SH3BP2 mKO mice revealed that neurofilament architecture of motor neurons was similar in control (SH3BP2 fl/fl) and SH3BP2 mKO mice (Supplementary Figure 3A). Quantification revealed that the number of axonal termini and the number of branching points at the junction, as well as axonal diameter, were normal in SH3BP2 mKO (Supplementary Figures 3B–D). However, NMJs appeared significantly more fragmented in mutant mice than in control animals (Figure 5C). To quantify potential synaptic alterations, we acquired approximately 80 NMJ images from the Soleus muscle of four animals per genotype and performed fragmentation analysis. This analysis confirmed that SH3BP2 ablation caused increased NMJ fragmentation in SH3BP2 mKO mice (Figure 5D). The NMJ (endplate) area in the Soleus muscles from SH3BP2 mKO mice was comparable to that of controls (Figure 5E). However, the junctional area occupied by AChR was significantly decreased (Figure 5F) in SH3BP2 KO muscles. The fraction of NMJs with the smallest AChR area (0–200 μm^2^) was increased, and the fraction with the highest receptor-rich domain (>400 μm^2^) was decreased in the mutant mice (Figure 5F). This effect was associated with an increase in the size of perforations between AChR-rich branches (Figure 5G). SH3BP2 mKO mice had a particularly reduced number of NMJs with a 100–200 μm^2^ perforation area and a predominance of junctions with larger areas between AChRs (Figure 5G). Similarly, NMJs in the Tibialis anterior and diaphragm muscles from SH3BP2 mKO were also more fragmented (Supplementary Figures 4A,B).

NMJ fragmentation may be associated with the gradual removal of AChRs from the synapse, thereby reducing the continuity of synaptic architecture. We therefore analyzed the localization of acetylcholinesterase (AChE), which the muscle releases into the synaptic cleft, where it associates with the ECM and therefore remains longer at the junction after other components are removed (Schmidt et al., 2011). Teased fibers from the Soleus muscle, stained with fasciculin II to visualize AChE and bungarotoxin to visualize AChR. WT mice showed good alignment between the two synaptic components (Figures 5H,I). In contrast, muscles from SH3BP2 mKO mice have discrete misaligned synaptic regions containing AChE without AChRs (Figure 5I). Quantitative analysis revealed that SH3BP2 mKO mice had almost four times as many NMJs with visible misalignment of synaptic components as control mice (Figure 5H). Interestingly, junctional areas with exposed AChE were observed in synapses with both more continuous and more fragmented architecture (Figure 5I). In many instances, recently removed AChRs caused clear discontinuities in synaptic branches, leading to an increase in NMJ fragments (Figure 5I).

Our experiments on molecular interactions, cell culture experiments on C2C12 myotubes, and adult NMJ analysis suggest that SH3BP2 contributes to AChR clustering. To further verify this, we analyzed postsynaptic machinery formation on embryonic day 13, 5 (E13,5). At this early developmental stage, skeletal muscle fibers spontaneously form small AChR clusters, called hot spots, in a cell-autonomous manner. Only at later developmental stages, some of these hot spots become innervated by approaching axons, leading to synapse formation, while others become dispersed (Tintignac et al., 2015). Therefore, AChR clustering at E13,5 resembles postsynaptic assemblies in cultured myotubes, which lack neuronal inputs. Interestingly, SH3BP2 mKO mice formed significantly fewer AChR hot spots that covered a smaller region of the diaphragm muscle at E13,5 (Figures 5J–L). Thus, we obtained additional evidence suggesting that SH3BP2 contributes to AChR clustering *in vivo* in a nerve-independent manner.

In line with other results, multiple NMJ abnormalities were also detected on P15, during intense postnatal synaptic remodeling; NMJs in SH3BP2 mKO mice exhibited reduced endplate size, AChR-occupied area, and synaptic perforations (Supplementary Figures 3E–H). These abnormalities suggest developmental delay caused by scaffold destabilization, thereby impairing AChR clustering, which is consistent with our previous findings.

Our findings of altered synaptic morphology in SH3BP2 mutants prompted us to examine NMJ ultrastructure using electron microscopy. Vertebrate NMJs have extended postsynaptic membranes with characteristic folds (Supplementary Figure 3I, purple color on the cross-section through a single NMJ branch). While AChRs are localized to postsynaptic densities at the crests of the folds, their depths contain highly clustered sodium channels. In response to a neurotransmitter, the AChR-triggered endplate potential is potentiated by sodium channels that initiate an action potential, leading to fiber contraction. As shown in Supplementary Figures 3I,J, mutant mice frequently exhibited exuberant postsynaptic folds with abnormal length and thickness. Additionally, postsynaptic density localization was often not limited to the crests of synaptic folds but was also observed in patches along the exuberant folds (Supplementary Figure 3I, lower-left panel). The width of the synaptic cleft was also significantly increased (Supplementary Figures 3I,J). Therefore, the absence of SH3BP2 leads to abnormalities in NMJ ultrastructure, in addition to general morphological impairments.

### Reduced muscle strength in the absence of SH3BP2

To determine whether synaptic defects in the absence of SH3BP2 are associated with impaired muscle strength, we assessed grip strength and running ability using voluntary running wheels and a treadmill. As shown in Figure 6A, SH3BP2 mKO mice had significantly lower grip strength and ran much less on the voluntary running wheels than SH3BP2 fl/fl (control) mice (Figures 6A,B). In the following experiments, we assessed animal fatigue on a treadmill operating at an accelerating speed. The experiment continued until the animals became exhausted, defined as refusing to run for 15 consecutive seconds despite an aversive stimulus (an air puff). SH3BP2 mKO mice become fatigued more quickly after running shorter distances and reach lower maximal speeds than control animals (Figures 6C–E).

**Figure 6:**
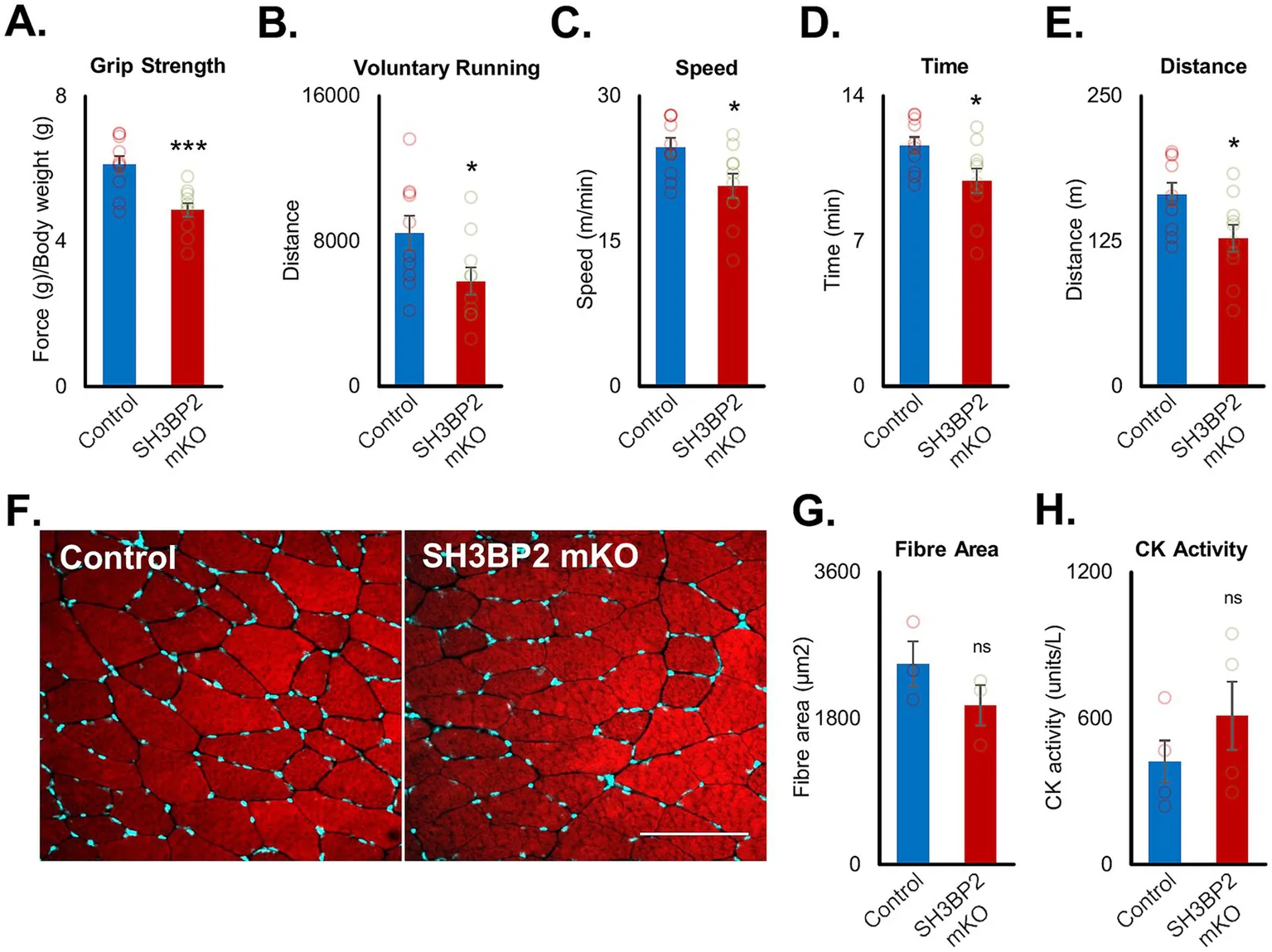
Reduced muscle strength but normal muscle fiber morphology in SH3BP2 mKO mice. **(A)** Decreased grip strength of SH3BP2 mKO mice. **(B)** Reduced voluntary running distance by SH3BP2 mKO mice in the running wheel experiment. **(C–E)** SH3BP2 mKO mice have reduced fitness in the treadmill experiment. Mutant mice ended the test reaching lower maximal speed **(C)**, shorter running time **(D)**, and distance **(E)**. Results are mean ± SEM; unpaired t-test; *n* = 10 mice per group. **(F)** Representative images of Tibialis anterior muscle cross sections stained with phalloidin for F-actin (red) and DAPI (cyan) to visualize nuclei. Quantification of **(G)** muscle fiber area. Results are mean ± SEM; unpaired t-test; *n* = 3 mice per group; **(H)** Creatine kinase (CK) activity in the sera from control and SH3BP2 mKO mice. Results are mean ± SEM; two-tailed Student’s t-test; *n* = 4 mice per group; *p < 0.05, ***p < 0.001.

Reduced muscle strength and animal fitness may reflect muscle atrophy; therefore, we performed histological analysis of Tibialis muscle cross-sections. General fiber morphology appeared normal in SH3BP2 mKO, and quantification of muscle fiber area revealed no significant differences compared to control animals (Figures 6F,G). Damaged muscle fibers release creatine kinase, thereby increasing serum creatine kinase levels. However, SH3BP2 mKO mice have serum creatine kinase levels comparable to those in control animals (Figure 6H). Collectively, our data strongly suggest that SH3BP2 deletion in skeletal muscles is not associated with muscle degeneration or injury, and that neuromuscular defects are likely a primary cause of muscle weakness.

### Impaired neuromuscular transmission in SH3BP2 mKO mice

To study neuromuscular transmission, we electrically stimulated the Tibialis anterior muscle (Figures 7A–D) or the sciatic nerve (Figures 7E–H) and measured the force generated by the hind paw of anesthetized mice. We found that the force generated by SH3BP2 mKO mice in response to single muscle stimulation at 5 mA for 0.2 ms was slightly decreased compared to SH3BP2 fl/fl (control) animals; however, the difference did not reach statistical significance (Figure 7B). When the Tibialis muscle was repetitively stimulated at 50, 100, and 125 Hz, the force generated during tetanic contraction was decreased at 50 and 100 Hz (Figures 7C,D). However, with sciatic nerve stimulation, when muscle contraction relies on NMJ transmission, a much greater and significant force decrement was observed in SH3BP2 mKO mice in a single twitch experiment (Figure 7F). Additionally, repetitive sciatic nerve stimulation led to a much weaker tetanic force in SH3BP2 mKO mice than in control animals (Figures 7G,H). The observed differences were statistically significant for all stimulation frequencies in this case. Collectively, our results suggest that SH3BP2 mKO mice may exhibit defects in twitch force and compromised neurotransmission at the NMJ.

**Figure 7:**
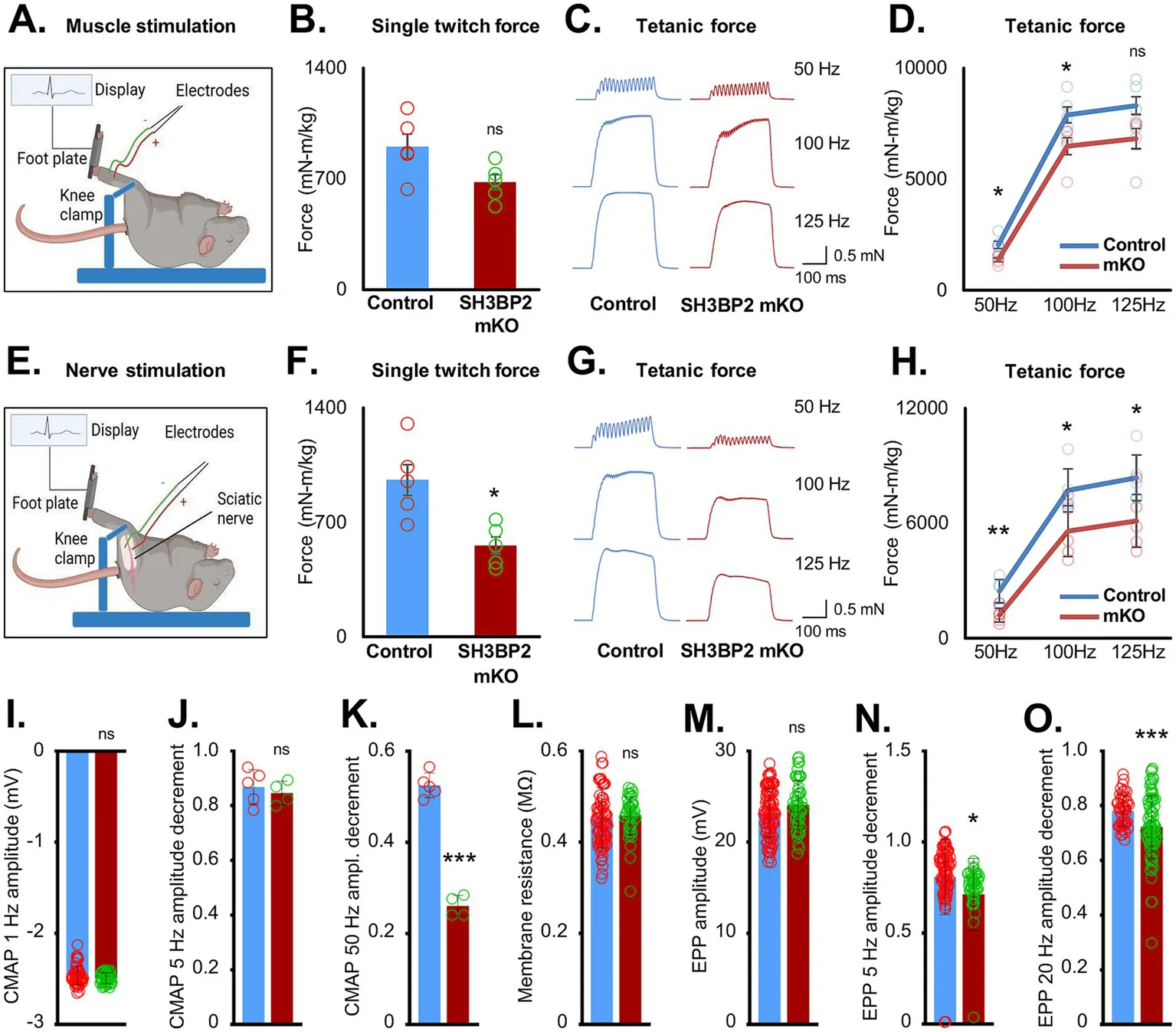
Impaired neuromuscular transmission in SH3BP2 mKO mice. **(A)** Schematic diagram of an experiment with the single twitch and tetanic force measurement in response to direct muscle stimulation. Created in BioRender. Pradhan, B. (2026), https://BioRender.com/y19sos2. **(B)** The single twitch force generated by control and SH3BP2 mKO mice in response to muscle stimulation at 5 mA for 0.2 ms. **(C)** Representative tetanic curves in response to direct muscle stimulation at 50, 100, and 125 Hz frequencies. **(D)** Quantification of tetanic force in response to direct muscle stimulation. All results are presented as mean ± SEM; unpaired t-test; *n* = 5 mice per group; **(E)** Schematic diagram of the experiment with twitch and tetanic force measurements in response to nerve stimulation. Created in BioRender. Pradhan, B. (2026), https://BioRender.com/mexacsf. **(F)** A significant reduction in single twitch force was observed in SH3BP2 mKO mice in response to sciatic nerve stimulation. **(G)** Representative tetanic curves in response to repetitive nerve stimulation at 50, 100, and 125 Hz frequencies. **(H)** In response to sciatic nerve stimulation, a significant reduction in generated tetanic force was observed in SH3BP2 mKO mice. All results are mean ± SEM; unpaired t-test; *n* = 5 mice per group. **(I–K)** CMAP analysis of diaphragm muscles revealed a significant change in decrement at 50 Hz stimulation in SH3BP2 mKO mice. **(L)** Muscle fiber membrane resistance was normal without SH3BP2. EPP amplitude was unaltered in a single stimulation experiment **(M)**, but repetitive stimulation at 5 Hz **(N)** and 20 Hz **(O)** revealed amplitude decrement in SH3BP2 mKO mice. Results shown in I-O are mean ± SEM; unpaired t-test; *n* = 4 mice for SH3BP2 mKO and *n* = 5 mice for control; *p < 0.05, **p < 0.0, ***p < 0.001.

To monitor the mechanical characteristics of neurotransmission defects in SH3BP2 mKO mice, we performed electrophysiological recordings on diaphragm muscles ex vivo. The compound muscle action potential (CMAP) in response to 1 and 5 Hz nerve stimulation was comparable between WT and mutant diaphragms (Figures 7I,J). However, substantial changes in the CMAP decrement were observed at 50 Hz stimulation in mutant mice (Figure 7K). Next, we performed single-fiber recordings. We first assessed the resistance of the muscle fiber membrane, which was normal in mutant mice (Figure 7L), suggesting that the muscle fiber sarcolemma was intact. This observation is in agreement with previous results on myonuclei localization, fiber cross-section area, and serum creatine kinase levels, all of which suggest no muscle fiber degeneration in SH3BP2 mKO mice. To characterize neuromuscular transmission, we also measured endplate potentials (EPP), which are depolarizations of the sarcolemma around endplates in response to nerve stimulation. EPP amplitude was normal in mutant mice (Figure 7M). However, repetitive stimulation at 5 Hz and 20 Hz revealed a significant decrease in the EPP decrement in SH3BP2 mKO mice, suggesting increased fatigability (Figures 7N,O). These defects might relate to structural NMJ impairments observed in mutant mice (Eiber et al., 2020; Figure 5; Supplementary Figures 3, 4).

## Discussion

Our previous proteomics studies identified the SH3BP2 scaffold protein as a potential interactor of αDB1 recruited to the phosphorylated tyrosine Y713 (Gingras et al., 2016). SH3BP2 has been shown to regulate signal transduction in immune and bone cells, and mutations in SH3BP2 cause Cherubism (Deckert et al., 1998; Levaot et al., 2011a). Nevertheless, its localization and function in skeletal muscles remain uninvestigated. This study demonstrates that SH3BP2 binds directly to phosphorylated αDB1 and that the SH2 domain mediates this interaction. Immunohistochemical analysis and muscle electroporation experiments confirmed that SH3BP2 localizes to the NMJ postsynaptic domain and, similarly to αDB1, co-localizes with AChR (Peters et al., 1998). Our BiFC experiments suggest that SH3BP2 interacts with αDB1 at the NMJ, where αDB1 has been shown to concentrate (Peters et al., 1998). Unexpectedly, the SH2 domain appeared dispensable for SH3BP2 synaptic localization. Moreover, each of the three SH3BP2 domains targeted GFP to the NMJ. These results suggested that all three SH3BP2 domains interact with the postsynaptic components. Our proteomics experiment identified four DGC components as candidate SH3BP2-interacting proteins. We provide further confirmation that SH3BP2 interacts with syntrophin-1, β-dystroglycan, and αDB1. Therefore, SH3BP2 exhibits polyvalent interactions with the DGC. Additionally, our experiments showed that SH3BP2 binds AChR subunits α and γ and that at least the interaction with AChRα is direct. Interestingly, our domain-mapping experiments revealed that all three SH3BP2 domains can interact with AChRα at three distinct sites. This observation is consistent with our imaging data, showing that all three SH3BP2 domains are recruited to the NMJ. Thus, SH3BP2 exhibits polyvalent binding also to the AChR pentamers, which contain two copies of the α subunit. Additional evidence for SH3BP2’s role in AChR clustering comes from *in vitro* KD experiments showing that SH3BP2-depleted myotubes failed to cluster AChRs despite normal plasma membrane expression. Consistently, impaired AChR cluster formation was observed *in vivo* at E13.5, before spontaneously formed clusters are innervated by motor neuron axons. Multiple NMJ morphology parameters, such as endplate area, AChR area, and perforation area, were also abnormal at the juvenile stage (P15), when NMJs undergo intense postnatal remodeling. At steady state, adult SH3BP2 mKO mice exhibit increased NMJ fragmentation and misalignment of AChRs relative to AChE, suggesting increased receptor removal from synaptic regions. Similarly, ultrastructural analysis revealed multiple alterations in NMJ organization, including increased lengths and widths of synaptic folds and widths of synaptic clefts. These synaptic morphological alterations could impair synaptic transmission, muscle strength, and animal fitness. Subsequent twitch force analysis following electrical stimulation showed that SH3BP2 mKO mice exhibit a defect in muscle force generation, suggesting impaired neuromuscular transmission as the primary cause. Electrophysiological recordings confirmed this, revealing CMAP and EPP defects at higher stimulation frequencies. Normal single-pulse endplate potentials (EPPs) in SH3BP2 mKO mice indicate that basal neuromuscular transmission remains intact. However, upon repetitive stimulation, we observed a ∼ 50% increase in CMAP decrement at 50 Hz and a modest increase in EPP decrement at 20 Hz, revealing vulnerability during high-frequency activity. These findings indicate that SH3BP2 plays a critical role in sustaining synaptic efficacy under demand, likely by promoting postsynaptic structural integrity, which is important during prolonged activation. This susceptibility to fatigue under load parallels phenotypes observed in other NMJ organizers and underscores SH3BP2’s contribution to synaptic robustness rather than to elementary neurotransmission. Future studies employing aging cohorts or disease models will be essential to delineate these activity-dependent deficits. Our behavioral analysis further demonstrated that SH3BP2 mKO mice have reduced grip strength and running ability, as well as increased fatigue. At the same time, muscle fiber integrity appears to be normal in mutant mice, which strongly argues for NMJ-specific rather than general muscle defects. First, muscle histology has not revealed signs of muscle fiber degeneration. Second, blood creatine kinase levels were normal. Third, electrophysiological measurements showed normal membrane resistance in SH3BP2 mKO muscles.

Our study demonstrates that SH3BP2 interacts peripherally with several DGC proteins. Lack of function in several DGC components, e.g., dystroglycan, dystrophin, and sarcoglycans, leads to muscular dystrophies (Grady et al., 1997a; Kameya et al., 1999). These are integral components required to assemble the entire DGC. The loss of function of other DGC proteins affects the NMJ without causing the symptoms of muscular dystrophy. For example, knockout of syntrophin-1 or utrophin does not cause muscular dystrophy (Grady et al., 1997a; Kameya et al., 1999). Similarly, lack of SH3BP2 does not cause muscular dystrophy symptoms. The DGC components also regulate the organization of synaptic folds. For instance, dystrophin mutations or deletion of utrophin or αDB1 leads to reduced synaptic folds (Banks et al., 2009; Deconinck et al., 1997; Grady et al., 2000). SH3BP2 mKO mice have increased synaptic fold length and partial mislocalization of electron-dense postsynaptic machinery along exuberant folds. This phenotype was observed in mice lacking αDB1 or with reduced rapsyn expression and was interpreted as a compensatory mechanism for compromised AChR clustering (Martinez-Martinez et al., 2009; Grady et al., 2000).

In addition to NMJ enrichment, SH3BP2 was also detected along the entire fibers in a striated pattern. This pattern resembles DGC-enriched costameres, which connect contractile machinery with the sarcolemma. Because SH3BP2 interacts with multiple DGC components, it may function more broadly as a scaffold linking DGC signaling complexes outside the synapse. Whether SH3BP2 contributes to costamere organization or mechanotransduction remains to be investigated.

Our previous work identified that podosomes, dynamic, actin-rich structures, regulate developmental remodeling of AChR clusters in laminin-cultured myotubes (Proszynski et al., 2009). Podosomes and closely related invadopodia are protrusive organelles that degrade extracellular matrix and promote cell migration, including cancer metastasis. Interestingly, SH3BP2 has recently been reported to associate with podosomes in macrophages (Arden et al., 2025). Our proteomics analysis suggests that SH3BP2 may interact with several podosome-associated proteins, including cortactin, talin, utrophin, filamins, and ezrin, all of which have recognized roles in actin remodeling and podosome organization. It would be interesting to study whether SH3BP2 is also associated with podosomes *in vivo* and what role it plays in their organization. However, there is no clear evidence for podosome association with the NMJ *in vivo*.

Our experiments also identified that SH3BP2 interacts with Lrp4, which, together with MuSK, serves as a receptor for nerve-derived agrin. Its activation triggers signaling that induces synaptic gene expression and postsynaptic development. Therefore, SH3BP2 likely plays multiple roles at the NMJ, and further studies should aim to more comprehensively identify and characterize those functions. Further studies should also address SH3BP2 functions in aging and pathological conditions.

## Data Availability

The original contributions presented in the study are included in the article/Supplementary material, further inquiries can be directed to the corresponding author.

## Supplementary material

SUPPLEMENTARY TABLE 1 List of proteins interacting with SH3BP2 identified by mass spectrometry analysis.

SUPPLEMENTARY TABLE 2 List of primary antibodies used in the study.

SUPPLEMENTARY FIGURE 1 Control for muscle electroporation. A representative image showing the Tibialis anterior electroporated with a plasmid expressing GFP, a negative control for SH3BP2 localization experiments. Scale bar 10 μm.

SUPPLEMENTARY FIGURE 2 Control for siRNA-mediated silencing of SH3BP2. **(A)** Validation of SH3BP2 knockdown at the mRNA level using qRT-PCR and **(B)** at the protein level using Western blot. C2C12 myotubes were transfected with either siRNAs targeting SH3BP2 or a scrambled, non-targeting siRNA (neg. ctrl.). Results are mean ± SEM; one-way ANOVA; *n* = 3 for negative control and siRNA1; *n* = 4 for siRNA2 group; *n* = 2 for untransfected; ***p* < 0.0, ****p* < 0.001.

SUPPLEMENTARY FIGURE 3 Synaptic abnormalities in SH3BP2 mKO mice. **(A)** Representative images of axonal morphology at NMJs in SH3BP2 fl/fl (Control) and SH3BP2 mKO mice. Scale bar 5 μm. **(B)** Quantification of the number of axonal terminals at the NMJ. **(C)** Quantification of axonal branching points at the NMJ. **(D)** Quantification of the axonal diameter. Results are mean ± SD; U Mann-Whitney test; *n* = 3 mice per group. **(E)** Representative image of AChRs in P15 Tibialis anterior muscles from control and SH3BP2 mKO mice. Scale bar 10 μm. Quantification of endplate areas **(F)**, AChR-occupied areas **(G)**, and perforation areas **(H)** in Tibialis anterior muscles at P15. Results are mean ± SD; U Mann-Whitney test; *n* = 3 mice per group; **(I)** Representative electron microscopy images of NMJs in Tibialis anterior muscles showing increased length and width of junctional folds in SH3BP2 mKO. For better visibility, synaptic folds were color-coded in purple in the image of the single NMJ branch cross-section from the control NMJ; blue N indicates the neuronal terminal. Mutant mice also exhibit mislocalization of electron-dense structures from the crests into the depths of exuberant folds (lower-left image). Scale bars were 1 μm (left column) and 200 μm (right column). **(J)** Quantification of the various NMJ parameters from electron microscopy analysis. The length and width of synaptic folds, as well as the width of the synaptic cleft, are larger in SH3BP2 mKO mice. However, the number of folds per endplate was not affected. Results are mean ± SD; unpaired Student’s t-test; *n* = 4 mice per group; **p* < 0.05, ***p* < 0.0, ****p* < 0.001.

SUPPLEMENTARY FIGURE 4 NMJ fragmentation in Tibialis and diaphragm muscles. **(A)** Increased NMJ fragmentation in the Tibialis anterior showed as a difference in the cumulative distribution (vertical axis) of the number of fragments (horizontal axis) in mKO mice. Kolmogorov-Smirnov test; *n* = 8 mice per genotype; more than 200 NMJs analyzed. **(B)** Increased NMJ fragmentation in the diaphragm showed as a difference in the cumulative distribution (vertical axis) of the number of fragments (horizontal axis) in mKO mice. Kolmogorov Smirnov test *n* = 4 mice per genotype; more than 80 NMJs analyzed; ****p* < 0.001.
