# Supplementary material for: SH3BP2 deficiency in skeletal muscle leads to neuromuscular junction abnormalities and decreased muscle strength
Source: Front Mol Neurosci. 2026 Oct 5;19:1985823. doi: 10.3389/fnmol.2026.1985823 (PMC13677847; doi:10.3389/fnmol.2026.1985823)
Supplement: Supplementary file 1 [file Data_Sheet_1.PDF]

Supplementary Table 1

Identified Proteins	Accession Number	Molecular Weight	# of identified peptides SH3BP2	Control
Titin OS=Mus musculus GN=Ttn PE=1 SV=1	sp|A2ASS6|TITIN_MOUSE	3906 kDa	1473	313
Desmin OS=Mus musculus GN=Des PE=1 SV=3	sp|P31001|DESM_MOUSE	53 kDa	1041	424
Filamin-C OS=Mus musculus GN=Flnc PE=1 SV=3	sp|Q8VHX6|FLNC_MOUSE	291 kDa	842	233
Myosin-XVIIIa OS=Mus musculus GN=Myo18a PE=1 SV=2	sp|Q9JMH9|MY18A_MOUSE	233 kDa	518	183
Fibronectin OS=Mus musculus GN=Fn1 PE=1 SV=3	sp|P11276|FINC_MOUSE	272 kDa	376	73
Triple functional domain protein OS=Mus musculus GN=Trio PE=1 SV=3	sp|Q0KL02|TRIO_MOUSE	348 kDa	370	4
Microtubule-actin cross-linking factor 1 OS=Mus musculus GN=Macf1 PE=1 SV=2	sp|Q9QXZ0|MACF1_MOUSE	832 kDa	331	48
Moesin OS=Mus musculus GN=Msn PE=1 SV=3	sp|P26041|MOES_MOUSE	68 kDa	191	83
Epiplakin OS=Mus musculus GN=Eppk1 PE=1 SV=2	sp|Q8R0W0|EPIPL_MOUSE	725 kDa	266	23
Collagen alpha-1(XII) chain OS=Mus musculus GN=Col12a1 PE=2 SV=3	sp|Q60847|COCA1_MOUSE	340 kDa	235	0
Filamin-A-interacting protein 1 OS=Mus musculus GN=Filip1 PE=1 SV=2	sp|Q9CS72|FLIP1_MOUSE	138 kDa	177	4
Kinesin-1 heavy chain OS=Mus musculus GN=Kif5b PE=1 SV=3	sp|Q61768|KINH_MOUSE	110 kDa	142	39
Talin-1 OS=Mus musculus GN=Tln1 PE=1 SV=2	sp|P26039|TLN1_MOUSE	270 kDa	107	56
Nucleolin OS=Mus musculus GN=Ncl PE=1 SV=2	sp|P09405|NUCL_MOUSE	77 kDa	107	40
Dedicator of cytokinesis protein 7 OS=Mus musculus GN=Dock7 PE=1 SV=3	sp|Q8R1A4|DOCK7_MOUSE	241 kDa	112	34
Coatomer subunit alpha OS=Mus musculus GN=Copa PE=1 SV=2	sp|Q8CIE6|COPA_MOUSE	138 kDa	116	23
Filamin-C OS=Homo sapiens GN=FLNC PE=1 SV=3	sp|Q14315|FLNC_HUMAN	291 kDa	174	0
Src substrate cortactin OS=Mus musculus GN=Cttn PE=1 SV=2	sp|Q60598|SRC8_MOUSE	61 kDa	61	34
Filamin-A OS=Mus musculus GN=Flna PE=1 SV=5	sp|Q8BTM8|FLNA_MOUSE	281 kDa	113	57
Collagen alpha-1(III) chain OS=Mus musculus GN=Col3a1 PE=2 SV=4	sp|P08121|CO3A1_MOUSE	139 kDa	87	3
Basement membrane-specific heparan sulfate proteoglycan core protein OS=Mus mu	sp|Q05793|PGBM_MOUSE	398 kDa	112	0
Serine/threonine-protein kinase DCLK1 OS=Mus musculus GN=Dclk1 PE=1 SV=1	sp|Q9JLM8|DCLK1_MOUSE	84 kDa	72	14
Rho guanine nucleotide exchange factor 2 OS=Mus musculus GN=Arhgef2 PE=1 SV=4	sp|Q60875|ARHG2_MOUSE	112 kDa	67	23
Fragile X mental retardation syndrome-related protein 1 OS=Mus musculus GN=Fxr1	sp|Q61584|FXR1_MOUSE	76 kDa	80	4
60S ribosomal protein L6 OS=Mus musculus GN=Rpl6 PE=1 SV=3	sp|P47911|RL6_MOUSE	34 kDa	109	0
Cold shock domain-containing protein E1 OS=Mus musculus GN=Csde1 PE=2 SV=1	sp|Q91W50|CSDE1_MOUSE	89 kDa	78	5
Serine/threonine-protein kinase TAO1 OS=Mus musculus GN=Taok1 PE=1 SV=1	sp|Q5F2E8|TAOK1_MOUSE	116 kDa	63	12
Calsequestrin-2 OS=Mus musculus GN=Casq2 PE=2 SV=3	sp|O09161|CASQ2_MOUSE	48 kDa	54	7
Elongation factor 2 OS=Homo sapiens GN=EEF2 PE=1 SV=4	sp|P13639|EF2_HUMAN	95 kDa	92	0
Muscle-related coiled-coil protein OS=Mus musculus GN=Murc PE=2 SV=1	sp|A2AMM0|MURC_MOUSE	41 kDa	49	12
Vacuolar protein sorting-associated protein 35 OS=Mus musculus GN=Vps35 PE=1 SV	sp|Q9EQH3|VPS35_MOUSE	92 kDa	51	10
Transcriptional activator protein Pur-beta OS=Mus musculus GN=Purb PE=1 SV=3	sp|O35295|PURB_MOUSE	34 kDa	49	11
Myomesin-3 OS=Mus musculus GN=Myom3 PE=2 SV=1	sp|A2ABU4|MYOM3_MOUSE	162 kDa	53	5
AP-2 complex subunit mu OS=Mus musculus GN=Ap2m1 PE=1 SV=1	sp|P84091|AP2M1_MOUSE (+1)	50 kDa	45	12
Titin OS=Homo sapiens GN=TTN PE=1 SV=2	sp|Q8WZ42|TITIN_HUMAN	3816 kDa	829	176
Phosphoglucomutase-like protein 5 OS=Mus musculus GN=Pgm5 PE=1 SV=2	sp|Q8BZF8|PGM5_MOUSE	62 kDa	42	11
Syncoilin OS=Mus musculus GN=Sync PE=1 SV=1	sp|Q9EPM5|SYNCI_MOUSE	54 kDa	47	7
E3 ubiquitin-protein ligase HECTD1 OS=Mus musculus GN=Hectd1 PE=1 SV=2	sp|Q69ZR2|HECD1_MOUSE	290 kDa	88	0
Tubulin beta-2A chain OS=Homo sapiens GN=TUBB2A PE=1 SV=1	sp|Q13885|TBB2A_HUMAN (+1)	50 kDa	87	0
Calponin-3 OS=Mus musculus GN=Cnn3 PE=2 SV=1	sp|Q9DAW9|CNN3_MOUSE	36 kDa	39	9
Radixin OS=Mus musculus GN=Rdx PE=1 SV=3	sp|P26043|RADI_MOUSE	69 kDa	79	32
Myosin-X OS=Homo sapiens GN=MYO10 PE=1 SV=3	sp|Q9HD67|MYO10_HUMAN	237 kDa	45	2
Sorbin and SH3 domain-containing protein 2 OS=Mus musculus GN=Sorbs2 PE=1 SV=	sp|Q3UTJ2|SRBS2_MOUSE	132 kDa	82	0
Obscurin-like protein 1 OS=Homo sapiens GN=OBSL1 PE=1 SV=3	sp|O75147|OBSL1_HUMAN	153 kDa	40	5
Serine-threonine kinase receptor-associated protein OS=Mus musculus GN=Strap PE=	sp|Q9Z1Z2|STRAP_MOUSE	38 kDa	35	11
Tubulin beta-3 chain OS=Homo sapiens GN=TUBB3 PE=1 SV=2	sp|Q13509|TBB3_HUMAN (+1)	50 kDa	71	0
Regulator of nonsense transcripts 1 OS=Mus musculus GN=Upf1 PE=1 SV=2	sp|Q9EPU0|RENT1_MOUSE	124 kDa	40	4
40S ribosomal protein S6 OS=Homo sapiens GN=RPS6 PE=1 SV=1	sp|P62753|RS6_HUMAN (+1)	29 kDa	71	0
60S ribosomal protein L7a OS=Mus musculus GN=Rpl7a PE=2 SV=2	sp|P12970|RL7A_MOUSE (+1)	30 kDa	66	0
Ezrin OS=Mus musculus GN=Ezr PE=1 SV=3	sp|P26040|EZRI_MOUSE	69 kDa	67	24
DNA topoisomerase 1 OS=Mus musculus GN=Top1 PE=1 SV=2	sp|Q04750|TOP1_MOUSE	91 kDa	64	0
Procollagen-lysine,2-oxoglutarate 5-dioxygenase 2 OS=Mus musculus GN=Plod2 PE=2	sp|Q9R0B9|PLOD2_MOUSE	84 kDa	28	13
Nebulin OS=Homo sapiens GN=NEB PE=1 SV=4	sp|P20929|NEBU_HUMAN	773 kDa	55	0
Catenin delta-1 OS=Mus musculus GN=Ctnnd1 PE=1 SV=2	sp|P30999|CTND1_MOUSE	105 kDa	50	0
Alanine aminotransferase 2 OS=Mus musculus GN=Gpt2 PE=2 SV=1	sp|Q8BGT5|ALAT2_MOUSE	58 kDa	49	0
40S ribosomal protein S2 OS=Mus musculus GN=Rps2 PE=1 SV=3	sp|P25444|RS2_MOUSE	31 kDa	48	0
Fibronectin OS=Homo sapiens GN=FN1 PE=1 SV=4	sp|P02751|FINC_HUMAN	263 kDa	182	19
Sorbin and SH3 domain-containing protein 1 OS=Mus musculus GN=Sorbs1 PE=1 SV=	sp|Q62417|SRBS1_MOUSE	143 kDa	47	0
Protein argonaute-2 OS=Mus musculus GN=Eif2c2 PE=1 SV=3	sp|Q8CJG0|AGO2_MOUSE (+1)	97 kDa	34	4
General vesicular transport factor p115 OS=Mus musculus GN=Uso1 PE=1 SV=2	sp|Q9Z1Z0|USO1_MOUSE	107 kDa	30	7
Calsequestrin-1 OS=Mus musculus GN=Casq1 PE=2 SV=3	sp|O09165|CASQ1_MOUSE	46 kDa	30	10
EH domain-binding protein 1-like protein 1 OS=Mus musculus GN=Ehbp1l1 PE=2 SV=:	sp|Q99MS7|EH1L1_MOUSE	185 kDa	28	8
Rho-associated protein kinase 2 OS=Mus musculus GN=Rock2 PE=1 SV=1	sp|P70336|ROCK2_MOUSE	161 kDa	46	0
Dystonin OS=Mus musculus GN=Dst PE=1 SV=1	sp|Q91ZU6|DYST_MOUSE	834 kDa	46	0
E3 ubiquitin-protein ligase NEDD4 OS=Mus musculus GN=Nedd4 PE=1 SV=3	sp|P46935|NEDD4_MOUSE	103 kDa	42	0
EH domain-containing protein 1 OS=Mus musculus GN=Ehd1 PE=1 SV=1	sp|Q9WVK4|EHD1_MOUSE	61 kDa	27	7
Serine/threonine-protein phosphatase 2A 55 kDa regulatory subunit B alpha isoform	sp|P63151|2ABA_HUMAN (+1)	52 kDa	30	2
Heat shock 70 kDa protein 1B OS=Mus musculus GN=Hspa1b PE=1 SV=3	sp|P17879|HS71B_MOUSE (+1)	70 kDa	41	0
Putative ATP-dependent RNA helicase DHX30 OS=Mus musculus GN=Dhx30 PE=2 SV=	sp|Q99PU8|DHX30_MOUSE	137 kDa	40	0
Phosphatidylinositide phosphatase SAC1 OS=Mus musculus GN=Sacm1l PE=2 SV=1	sp|Q9EP69|SAC1_MOUSE	67 kDa	39	0
Kinectin OS=Mus musculus GN=Ktn1 PE=1 SV=1	sp|Q61595|KTN1_MOUSE	153 kDa	28	3
Vigilin OS=Mus musculus GN=Hdlbp PE=1 SV=1	sp|Q8VDJ3|VIGLN_MOUSE	142 kDa	26	5

WD repeat-containing protein 11 OS=Mus musculus GN=Wdr11 PE=2 SV=1	sp|Q8K1X1|WDR11_MOUSE	136 kDa	39	0
Kinesin light chain 1 OS=Homo sapiens GN=KLC1 PE=1 SV=2	sp|Q07866|KLC1_HUMAN	65 kDa	21	5
60S ribosomal protein L17 OS=Mus musculus GN=Rpl17 PE=2 SV=3	sp|Q9CPR4|RL17_MOUSE	21 kDa	39	0
Pyrroline-5-carboxylate reductase 2 OS=Mus musculus GN=Pycr2 PE=2 SV=1	sp|Q922Q4|P5CR2_MOUSE	34 kDa	23	4
Lipoamide acyltransferase component of branched-chain alpha-keto acid dehydroger	sp|P53395|ODB2_MOUSE	53 kDa	25	3
Ras GTPase-activating-like protein IQGAP1 OS=Mus musculus GN=Iqgap1 PE=1 SV=1	sp|Q9JKF1|IQGA1_MOUSE	189 kDa	18	10
Myosin-14 OS=Mus musculus GN=Myh14 PE=1 SV=1	sp|Q6URW6|MYH14_MOUSE	229 kDa	38	0
1-phosphatidylinositol-4,5-bisphosphate phosphodiesterase gamma-1 OS=Mus musc	sp|Q62077|PLCG1_MOUSE	150 kDa	37	0
Basement membrane-specific heparan sulfate proteoglycan core protein OS=Homo s	sp|P98160|PGBM_HUMAN	469 kDa	37	0
Microtubule-actin cross-linking factor 1, isoforms 1/2/3/5 OS=Homo sapiens GN=MA(	sp|Q9UPN3|MACF1_HUMAN	620 kDa	37	0
MAP7 domain-containing protein 1 OS=Mus musculus GN=Map7d1 PE=1 SV=1	sp|A2AJI0|MA7D1_MOUSE	93 kDa	36	0
Fermitin family homolog 2 OS=Mus musculus GN=Fermt2 PE=1 SV=1	sp|Q8CIB5|FERM2_MOUSE	78 kDa	34	0
Golgin subfamily A member 3 OS=Mus musculus GN=Golga3 PE=1 SV=3	sp|P55937|GOGA3_MOUSE	167 kDa	23	2
CCR4-NOT transcription complex subunit 1 OS=Homo sapiens GN=CNOT1 PE=1 SV=2	sp|A5YKK6|CNOT1_HUMAN (+1)	267 kDa	33	0
Utrophin OS=Homo sapiens GN=UTRN PE=1 SV=2	sp|P46939|UTRO_HUMAN	394 kDa	33	0
Tripeptidyl-peptidase 2 OS=Mus musculus GN=Tpp2 PE=2 SV=3	sp|Q64514|TPP2_MOUSE	140 kDa	32	0
BAG family molecular chaperone regulator 3 OS=Mus musculus GN=Bag3 PE=1 SV=2	sp|Q9JLV1|BAG3_MOUSE	62 kDa	24	2
EH domain-containing protein 4 OS=Mus musculus GN=Ehd4 PE=1 SV=1	sp|Q9EQP2|EHD4_MOUSE	61 kDa	23	9
Nischarin OS=Mus musculus GN=Nisch PE=1 SV=2	sp|Q80TM9|NISCH_MOUSE	175 kDa	31	0
Active breakpoint cluster region-related protein OS=Mus musculus GN=Abr PE=2 SV=	sp|Q5SSL4|ABR_MOUSE	98 kDa	30	0
Heat shock-related 70 kDa protein 2 OS=Homo sapiens GN=HSPA2 PE=1 SV=1	sp|P54652|HSP72_HUMAN	70 kDa	30	0
ATP-binding cassette sub-family F member 2 OS=Mus musculus GN=Abcf2 PE=2 SV=1	sp|Q99LE6|ABCF2_MOUSE	72 kDa	29	0
Nck-associated protein 1 OS=Mus musculus GN=Nckap1 PE=2 SV=2	sp|P28660|NCKP1_MOUSE	129 kDa	28	0
Insulin-like growth factor-binding protein 5 OS=Mus musculus GN=Igfbp5 PE=1 SV=1	sp|Q07079|IBP5_MOUSE	30 kDa	18	3
Fibulin-2 OS=Mus musculus GN=Fbln2 PE=1 SV=1	sp|P37889|FBLN2_MOUSE	132 kDa	27	0
FACT complex subunit SPT16 OS=Homo sapiens GN=SUPT16H PE=1 SV=1	sp|Q9Y5B9|SP16H_HUMAN	120 kDa	26	0
Rho guanine nucleotide exchange factor 7 OS=Mus musculus GN=Arhgef7 PE=1 SV=2	sp|Q9ES28|ARHG7_MOUSE	97 kDa	26	0
60S ribosomal protein L15 OS=Homo sapiens GN=RPL15 PE=1 SV=2	sp|P61313|RL15_HUMAN (+1)	24 kDa	26	0
60S ribosomal protein L21 OS=Mus musculus GN=Rpl21 PE=2 SV=3	sp|O09167|RL21_MOUSE	19 kDa	26	0
Intersectin-2 OS=Mus musculus GN=Itsn2 PE=1 SV=2	sp|Q9Z0R6|ITSN2_MOUSE	189 kDa	25	0
E3 ubiquitin-protein ligase HUWE1 OS=Homo sapiens GN=HUWE1 PE=1 SV=3	sp|Q7Z6Z7|HUWE1_HUMAN	482 kDa	25	0
DnaJ homolog subfamily C member 7 OS=Mus musculus GN=Dnajc7 PE=1 SV=2	sp|Q9QYI3|DNJC7_MOUSE	56 kDa	18	3
Ubiquitin-associated and SH3 domain-containing protein B OS=Mus musculus GN=Ub	sp|Q8BGG7|UBS3B_MOUSE	71 kDa	25	0
Hornerin OS=Homo sapiens GN=HRNR PE=1 SV=2	sp|Q86YZ3|HORN_HUMAN	282 kDa	16	2
Transcriptional activator protein Pur-alpha OS=Mus musculus GN=Pura PE=1 SV=1	sp|P42669|PURA_MOUSE (+1)	35 kDa	25	5
Transforming growth factor-beta-induced protein ig-h3 OS=Homo sapiens GN=TGFBI	sp|Q15582|BGH3_HUMAN	75 kDa	24	0
Squamous cell carcinoma antigen recognized by T-cells 3 OS=Mus musculus GN=Sart3	sp|Q9JLI8|SART3_MOUSE	110 kDa	17	2
Mitogen-activated protein kinase 1 OS=Mus musculus GN=Mapk1 PE=1 SV=3	sp|P63085|MK01_MOUSE	41 kDa	16	4
Cytoplasmic FMR1-interacting protein 2 OS=Mus musculus GN=Cyfip2 PE=1 SV=2	sp|Q5SQX6|CYFP2_MOUSE	146 kDa	24	0
RNA-binding protein Musashi homolog 2 OS=Mus musculus GN=Msi2 PE=1 SV=1	sp|Q920Q6|MSI2H_MOUSE	37 kDa	17	4
60S ribosomal protein L19 OS=Homo sapiens GN=RPL19 PE=1 SV=1	sp|P84098|RL19_HUMAN (+1)	23 kDa	24	0
Lupus La protein homolog OS=Mus musculus GN=Ssb PE=2 SV=1	sp|P32067|LA_MOUSE	48 kDa	23	0
Beta-taxilin OS=Mus musculus GN=Txlnb PE=2 SV=2	sp|Q8VBT1|TXLNB_MOUSE	77 kDa	23	0
Ubiquitin carboxyl-terminal hydrolase 7 OS=Mus musculus GN=Usp7 PE=1 SV=1	sp|Q6A4J8|UBP7_MOUSE	128 kDa	23	0
Nucleolin OS=Homo sapiens GN=NCL PE=1 SV=3	sp|P19338|NUCL_HUMAN	77 kDa	23	0
C-terminal-binding protein 1 OS=Mus musculus GN=Ctbp1 PE=1 SV=2	sp|O88712|CTBP1_MOUSE	48 kDa	14	3
Filamin-B OS=Mus musculus GN=Flnb PE=1 SV=3	sp|Q80X90|FLNB_MOUSE	278 kDa	60	21
Dynactin subunit 1 OS=Mus musculus GN=Dctn1 PE=1 SV=3	sp|O08788|DCTN1_MOUSE	142 kDa	22	0
Constitutive coactivator of PPAR-gamma-like protein 1 OS=Mus musculus GN=FAM12	sp|Q6A0A9|F120A_MOUSE	122 kDa	22	0
Phospholipid hydroperoxide glutathione peroxidase, mitochondrial OS=Mus musculu	sp|O70325|GPX41_MOUSE	22 kDa	22	0
Rab3 GTPase-activating protein non-catalytic subunit OS=Mus musculus GN=Rab3gap	sp|Q8BMG7|RBGPR_MOUSE	153 kDa	14	2
Nucleophosmin OS=Mus musculus GN=Npm1 PE=1 SV=1	sp|Q61937|NPM_MOUSE	33 kDa	12	3
1-phosphatidylinositol-4,5-bisphosphate phosphodiesterase gamma-2 OS=Mus musc	sp|Q8CIH5|PLCG2_MOUSE	148 kDa	22	0
60S ribosomal protein L18 OS=Mus musculus GN=Rpl18 PE=2 SV=3	sp|P35980|RL18_MOUSE	22 kDa	22	0
40S ribosomal protein S24 OS=Homo sapiens GN=RPS24 PE=1 SV=1	sp|P62847|RS24_HUMAN (+1)	15 kDa	22	0
Liprin-beta-1 OS=Mus musculus GN=Ppfibp1 PE=1 SV=3	sp|Q8C8U0|LIPB1_MOUSE	109 kDa	21	0
Vacuolar protein sorting-associated protein 13A OS=Mus musculus GN=Vps13a PE=1	sp|Q5H8C4|VP13A_MOUSE	359 kDa	21	0
Kinesin light chain 1 OS=Mus musculus GN=Klc1 PE=1 SV=3	sp|O88447|KLC1_MOUSE	61 kDa	21	0
Neural cell adhesion molecule 1 OS=Mus musculus GN=Ncam1 PE=1 SV=3	sp|P13595|NCAM1_MOUSE	119 kDa	12	4
Protein prune homolog 2 OS=Mus musculus GN=Prune2 PE=2 SV=2	sp|Q52KR3|PRUN2_MOUSE	340 kDa	21	0
Lon protease homolog, mitochondrial OS=Mus musculus GN=Lonp1 PE=1 SV=2	sp|Q8CGK3|LONM_MOUSE	106 kDa	20	0
Elongator complex protein 1 OS=Mus musculus GN=Ikbkap PE=2 SV=2	sp|Q7TT37|ELP1_MOUSE	150 kDa	20	0
Prolow-density lipoprotein receptor-related protein 1 OS=Mus musculus GN=Lrp1 PE	sp|Q91ZX7|LRP1_MOUSE	505 kDa	20	0
Thrombospondin-1 OS=Mus musculus GN=Thbs1 PE=1 SV=1	sp|P35441|TSP1_MOUSE	130 kDa	20	0
Protein arginine N-methyltransferase 5 OS=Mus musculus GN=Prmt5 PE=1 SV=3	sp|Q8CIG8|ANM5_MOUSE	73 kDa	12	3
ARF GTPase-activating protein GIT1 OS=Homo sapiens GN=GIT1 PE=1 SV=2	sp|Q9Y2X7|GIT1_HUMAN	84 kDa	20	0
Cytoplasmic FMR1-interacting protein 1 OS=Homo sapiens GN=CYFIP1 PE=1 SV=1	sp|Q7L576|CYFP1_HUMAN (+1)	145 kDa	20	0
Disco-interacting protein 2 homolog C OS=Homo sapiens GN=DIP2C PE=2 SV=2	sp|Q9Y2E4|DIP2C_HUMAN	171 kDa	19	0
Activating signal cointegrator 1 complex subunit 3 OS=Homo sapiens GN=ASCC3 PE=1	sp|Q8N3C0|HELC1_HUMAN	251 kDa	19	0
Tankyrase-1 OS=Mus musculus GN=Tnks PE=2 SV=1	sp|Q6PFX9|TNKS1_MOUSE	141 kDa	19	0
Procollagen-lysine,2-oxoglutarate 5-dioxygenase 1 OS=Mus musculus GN=Plod1 PE=1	sp|Q9R0E2|PLOD1_MOUSE	84 kDa	11	3
Insulin-like growth factor 2 mRNA-binding protein 2 OS=Mus musculus GN=Igf2bp2 P	sp|Q5SF07|IF2B2_MOUSE	66 kDa	12	2
Destrin OS=Mus musculus GN=Dstn PE=1 SV=3	sp|Q9R0P5|DEST_MOUSE	19 kDa	10	3
Phosphorylase b kinase regulatory subunit beta OS=Mus musculus GN=Phkb PE=1 SV	sp|Q7TSH2|KPBB_MOUSE	124 kDa	10	2
UPF0424 protein C1orf128 OS=Homo sapiens GN=C1orf128 PE=1 SV=1	sp|Q9GZP4|CA128_HUMAN	24 kDa	12	4
Fatty acid synthase OS=Homo sapiens GN=FASN PE=1 SV=3	sp|P49327|FAS_HUMAN	273 kDa	19	0
RNA-binding protein FUS OS=Homo sapiens GN=FUS PE=1 SV=1	sp|P35637|FUS_HUMAN (+1)	53 kDa	19	0

Dual specificity tyrosine-phosphorylation-regulated kinase 1A OS=Homo sapiens GN=	sp|Q13627|DYR1A_HUMAN (+1)	86 kDa	18	0
Microtubule-associated protein 1A OS=Mus musculus GN=Map1a PE=1 SV=2	sp|Q9QYR6|MAP1A_MOUSE	300 kDa	18	0
Eukaryotic translation initiation factor 3 subunit A OS=Mus musculus GN=Eif3a PE=1 S	sp|P23116|EIF3A_MOUSE (+1)	162 kDa	18	0
AP-1 complex subunit mu-1 OS=Mus musculus GN=Ap1m1 PE=1 SV=3	sp|P35585|AP1M1_MOUSE	49 kDa	11	2
Vacuolar protein sorting-associated protein 26A OS=Homo sapiens GN=VPS26A PE=1	sp|O75436|VP26A_HUMAN (+1)	38 kDa	17	0
Nuclear export mediator factor Nemf OS=Mus musculus GN=Nemf PE=1 SV=2	sp|Q8CCP0|NEMF_MOUSE	121 kDa	17	0
DDB1- and CUL4-associated factor 7 OS=Homo sapiens GN=DCAF7 PE=1 SV=1	sp|P61962|DCAF7_HUMAN (+1)	39 kDa	17	0
Myotubularin-related protein 5 OS=Mus musculus GN=Sbf1 PE=1 SV=2	sp|Q6ZPE2|MTMR5_MOUSE	209 kDa	17	0
Ribosome-binding protein 1 OS=Mus musculus GN=Rrbp1 PE=2 SV=2	sp|Q99PL5|RRBP1_MOUSE	173 kDa	17	0
Coatomer subunit epsilon OS=Mus musculus GN=Cope PE=2 SV=3	sp|O89079|COPE_MOUSE	35 kDa	16	0
ELAV-like protein 1 OS=Mus musculus GN=Elavl1 PE=1 SV=2	sp|P70372|ELAV1_MOUSE	36 kDa	9	3
Leucyl-tRNA synthetase, cytoplasmic OS=Homo sapiens GN=LARS PE=1 SV=2	sp|Q9P2J5|SYLC_HUMAN	134 kDa	16	0
Myomesin-2 OS=Homo sapiens GN=MYOM2 PE=2 SV=2	sp|P54296|MYOM2_HUMAN	165 kDa	15	0
Programmed cell death 6-interacting protein OS=Mus musculus GN=Pdcd6ip PE=1 SV	sp|Q9WU78|PDC6I_MOUSE	96 kDa	15	0
Ubiquitin carboxyl-terminal hydrolase 4 OS=Mus musculus GN=Usp4 PE=1 SV=3	sp|P35123|UBP4_MOUSE	108 kDa	15	0
Ankyrin repeat and FYVE domain-containing protein 1 OS=Mus musculus GN=Ankfy1	sp|Q810B6|ANFY1_MOUSE	129 kDa	15	0
Calponin-2 OS=Mus musculus GN=Cnn2 PE=2 SV=1	sp|Q08093|CNN2_MOUSE	33 kDa	15	0
Fibronectin type III domain-containing protein 3B OS=Mus musculus GN=Fndc3b PE=	sp|Q6NWW9|FND3B_MOUSE	133 kDa	15	0
Midline-1 OS=Mus musculus GN=Mid1 PE=1 SV=2	sp|O70583|TRI18_MOUSE	76 kDa	15	0
Serine/threonine-protein phosphatase 2A catalytic subunit alpha isoform OS=Mus m	sp|P63330|PP2AA_MOUSE (+1)	36 kDa	15	0
Nestin OS=Homo sapiens GN=NES PE=1 SV=2	sp|P48681|NEST_HUMAN	177 kDa	15	0
60S ribosomal protein L27a OS=Mus musculus GN=Rpl27a PE=2 SV=5	sp|P14115|RL27A_MOUSE	17 kDa	15	0
Serine/arginine-rich splicing factor 3 OS=Homo sapiens GN=SRSF3 PE=1 SV=1	sp|P84103|SRSF3_HUMAN (+1)	19 kDa	15	0
Phosphatidylinositol-3,4,5-trisphosphate 5-phosphatase 2 OS=Homo sapiens GN=INP	sp|O15357|SHIP2_HUMAN (+1)	139 kDa	14	0
Protein transport protein Sec24C OS=Homo sapiens GN=SEC24C PE=1 SV=3	sp|P53992|SC24C_HUMAN	118 kDa	14	0
Growth factor receptor-bound protein 2 OS=Homo sapiens GN=GRB2 PE=1 SV=1	sp|P62993|GRB2_HUMAN (+1)	25 kDa	14	0
Regulatory-associated protein of mTOR OS=Homo sapiens GN=RPTOR PE=1 SV=1	sp|Q8N122|RPTOR_HUMAN	149 kDa	14	0
Syntenin-1 OS=Mus musculus GN=Sdcbp PE=2 SV=1	sp|O08992|SDCB1_MOUSE	32 kDa	14	0
Vacuolar protein sorting-associated protein 13C OS=Mus musculus GN=Vps13c PE=1 S	sp|Q8BX70|VP13C_MOUSE	420 kDa	14	0
Fragile X mental retardation syndrome-related protein 2 OS=Homo sapiens GN=FXR2	sp|P51116|FXR2_HUMAN	74 kDa	14	0
Structural maintenance of chromosomes protein 1A OS=Mus musculus GN=Smc1a PE	sp|Q9CU62|SMC1A_MOUSE	143 kDa	14	0
60S ribosomal protein L32 OS=Homo sapiens GN=RPL32 PE=1 SV=2	sp|P62910|RL32_HUMAN (+1)	16 kDa	14	0
Structural maintenance of chromosomes protein 3 OS=Mus musculus GN=Smc3 PE=1	sp|Q9CW03|SMC3_MOUSE (+1)	142 kDa	14	0
Myeloid-associated differentiation marker OS=Mus musculus GN=Myadm PE=2 SV=2	sp|O35682|MYADM_MOUSE	35 kDa	13	0
Nidogen-2 OS=Mus musculus GN=Nid2 PE=1 SV=2	sp|O88322|NID2_MOUSE	154 kDa	13	0
Calcium-binding and coiled-coil domain-containing protein 1 OS=Mus musculus GN=(	sp|Q8CGU1|CACO1_MOUSE	77 kDa	13	0
Microtubule-actin cross-linking factor 1, isoform 4 OS=Homo sapiens GN=MACF1 PE=	sp|Q96PK2|MACF4_HUMAN	670 kDa	13	0
Serine/arginine-rich splicing factor 1 OS=Homo sapiens GN=SRSF1 PE=1 SV=2	sp|Q07955|SRSF1_HUMAN (+1)	28 kDa	13	0
Vimentin OS=Homo sapiens GN=VIM PE=1 SV=4	sp|P08670|VIME_HUMAN	54 kDa	256	111
Glypican-1 OS=Mus musculus GN=Gpc1 PE=1 SV=1	sp|Q9QZF2|GPC1_MOUSE	61 kDa	6	2
Serine/arginine-rich splicing factor 6 OS=Homo sapiens GN=SRSF6 PE=1 SV=2	sp|Q13247|SRSF6_HUMAN	40 kDa	13	0
FYVE and coiled-coil domain-containing protein 1 OS=Mus musculus GN=Fyco1 PE=1	sp|Q8VDC1|FYCO1_MOUSE	162 kDa	12	0
Protein transport protein Sec23B OS=Mus musculus GN=Sec23b PE=2 SV=1	sp|Q9D662|SC23B_MOUSE	86 kDa	10	5
Synaptopodin 2-like protein OS=Mus musculus GN=Synpo2l PE=2 SV=1	sp|Q8BWB1|SYP2L_MOUSE	103 kDa	12	0
Mitogen-activated protein kinase kinase kinase kinase 4 OS=Mus musculus GN=Map4	sp|P97820|M4K4_MOUSE	141 kDa	12	0
Tight junction protein ZO-1 OS=Mus musculus GN=Tjp1 PE=1 SV=2	sp|P39447|ZO1_MOUSE	195 kDa	12	0
Interleukin enhancer-binding factor 3 OS=Homo sapiens GN=ILF3 PE=1 SV=3	sp|Q12906|ILF3_HUMAN	95 kDa	12	0
C-Jun-amino-terminal kinase-interacting protein 3 OS=Mus musculus GN=Mapk8ip3 F	sp|Q9ESN9|JIP3_MOUSE	148 kDa	12	0
Arginine and glutamate-rich protein 1 OS=Mus musculus GN=Arglu1 PE=1 SV=2	sp|Q3UL36|ARGL1_MOUSE (+1)	33 kDa	12	0
Kinesin-like protein KIF2A OS=Homo sapiens GN=KIF2A PE=1 SV=3	sp|O00139|KIF2A_HUMAN (+1)	80 kDa	12	0
Eukaryotic translation initiation factor 5B OS=Mus musculus GN=Eif5b PE=1 SV=2	sp|Q05D44|IF2P_MOUSE	138 kDa	12	0
DNA-directed RNA polymerase II subunit RPB2 OS=Homo sapiens GN=POLR2B PE=1 S	sp|P30876|RPB2_HUMAN (+1)	134 kDa	12	0
Heterogeneous nuclear ribonucleoprotein R OS=Homo sapiens GN=HNRNPR PE=1 SV	sp|O43390|HNRPR_HUMAN	71 kDa	12	0
Mimecan OS=Homo sapiens GN=OGN PE=1 SV=1	sp|P20774|MIME_HUMAN	34 kDa	11	0
La-related protein 1 OS=Mus musculus GN=Larp1 PE=1 SV=2	sp|Q6ZQ58|LARP1_MOUSE	121 kDa	11	0
NEDD9-interacting protein with calponin homology and LIM domains OS=Mus muscu	sp|Q8VDP3|MICA1_MOUSE	117 kDa	11	0
Protein KIAA0664 OS=Mus musculus GN=Kiaa0664 PE=2 SV=2	sp|Q5SW19|K0664_MOUSE	148 kDa	7	2
Pleckstrin homology-like domain family A member 3 OS=Mus musculus GN=Phlda3 P	sp|Q9WV95|PHLA3_MOUSE	14 kDa	6	2
Uncharacterized protein KIAA0564 homolog OS=Mus musculus GN=Kiaa0564 PE=2 S\	sp|Q8CC88|K0564_MOUSE	213 kDa	11	0
Ataxin-2 OS=Mus musculus GN=Atxn2 PE=1 SV=1	sp|O70305|ATX2_MOUSE	136 kDa	11	0
Coiled-coil domain-containing protein 165 OS=Mus musculus GN=Ccdc165 PE=1 SV=1	sp|Q3UHU5|CC165_MOUSE	214 kDa	11	0
Myelin expression factor 2 OS=Mus musculus GN=Myef2 PE=1 SV=1	sp|Q8C854|MYEF2_MOUSE	63 kDa	11	0
NEDD8 ultimate buster 1 OS=Mus musculus GN=Nub1 PE=1 SV=2	sp|P54729|NUB1_MOUSE	70 kDa	11	0
Guanine nucleotide-binding protein G(I)/G(S)/G(T) subunit beta-1 OS=Homo sapiens (	sp|P62873|GBB1_HUMAN (+1)	37 kDa	11	0
Structural maintenance of chromosomes protein 2 OS=Homo sapiens GN=SMC2 PE=1	sp|O95347|SMC2_HUMAN	136 kDa	11	0
28S ribosomal protein S22, mitochondrial OS=Mus musculus GN=Mrps22 PE=2 SV=1	sp|Q9CXW2|RT22_MOUSE	41 kDa	11	0
Phosphatidylinositol-4-phosphate 3-kinase C2 domain-containing subunit alpha OS=H	sp|O00443|P3C2A_HUMAN (+1)	191 kDa	10	0
Dystrophin OS=Mus musculus GN=Dmd PE=1 SV=3	sp|P11531|DMD_MOUSE	426 kDa	10	0
LIM and senescent cell antigen-like-containing domain protein 1 OS=Homo sapiens G	sp|P48059|LIMS1_HUMAN (+1)	37 kDa	10	0
Casein kinase II subunit alpha OS=Homo sapiens GN=CSNK2A1 PE=1 SV=1	sp|P68400|CSK21_HUMAN (+1)	45 kDa	10	0
Calcium uptake protein 1, mitochondrial OS=Mus musculus GN=Cbara1 PE=2 SV=1	sp|Q8VCX5|MICU1_MOUSE	54 kDa	10	0
GTP-binding protein 1 OS=Homo sapiens GN=GTPBP1 PE=1 SV=3	sp|O00178|GTPB1_HUMAN (+1)	72 kDa	10	0
STE20-like serine/threonine-protein kinase OS=Mus musculus GN=Slk PE=1 SV=2	sp|O54988|SLK_MOUSE	141 kDa	10	0
Tripartite motif-containing protein 47 OS=Mus musculus GN=Trim47 PE=2 SV=2	sp|Q8C0E3|TRI47_MOUSE	70 kDa	10	0
Carbohydrate kinase domain-containing protein OS=Mus musculus GN=Carkd PE=1 S	sp|Q9CZ42|CARKD_MOUSE	37 kDa	10	0
Junctophilin-2 OS=Mus musculus GN=Jph2 PE=1 SV=2	sp|Q9ET78|JPH2_MOUSE	75 kDa	5	2
Four and a half LIM domains protein 3 OS=Mus musculus GN=Fhl3 PE=2 SV=1	sp|Q9R059|FHL3_MOUSE	32 kDa	5	2

Fragile X mental retardation syndrome-related protein 2 OS=Mus musculus GN=Fxr2	sp|Q9WVR4|FXR2_MOUSE	74 kDa	10	0
Adenylosuccinate lyase OS=Mus musculus GN=Adsl PE=2 SV=2	sp|P54822|PUR8_MOUSE	55 kDa	10	0
60S ribosomal protein L36a OS=Homo sapiens GN=RPL36A PE=1 SV=2	sp|P83881|RL36A_HUMAN (+2)	12 kDa	10	0
Proliferating cell nuclear antigen OS=Mus musculus GN=Pcna PE=1 SV=2	sp|P17918|PCNA_MOUSE	29 kDa	10	0
28S ribosomal protein S9, mitochondrial OS=Mus musculus GN=Mrps9 PE=1 SV=3	sp|Q9D7N3|RT09_MOUSE	45 kDa	10	0
60S ribosomal protein L36 OS=Homo sapiens GN=RPL36 PE=1 SV=3	sp|Q9Y3U8|RL36_HUMAN	12 kDa	10	0
Heterogeneous nuclear ribonucleoprotein A0 OS=Homo sapiens GN=HNRNPA0 PE=1	sp|Q13151|ROA0_HUMAN	31 kDa	10	0
Nebulin-related-anchoring protein OS=Mus musculus GN=Nrap PE=1 SV=3	sp|Q80XB4|NRAP_MOUSE	196 kDa	9	0
Atlastin-2 OS=Mus musculus GN=Atl2 PE=1 SV=1	sp|Q6PA06|ATLA2_MOUSE	66 kDa	9	0
Trinucleotide repeat-containing gene 6B protein OS=Mus musculus GN=Tnrc6b PE=2	sp|Q8BKI2|TNR6B_MOUSE	192 kDa	9	0
Dedicator of cytokinesis protein 11 OS=Mus musculus GN=Dock11 PE=1 SV=1	sp|A2AF47|DOC11_MOUSE	238 kDa	9	0
Protein tyrosine phosphatase type IVA 2 OS=Mus musculus GN=Ptp4a2 PE=1 SV=1	sp|O70274|TP4A2_MOUSE (+1)	19 kDa	9	0
Lys-63-specific deubiquitinase BRCC36 OS=Mus musculus GN=Brcc3 PE=2 SV=1	sp|P46737|BRCC3_MOUSE	33 kDa	9	0
Core-binding factor subunit beta OS=Homo sapiens GN=CBFB PE=1 SV=2	sp|Q13951|PEBB_HUMAN	22 kDa	9	0
26S proteasome non-ATPase regulatory subunit 4 OS=Mus musculus GN=Psmd4 PE=1	sp|O35226|PSMD4_MOUSE (+1)	41 kDa	9	0
Serine/threonine-protein kinase MRCK beta OS=Mus musculus GN=Cdc42bpb PE=1 S	sp|Q7TT50|MRCKB_MOUSE	195 kDa	9	0
Poly(U)-binding-splicing factor PUF60 OS=Mus musculus GN=Puf60 PE=2 SV=2	sp|Q3UEB3|PUF60_MOUSE (+1)	60 kDa	9	0
Transcription elongation factor, mitochondrial OS=Mus musculus GN=Tefm PE=2 SV=	sp|Q5SSK3|TEFM_MOUSE	42 kDa	9	0
C-1-tetrahydrofolate synthase, cytoplasmic OS=Mus musculus GN=Mthfd1 PE=1 SV=4	sp|Q922D8|C1TC_MOUSE	101 kDa	9	0
Heat shock protein beta-7 OS=Homo sapiens GN=HSPB7 PE=1 SV=1	sp|Q9UBY9|HSPB7_HUMAN	19 kDa	9	0
Acetylcholine receptor subunit alpha OS=Mus musculus GN=Chrna1 PE=1 SV=1	sp|P04756|ACHA_MOUSE	52 kDa	8	0
Lysosomal protective protein OS=Mus musculus GN=Ctsa PE=1 SV=1	sp|P16675|PPGB_MOUSE	54 kDa	8	0
Protein kinase C and casein kinase II substrate protein 3 OS=Mus musculus GN=Pacsi	sp|Q99JB8|PACN3_MOUSE	49 kDa	8	0
Integrin-linked protein kinase OS=Mus musculus GN=Ilk PE=1 SV=2	sp|O55222|ILK_MOUSE	51 kDa	8	0
Junctophilin-1 OS=Mus musculus GN=Jph1 PE=1 SV=1	sp|Q9ET80|JPH1_MOUSE	72 kDa	8	0
Signal recognition particle 68 kDa protein OS=Mus musculus GN=Srp68 PE=2 SV=2	sp|Q8BMA6|SRP68_MOUSE	71 kDa	8	0
UPF0568 protein C14orf166 homolog OS=Mus musculus PE=2 SV=1	sp|Q9CQE8|CN166_MOUSE (+1)	28 kDa	8	0
Intersectin-1 OS=Mus musculus GN=Itsn1 PE=1 SV=1	sp|Q9Z0R4|ITSN1_MOUSE	194 kDa	8	0
Moesin OS=Homo sapiens GN=MSN PE=1 SV=3	sp|P26038|MOES_HUMAN	68 kDa	129	69
Serine/threonine-protein phosphatase 2A 55 kDa regulatory subunit B delta isoform	sp|Q66LE6|2ABD_HUMAN (+1)	52 kDa	8	0
ATP-dependent RNA helicase Dhx29 OS=Mus musculus GN=Dhx29 PE=2 SV=1	sp|Q6PGC1|DHX29_MOUSE	154 kDa	8	0
Phenylalanyl-tRNA synthetase alpha chain OS=Mus musculus GN=Farsa PE=2 SV=1	sp|Q8C0C7|SYFA_MOUSE	58 kDa	8	0
Nucleolar RNA helicase 2 OS=Homo sapiens GN=DDX21 PE=1 SV=5	sp|Q9NR30|DDX21_HUMAN	87 kDa	8	0
L-lactate dehydrogenase A chain OS=Homo sapiens GN=LDHA PE=1 SV=2	sp|P00338|LDHA_HUMAN	37 kDa	8	0
Protein bicaudal D homolog 2 OS=Mus musculus GN=Bicd2 PE=1 SV=1	sp|Q921C5|BICD2_MOUSE	93 kDa	7	0
Golgin subfamily A member 4 OS=Mus musculus GN=Golga4 PE=1 SV=2	sp|Q91VW5|GOGA4_MOUSE	258 kDa	7	0
Vitronectin OS=Homo sapiens GN=VTN PE=1 SV=1	sp|P04004|VTNC_HUMAN	54 kDa	7	0
WD repeat-containing protein 47 OS=Mus musculus GN=Wdr47 PE=1 SV=2	sp|Q8CGF6|WDR47_MOUSE	102 kDa	7	0
WD repeat-containing protein 6 OS=Mus musculus GN=Wdr6 PE=2 SV=1	sp|Q99ME2|WDR6_MOUSE	122 kDa	7	0
AP-2 complex subunit alpha-2 OS=Mus musculus GN=Ap2a2 PE=1 SV=2	sp|P17427|AP2A2_MOUSE	104 kDa	7	0
Rho GTPase-activating protein 10 OS=Mus musculus GN=Arhgap10 PE=1 SV=2	sp|Q6Y5D8|RHG10_MOUSE	89 kDa	7	0
ATP-dependent Clp protease ATP-binding subunit clpX-like, mitochondrial OS=Homo	sp|O76031|CLPX_HUMAN (+1)	69 kDa	7	0
Prostaglandin E synthase 3 OS=Homo sapiens GN=PTGES3 PE=1 SV=1	sp|Q15185|TEBP_HUMAN (+1)	19 kDa	7	0
182 kDa tankyrase-1-binding protein OS=Mus musculus GN=Tnks1bp1 PE=1 SV=2	sp|P58871|TB182_MOUSE	182 kDa	7	0
Dual specificity protein phosphatase 3 OS=Mus musculus GN=Dusp3 PE=1 SV=1	sp|Q9D7X3|DUS3_MOUSE	20 kDa	7	0
Tyrosine-protein kinase ABL2 OS=Homo sapiens GN=ABL2 PE=1 SV=1	sp|P42684|ABL2_HUMAN	128 kDa	7	0
Serum deprivation-response protein OS=Mus musculus GN=Sdpr PE=1 SV=3	sp|Q63918|SDPR_MOUSE	47 kDa	7	0
Nidogen-1 OS=Mus musculus GN=Nid1 PE=1 SV=2	sp|P10493|NID1_MOUSE	137 kDa	7	0
Tumor susceptibility gene 101 protein OS=Mus musculus GN=Tsg101 PE=1 SV=2	sp|Q61187|TS101_MOUSE (+1)	44 kDa	7	0
COMM domain-containing protein 3 OS=Mus musculus GN=Commd3 PE=2 SV=1	sp|Q63829|COMD3_MOUSE	22 kDa	7	0
WD repeat-containing protein 26 OS=Mus musculus GN=Wdr26 PE=1 SV=3	sp|Q8C6G8|WDR26_MOUSE (+1)	71 kDa	7	0
Beta-1-syntrophin OS=Mus musculus GN=Sntb1 PE=1 SV=4	sp|Q99L88|SNTB1_MOUSE	58 kDa	7	0
Girdin OS=Mus musculus GN=Ccdc88a PE=1 SV=2	sp|Q5SNZ0|GRDN_MOUSE	216 kDa	7	0
Constitutive coactivator of PPAR-gamma-like protein 2 OS=Mus musculus GN=Fam12	sp|Q8C3F2|F120C_MOUSE	120 kDa	7	0
Serine/threonine-protein phosphatase PP1-alpha catalytic subunit OS=Homo sapiens	sp|P62136|PP1A_HUMAN (+1)	38 kDa	7	0
CTP synthase 1 OS=Mus musculus GN=Ctps PE=1 SV=2	sp|P70698|PYRG1_MOUSE	67 kDa	7	0
DNA ligase 3 OS=Mus musculus GN=Lig3 PE=1 SV=2	sp|P97386|DNLI3_MOUSE	113 kDa	7	0
5'-3' exoribonuclease 1 OS=Mus musculus GN=Xrn1 PE=1 SV=1	sp|P97789|XRN1_MOUSE	194 kDa	7	0
Ubiquitin carboxyl-terminal hydrolase 40 OS=Mus musculus GN=Usp40 PE=1 SV=2	sp|Q8BWR4|UBP40_MOUSE	140 kDa	7	0
TATA-binding protein-associated factor 2N OS=Homo sapiens GN=TAF15 PE=1 SV=1	sp|Q92804|RBP56_HUMAN	62 kDa	7	0
Nucleolar transcription factor 1 OS=Homo sapiens GN=UBTF PE=1 SV=1	sp|P17480|UBF1_HUMAN (+1)	89 kDa	7	0
U1 small nuclear ribonucleoprotein A OS=Mus musculus GN=Snrpa PE=2 SV=3	sp|Q62189|SNRPA_MOUSE	32 kDa	7	0
39S ribosomal protein L48, mitochondrial OS=Mus musculus GN=Mrpl48 PE=2 SV=1	sp|Q8JZS9|RM48_MOUSE	24 kDa	7	0
Pyrroline-5-carboxylate reductase 3 OS=Mus musculus GN=Pycrl PE=2 SV=2	sp|Q9DCC4|P5CR3_MOUSE	29 kDa	7	0
28S ribosomal protein S29, mitochondrial OS=Mus musculus GN=Dap3 PE=2 SV=1	sp|Q9ER88|RT29_MOUSE	45 kDa	7	0
[3-methyl-2-oxobutanoate dehydrogenase [lipoamide]] kinase, mitochondrial OS=Mu	sp|O55028|BCKD_MOUSE	47 kDa	7	0
DnaJ homolog subfamily B member 6 OS=Mus musculus GN=Dnajb6 PE=1 SV=4	sp|O54946|DNJB6_MOUSE	40 kDa	6	0
Cleavage and polyadenylation specificity factor subunit 5 OS=Homo sapiens GN=NUD	sp|O43809|CPSF5_HUMAN (+1)	26 kDa	6	0
Fat storage-inducing transmembrane protein 2 OS=Mus musculus GN=Fitm2 PE=1 SV	sp|P59266|FITM2_MOUSE	30 kDa	6	0
Myomesin-1 OS=Mus musculus GN=Myom1 PE=1 SV=2	sp|Q62234|MYOM1_MOUSE	185 kDa	6	0
Lysocardiolipin acyltransferase 1 OS=Mus musculus GN=Lclat1 PE=2 SV=2	sp|Q3UN02|LCLT1_MOUSE	44 kDa	6	0
Protein transport protein Sec16A OS=Homo sapiens GN=SEC16A PE=1 SV=3	sp|O15027|SC16A_HUMAN	234 kDa	6	0
Guanine nucleotide exchange factor VAV2 OS=Mus musculus GN=Vav2 PE=1 SV=1	sp|Q60992|VAV2_MOUSE	100 kDa	6	0
Myocyte-specific enhancer factor 2A OS=Homo sapiens GN=MEF2A PE=1 SV=1	sp|Q02078|MEF2A_HUMAN	55 kDa	6	0
Serine protease HTRA1 OS=Mus musculus GN=Htra1 PE=2 SV=2	sp|Q9R118|HTRA1_MOUSE	51 kDa	6	0
Casein kinase I isoform epsilon OS=Homo sapiens GN=CSNK1E PE=1 SV=1	sp|P49674|KC1E_HUMAN (+1)	47 kDa	6	0
Disco-interacting protein 2 homolog B OS=Mus musculus GN=Dip2b PE=1 SV=1	sp|Q3UH60|DIP2B_MOUSE (+1)	171 kDa	6	0

Coatomer subunit zeta-1 OS=Homo sapiens GN=COPZ1 PE=1 SV=1	sp|P61923|COPZ1_HUMAN (+1)	20 kDa	6	0
Golgi resident protein GCP60 OS=Mus musculus GN=Acbd3 PE=1 SV=3	sp|Q8BMP6|GCP60_MOUSE	60 kDa	6	0
Protein diaphanous homolog 1 OS=Mus musculus GN=Diaph1 PE=1 SV=1	sp|O08808|DIAP1_MOUSE	139 kDa	6	0
ELKS/Rab6-interacting/CAST family member 1 OS=Mus musculus GN=Erc1 PE=1 SV=1	sp|Q99MI1|RB6I2_MOUSE	128 kDa	6	0
Ankyrin repeat domain-containing protein 1 OS=Mus musculus GN=Ankrd1 PE=2 SV=	sp|Q9CR42|ANKR1_MOUSE	36 kDa	6	0
ADP-ribosylation factor-like protein 8A OS=Mus musculus GN=Arl8a PE=2 SV=1	sp|Q8VEH3|ARL8A_MOUSE	21 kDa	6	0
RNA-binding protein 38 OS=Mus musculus GN=Rbm38 PE=1 SV=2	sp|Q62176|RBM38_MOUSE (+2)	25 kDa	6	0
Nuclear factor 1 C-type OS=Homo sapiens GN=NFIC PE=1 SV=2	sp|P08651|NFIC_HUMAN (+1)	56 kDa	6	0
28S ribosomal protein S34, mitochondrial OS=Mus musculus GN=Mrps34 PE=2 SV=1	sp|Q9JIK9|RT34_MOUSE	26 kDa	6	0
Beta-galactosidase OS=Mus musculus GN=Glb1 PE=2 SV=1	sp|P23780|BGAL_MOUSE	73 kDa	6	0
Splicing factor U2AF 35 kDa subunit OS=Homo sapiens GN=U2AF1 PE=1 SV=3	sp|Q01081|U2AF1_HUMAN (+1)	28 kDa	6	0
1-acyl-sn-glycerol-3-phosphate acyltransferase delta OS=Mus musculus GN=Agpat4 P	sp|Q8K4X7|PLCD_MOUSE	44 kDa	6	0
Pre-mRNA-processing-splicing factor 8 OS=Homo sapiens GN=PRPF8 PE=1 SV=2	sp|Q6P2Q9|PRP8_HUMAN (+1)	274 kDa	6	0
Lamin-B1 OS=Mus musculus GN=Lmnb1 PE=1 SV=3	sp|P14733|LMNB1_MOUSE	67 kDa	6	0
2',3'-cyclic-nucleotide 3'-phosphodiesterase OS=Mus musculus GN=Cnp PE=1 SV=3	sp|P16330|CN37_MOUSE	47 kDa	6	0
Cellular nucleic acid-binding protein OS=Mus musculus GN=Cnbp PE=2 SV=2	sp|P53996|CNBP_MOUSE (+1)	20 kDa	6	0
40S ribosomal protein S25 OS=Homo sapiens GN=RPS25 PE=1 SV=1	sp|P62851|RS25_HUMAN (+1)	14 kDa	6	0
28S ribosomal protein S5, mitochondrial OS=Mus musculus GN=Mrps5 PE=2 SV=1	sp|Q99N87|RT05_MOUSE	48 kDa	6	0
Dihydropyrimidinase-related protein 2 OS=Mus musculus GN=Dpysl2 PE=1 SV=2	sp|O08553|DPYL2_MOUSE	62 kDa	6	0
Acetylcholine receptor subunit gamma OS=Mus musculus GN=Chrng PE=2 SV=1	sp|P04760|ACHG_MOUSE	59 kDa	5	0
5-oxoprolinase OS=Mus musculus GN=Oplah PE=2 SV=1	sp|Q8K010|OPLA_MOUSE	138 kDa	5	0
ATP-binding cassette sub-family F member 1 OS=Mus musculus GN=Abcf1 PE=1 SV=1	sp|Q6P542|ABCF1_MOUSE	95 kDa	5	0
Muscleblind-like protein 1 OS=Mus musculus GN=Mbnl1 PE=1 SV=1	sp|Q9JKP5|MBNL1_MOUSE (+1)	37 kDa	5	0
GRIP and coiled-coil domain-containing protein 2 OS=Mus musculus GN=Gcc2 PE=1 S	sp|Q8CHG3|GCC2_MOUSE	194 kDa	5	0
Protein FAM91A1 OS=Homo sapiens GN=FAM91A1 PE=1 SV=3	sp|Q658Y4|F91A1_HUMAN	94 kDa	5	0
Kelch repeat and BTB domain-containing protein 2 OS=Homo sapiens GN=KBTBD2 PE	sp|Q8IY47|KBTB2_HUMAN	71 kDa	5	0
Voltage-dependent anion-selective channel protein 3 OS=Mus musculus GN=Vdac3 P	sp|Q60931|VDAC3_MOUSE (+1)	31 kDa	5	0
Kinesin-like protein KIF1C OS=Mus musculus GN=Kif1c PE=1 SV=2	sp|O35071|KIF1C_MOUSE	122 kDa	5	0
Ras-related protein Rab-18 OS=Mus musculus GN=Rab18 PE=2 SV=2	sp|P35293|RAB18_MOUSE (+1)	23 kDa	5	0
WD repeat-containing protein 5 OS=Homo sapiens GN=WDR5 PE=1 SV=1	sp|P61964|WDR5_HUMAN (+1)	37 kDa	5	0
Protein RCC2 OS=Mus musculus GN=Rcc2 PE=2 SV=1	sp|Q8BK67|RCC2_MOUSE (+1)	56 kDa	5	0
Geranylgeranyl transferase type-2 subunit alpha OS=Mus musculus GN=Rabggta PE=:	sp|Q9JHK4|PGTA_MOUSE	65 kDa	5	0
Transducin beta-like protein 2 OS=Mus musculus GN=Tbl2 PE=2 SV=2	sp|Q9R099|TBL2_MOUSE	50 kDa	5	0
Fibrillin-1 OS=Mus musculus GN=Fbn1 PE=1 SV=1	sp|Q61554|FBN1_MOUSE	312 kDa	5	0
Protein yippee-like 5 OS=Homo sapiens GN=YPEL5 PE=2 SV=1	sp|P62699|YPEL5_HUMAN (+1)	14 kDa	5	0
Casein kinase I isoform alpha OS=Homo sapiens GN=CSNK1A1 PE=1 SV=2	sp|P48729|KC1A_HUMAN (+1)	39 kDa	5	0
STIP1 homology and U box-containing protein 1 OS=Mus musculus GN=Stub1 PE=1 S\	sp|Q9WUD1|CHIP_MOUSE	35 kDa	5	0
Ras-related protein Rab-14 OS=Homo sapiens GN=RAB14 PE=1 SV=4	sp|P61106|RAB14_HUMAN (+1)	24 kDa	5	0
Serine/threonine-protein kinase TAO3 OS=Mus musculus GN=Taok3 PE=1 SV=2	sp|Q8BYC6|TAOK3_MOUSE (+1)	105 kDa	5	0
SLIT-ROBO Rho GTPase-activating protein 2 OS=Homo sapiens GN=SRGAP2 PE=1 SV=:	sp|O75044|FNBP2_HUMAN (+1)	121 kDa	5	0
General transcription factor II-I OS=Homo sapiens GN=GTF2I PE=1 SV=2	sp|P78347|GTF2I_HUMAN (+1)	112 kDa	5	0
S-phase kinase-associated protein 1 OS=Homo sapiens GN=SKP1 PE=1 SV=2	sp|P63208|SKP1_HUMAN (+1)	19 kDa	5	0
28S ribosomal protein S27, mitochondrial OS=Mus musculus GN=Mrps27 PE=2 SV=2	sp|Q8BK72|RT27_MOUSE	48 kDa	5	0
40S ribosomal protein S29 OS=Homo sapiens GN=RPS29 PE=1 SV=2	sp|P62273|RS29_HUMAN (+1)	7 kDa	5	0
tRNA (cytosine(34)-C(5))-methyltransferase OS=Mus musculus GN=Nsun2 PE=1 SV=2	sp|Q1HFZ0|NSUN2_MOUSE	85 kDa	5	0
28S ribosomal protein S35, mitochondrial OS=Mus musculus GN=Mrps35 PE=2 SV=2	sp|Q8BJZ4|RT35_MOUSE	36 kDa	5	0
39S ribosomal protein L40, mitochondrial OS=Mus musculus GN=Mrpl40 PE=2 SV=2	sp|Q9Z2Q5|RM40_MOUSE	24 kDa	5	0
28S ribosomal protein S18b, mitochondrial OS=Mus musculus GN=Mrps18b PE=2 SV=	sp|Q99N84|RT18B_MOUSE	29 kDa	5	0
Peroxiredoxin-2 OS=Homo sapiens GN=PRDX2 PE=1 SV=5	sp|P32119|PRDX2_HUMAN	22 kDa	5	0
Protein LYRIC OS=Mus musculus GN=Mtdh PE=1 SV=1	sp|Q80WJ7|LYRIC_MOUSE	64 kDa	4	0
SH3 and PX domain-containing protein 2A OS=Mus musculus GN=Sh3pxd2a PE=1 SV=	sp|O89032|SPD2A_MOUSE	124 kDa	4	0
Ankyrin repeat domain-containing protein 17 OS=Homo sapiens GN=ANKRD17 PE=1 !	sp|O75179|ANR17_HUMAN (+1)	274 kDa	4	0
Low-density lipoprotein receptor-related protein 4 OS=Mus musculus GN=Lrp4 PE=1	sp|Q8VI56|LRP4_MOUSE	212 kDa	4	0
Dephospho-CoA kinase domain-containing protein OS=Mus musculus GN=Dcakd PE=	sp|Q8BHC4|DCAKD_MOUSE	26 kDa	4	0
Endoplasmic reticulum-Golgi intermediate compartment protein 2 OS=Mus musculus	sp|Q9CR89|ERGI2_MOUSE	42 kDa	4	0
Protein FAM98A OS=Mus musculus GN=Fam98a PE=2 SV=1	sp|Q3TJZ6|FA98A_MOUSE	55 kDa	4	0
Procollagen-lysine,2-oxoglutarate 5-dioxygenase 3 OS=Mus musculus GN=Plod3 PE=1	sp|Q9R0E1|PLOD3_MOUSE	85 kDa	4	0
Alpha-1-syntrophin OS=Homo sapiens GN=SNTA1 PE=1 SV=1	sp|Q13424|SNTA1_HUMAN	54 kDa	4	0
Mothers against decapentaplegic homolog 3 OS=Homo sapiens GN=SMAD3 PE=1 SV=	sp|P84022|SMAD3_HUMAN (+1)	48 kDa	4	0
BRISC complex subunit Abro1 OS=Mus musculus GN=Fam175b PE=2 SV=1	sp|Q3TCJ1|F175B_MOUSE	47 kDa	4	0
Protein cordon-bleu OS=Mus musculus GN=Cobl PE=1 SV=1	sp|Q5NBX1|COBL_MOUSE	144 kDa	4	0
Trafficking protein particle complex subunit 5 OS=Mus musculus GN=Trappc5 PE=1 S\	sp|Q9CQA1|TPPC5_MOUSE	21 kDa	4	0
Beta-parvin OS=Mus musculus GN=Parvb PE=1 SV=1	sp|Q9ES46|PARVB_MOUSE	42 kDa	4	0
Spermine synthase OS=Mus musculus GN=Sms PE=1 SV=1	sp|P97355|SPSY_MOUSE	41 kDa	4	0
Mitogen-activated protein kinase kinase kinase MLT OS=Mus musculus GN=Mltk PE=:	sp|Q9ESL4|MLTK_MOUSE (+1)	92 kDa	4	0
Kelch-like protein 30 OS=Mus musculus GN=Klhl30 PE=2 SV=1	sp|Q8C3F7|KLH30_MOUSE	65 kDa	4	0
Ras-related protein Rab-6A OS=Homo sapiens GN=RAB6A PE=1 SV=3	sp|P20340|RAB6A_HUMAN (+1)	24 kDa	4	0
A-kinase anchor protein 13 OS=Homo sapiens GN=AKAP13 PE=1 SV=2	sp|Q12802|AKP13_HUMAN	308 kDa	4	0
Fragile X mental retardation protein 1 homolog OS=Mus musculus GN=Fmr1 PE=1 SV	sp|P35922|FMR1_MOUSE	69 kDa	4	0
Protein arginine N-methyltransferase 2 OS=Mus musculus GN=Prmt2 PE=1 SV=1	sp|Q9R144|ANM2_MOUSE	50 kDa	4	0
Cytosolic acyl coenzyme A thioester hydrolase OS=Homo sapiens GN=ACOT7 PE=1 SV	sp|O00154|BACH_HUMAN	42 kDa	4	0
ATP-binding cassette sub-family B member 7, mitochondrial OS=Homo sapiens GN=A	sp|O75027|ABCB7_HUMAN (+1)	83 kDa	4	0
Lipopolysaccharide-responsive and beige-like anchor protein OS=Mus musculus GN=I	sp|Q9ESE1|LRBA_MOUSE	317 kDa	4	0
Serine/arginine-rich splicing factor 2 OS=Homo sapiens GN=SRSF2 PE=1 SV=4	sp|Q01130|SRSF2_HUMAN (+1)	25 kDa	4	0
Mitochondrial fission factor OS=Mus musculus GN=Mff PE=1 SV=1	sp|Q6PCP5|MFF_MOUSE	33 kDa	4	0
Trimethyllysine dioxygenase, mitochondrial OS=Mus musculus GN=Tmlhe PE=2 SV=2	sp|Q91ZE0|TMLH_MOUSE	50 kDa	4	0
Ubiquitin-associated protein 2-like OS=Mus musculus GN=Ubap2l PE=1 SV=1	sp|Q80X50|UBP2L_MOUSE	117 kDa	4	0

UDP-N-acetylglucosamine--dolichyl-phosphate N-acetylglucosaminephosphotransfer	sp|P42867|GPT_MOUSE	46 kDa	4	0
Isobutyryl-CoA dehydrogenase, mitochondrial OS=Mus musculus GN=Acad8 PE=2 SV	sp|Q9D7B6|ACAD8_MOUSE	45 kDa	4	0
NFX1-type zinc finger-containing protein 1 OS=Mus musculus GN=Znfx1 PE=2 SV=3	sp|Q8R151|ZNFX1_MOUSE	219 kDa	4	0
Ubiquitin-protein ligase E3A OS=Mus musculus GN=Ube3a PE=2 SV=1	sp|O08759|UBE3A_MOUSE	101 kDa	4	0
60S ribosomal protein L35 OS=Homo sapiens GN=RPL35 PE=1 SV=2	sp|P42766|RL35_HUMAN (+1)	15 kDa	4	0
Plectin OS=Mus musculus GN=Plec PE=1 SV=2	sp|Q9QXS1|PLEC_MOUSE	534 kDa	2389	1401
Myosin-9 OS=Mus musculus GN=Myh9 PE=1 SV=4	sp|Q8VDD5|MYH9_MOUSE	226 kDa	1369	2189
SH3 domain-binding protein 2 OS=Mus musculus GN=Sh3bp2 PE=1 SV=1	sp|Q06649|3BP2_MOUSE	62 kDa	1635	1705
Myosin-3 OS=Mus musculus GN=Myh3 PE=2 SV=2	sp|P13541|MYH3_MOUSE	224 kDa	602	1759
Vimentin OS=Mus musculus GN=Vim PE=1 SV=3	sp|P20152|VIME_MOUSE	54 kDa	876	489
Myosin-1 OS=Mus musculus GN=Myh1 PE=1 SV=1	sp|Q5SX40|MYH1_MOUSE	223 kDa	536	1579
Nestin OS=Mus musculus GN=Nes PE=1 SV=1	sp|Q6P5H2|NEST_MOUSE	207 kDa	397	232
Actin, cytoplasmic 2 OS=Mus musculus GN=Actg1 PE=1 SV=1	sp|P63260|ACTG_MOUSE (+1)	42 kDa	210	402
Glyceraldehyde-3-phosphate dehydrogenase OS=Mus musculus GN=Gapdh PE=1 SV=	sp|P16858|G3P_MOUSE	36 kDa	446	147
Myosin-10 OS=Mus musculus GN=Myh10 PE=1 SV=2	sp|Q61879|MYH10_MOUSE	229 kDa	472	700
Keratin, type II cytoskeletal 1 OS=Homo sapiens GN=KRT1 PE=1 SV=6	sp|P04264|K2C1_HUMAN	66 kDa	229	324
Sarcoplasmic/endoplasmic reticulum calcium ATPase 1 OS=Mus musculus GN=Atp2a	sp|Q8R429|AT2A1_MOUSE	109 kDa	264	258
Myosin-7 OS=Mus musculus GN=Myh7 PE=1 SV=1	sp|Q91Z83|MYH7_MOUSE	223 kDa	318	959
Cytoplasmic dynein 1 heavy chain 1 OS=Mus musculus GN=Dync1h1 PE=1 SV=2	sp|Q9JHU4|DYHC1_MOUSE	532 kDa	295	184
Elongation factor 1-alpha 1 OS=Mus musculus GN=Eef1a1 PE=1 SV=3	sp|P10126|EF1A1_MOUSE	50 kDa	258	211
Myosin-8 OS=Mus musculus GN=Myh8 PE=1 SV=2	sp|P13542|MYH8_MOUSE	223 kDa	389	1561
Tubulin alpha-1B chain OS=Mus musculus GN=Tuba1b PE=1 SV=2	sp|P05213|TBA1B_MOUSE (+1)	50 kDa	220	158
Heat shock protein HSP 90-beta OS=Homo sapiens GN=HSP90AB1 PE=1 SV=4	sp|P08238|HS90B_HUMAN (+1)	83 kDa	243	111
Tubulin beta-2B chain OS=Homo sapiens GN=TUBB2B PE=1 SV=1	sp|Q9BVA1|TBB2B_HUMAN (+1)	50 kDa	201	151
6-phosphofructokinase, muscle type OS=Mus musculus GN=Pfkm PE=1 SV=3	sp|P47857|K6PF_MOUSE	85 kDa	221	105
Heat shock cognate 71 kDa protein OS=Mus musculus GN=Hspa8 PE=1 SV=1	sp|P63017|HSP7C_MOUSE	71 kDa	186	132
Elongation factor 2 OS=Mus musculus GN=Eef2 PE=1 SV=2	sp|P58252|EF2_MOUSE	95 kDa	182	117
Keratin, type I cytoskeletal 9 OS=Homo sapiens GN=KRT9 PE=1 SV=3	sp|P35527|K1C9_HUMAN	62 kDa	121	177
60S ribosomal protein L3 OS=Mus musculus GN=Rpl3 PE=2 SV=3	sp|P27659|RL3_MOUSE	46 kDa	222	18
Tropomyosin beta chain OS=Mus musculus GN=Tpm2 PE=1 SV=1	sp|P58774|TPM2_MOUSE	33 kDa	57	163
Myosin-Ic OS=Mus musculus GN=Myo1c PE=1 SV=2	sp|Q9WTI7|MYO1C_MOUSE	122 kDa	23	182
Actin, alpha skeletal muscle OS=Homo sapiens GN=ACTA1 PE=1 SV=1	sp|P68133|ACTS_HUMAN (+1)	42 kDa	187	352
Prelamin-A/C OS=Mus musculus GN=Lmna PE=1 SV=2	sp|P48678|LMNA_MOUSE	74 kDa	104	89
DNA damage-binding protein 1 OS=Mus musculus GN=Ddb1 PE=1 SV=2	sp|Q3U1J4|DDB1_MOUSE	127 kDa	114	80
Myosin-4 OS=Mus musculus GN=Myh4 PE=1 SV=1	sp|Q5SX39|MYH4_MOUSE	223 kDa	474	1402
Glycogen phosphorylase, muscle form OS=Mus musculus GN=Pygm PE=1 SV=3	sp|Q9WUB3|PYGM_MOUSE	97 kDa	108	68
Stress-70 protein, mitochondrial OS=Mus musculus GN=Hspa9 PE=1 SV=2	sp|P38647|GRP75_MOUSE	74 kDa	101	74
Gelsolin OS=Mus musculus GN=Gsn PE=1 SV=3	sp|P13020|GELS_MOUSE	86 kDa	54	115
SH3 domain-binding protein 2 OS=Homo sapiens GN=SH3BP2 PE=1 SV=2	sp|P78314|3BP2_HUMAN	62 kDa	666	609
Sarcoplasmic/endoplasmic reticulum calcium ATPase 2 OS=Mus musculus GN=Atp2a	sp|O55143|AT2A2_MOUSE	115 kDa	189	165
Pyruvate kinase isozymes M1/M2 OS=Mus musculus GN=Pkm2 PE=1 SV=4	sp|P52480|KPYM_MOUSE	58 kDa	69	88
Myosin-binding protein C, slow-type OS=Homo sapiens GN=MYBPC1 PE=1 SV=2	sp|Q00872|MYPC1_HUMAN	128 kDa	34	113
Heat shock 70 kDa protein 1A/1B OS=Homo sapiens GN=HSPA1A PE=1 SV=5	sp|P08107|HSP71_HUMAN	70 kDa	98	89
ATP synthase subunit alpha, mitochondrial OS=Mus musculus GN=Atp5a1 PE=1 SV=1	sp|Q03265|ATPA_MOUSE	60 kDa	31	109
ADP/ATP translocase 2 OS=Mus musculus GN=Slc25a5 PE=1 SV=3	sp|P51881|ADT2_MOUSE	33 kDa	32	101
Ubiquitin-40S ribosomal protein S27a OS=Mus musculus GN=Rps27a PE=1 SV=2	sp|P62983|RS27A_MOUSE	18 kDa	55	78
78 kDa glucose-regulated protein OS=Mus musculus GN=Hspa5 PE=1 SV=3	sp|P20029|GRP78_MOUSE	72 kDa	72	67
Protein flightless-1 homolog OS=Mus musculus GN=Flii PE=1 SV=1	sp|Q9JJ28|FLII_MOUSE	145 kDa	69	61
UDP-glucose:glycoprotein glucosyltransferase 1 OS=Mus musculus GN=Uggt1 PE=1 S	sp|Q6P5E4|UGGG1_MOUSE	176 kDa	104	24
Myosin regulatory light chain 2, skeletal muscle isoform OS=Mus musculus GN=Mylp	sp|P97457|MLRS_MOUSE	19 kDa	44	81
Clathrin heavy chain 1 OS=Homo sapiens GN=CLTC PE=1 SV=5	sp|Q00610|CLH1_HUMAN (+1)	192 kDa	74	48
40S ribosomal protein S4, X isoform OS=Homo sapiens GN=RPS4X PE=1 SV=2	sp|P62701|RS4X_HUMAN (+1)	30 kDa	97	17
Heterogeneous nuclear ribonucleoprotein H2 OS=Mus musculus GN=Hnrnph2 PE=1 S	sp|P70333|HNRH2_MOUSE	49 kDa	79	35
ATP-dependent RNA helicase DDX3X OS=Mus musculus GN=Ddx3x PE=1 SV=3	sp|Q62167|DDX3X_MOUSE	73 kDa	88	21
Myosin light chain 1/3, skeletal muscle isoform OS=Mus musculus GN=Myl1 PE=1 SV	sp|P05977|MYL1_MOUSE	21 kDa	34	79
Nuclear cap-binding protein subunit 1 OS=Mus musculus GN=Ncbp1 PE=1 SV=2	sp|Q3UYV9|NCBP1_MOUSE	92 kDa	4	0
Protein disulfide-isomerase OS=Mus musculus GN=P4hb PE=1 SV=2	sp|P09103|PDIA1_MOUSE	57 kDa	62	39
Heat shock protein beta-1 OS=Mus musculus GN=Hspb1 PE=1 SV=3	sp|P14602|HSPB1_MOUSE	23 kDa	58	46
Polyadenylate-binding protein 1 OS=Mus musculus GN=Pabpc1 PE=1 SV=2	sp|P29341|PABP1_MOUSE	71 kDa	74	25
40S ribosomal protein S3a OS=Mus musculus GN=Rps3a PE=1 SV=3	sp|P97351|RS3A_MOUSE	30 kDa	83	17
60S ribosomal protein L4 OS=Mus musculus GN=Rpl4 PE=1 SV=3	sp|Q9D8E6|RL4_MOUSE	47 kDa	98	2
Alpha-crystallin B chain OS=Mus musculus GN=Cryab PE=1 SV=2	sp|P23927|CRYAB_MOUSE	20 kDa	74	27
Tropomyosin alpha-1 chain OS=Mus musculus GN=Tpm1 PE=1 SV=1	sp|P58771|TPM1_MOUSE	33 kDa	44	136
Vinculin OS=Mus musculus GN=Vcl PE=1 SV=4	sp|Q64727|VINC_MOUSE	117 kDa	49	48
Trifunctional enzyme subunit alpha, mitochondrial OS=Mus musculus GN=Hadha PE=	sp|Q8BMS1|ECHA_MOUSE	83 kDa	70	24
DnaJ homolog subfamily B member 11 OS=Mus musculus GN=Dnajb11 PE=1 SV=1	sp|Q99KV1|DJB11_MOUSE	41 kDa	49	40
Elongation factor Tu, mitochondrial OS=Mus musculus GN=Tufm PE=1 SV=1	sp|Q8BFR5|EFTU_MOUSE	50 kDa	56	34
Tubulin beta-6 chain OS=Mus musculus GN=Tubb6 PE=1 SV=1	sp|Q922F4|TBB6_MOUSE	50 kDa	160	105
Hexokinase-2 OS=Mus musculus GN=Hk2 PE=1 SV=1	sp|O08528|HXK2_MOUSE	103 kDa	66	25
60S ribosomal protein L7 OS=Mus musculus GN=Rpl7 PE=2 SV=2	sp|P14148|RL7_MOUSE	31 kDa	84	3
Bifunctional aminoacyl-tRNA synthetase OS=Mus musculus GN=Eprs PE=2 SV=4	sp|Q8CGC7|SYEP_MOUSE	170 kDa	58	29
60S ribosomal protein L13 OS=Mus musculus GN=Rpl13 PE=2 SV=3	sp|P47963|RL13_MOUSE	24 kDa	79	8
Kelch repeat and BTB domain-containing protein 10 OS=Homo sapiens GN=KBTBD10	sp|O60662|KBTBA_HUMAN	68 kDa	59	24
Probable ATP-dependent RNA helicase DDX5 OS=Mus musculus GN=Ddx5 PE=1 SV=2	sp|Q61656|DDX5_MOUSE	69 kDa	66	23
3-ketoacyl-CoA thiolase, mitochondrial OS=Mus musculus GN=Acaa2 PE=1 SV=3	sp|Q8BWT1|THIM_MOUSE	42 kDa	33	35
26S proteasome non-ATPase regulatory subunit 1 OS=Mus musculus GN=Psmd1 PE=	sp|Q3TXS7|PSMD1_MOUSE	106 kDa	45	36
Ig gamma-3 chain C region OS=Homo sapiens GN=IGHG3 PE=1 SV=2	sp|P01860|IGHG3_HUMAN	41 kDa	40	39

Microtubule-associated protein 1B OS=Mus musculus GN=Map1b PE=1 SV=2	sp|P14873|MAP1B_MOUSE	270 kDa	39	41
Nexilin OS=Mus musculus GN=Nexn PE=1 SV=3	sp|Q7TPW1|NEXN_MOUSE	72 kDa	26	53
Serpin B6 OS=Mus musculus GN=Serpinb6 PE=2 SV=1	sp|Q60854|SPB6_MOUSE	43 kDa	56	18
ATP synthase subunit beta, mitochondrial OS=Mus musculus GN=Atp5b PE=1 SV=2	sp|P56480|ATPB_MOUSE	56 kDa	5	71
40S ribosomal protein S3 OS=Mus musculus GN=Rps3 PE=1 SV=1	sp|P62908|RS3_MOUSE	27 kDa	58	17
Serpin H1 OS=Mus musculus GN=Serpinh1 PE=1 SV=3	sp|P19324|SERPH_MOUSE	47 kDa	36	38
Myosin light chain 4 OS=Mus musculus GN=Myl4 PE=2 SV=3	sp|P09541|MYL4_MOUSE	21 kDa	31	48
Transitional endoplasmic reticulum ATPase OS=Homo sapiens GN=VCP PE=1 SV=4	sp|P55072|TERA_HUMAN (+1)	89 kDa	22	51
Protein-glutamine gamma-glutamyltransferase 2 OS=Mus musculus GN=Tgm2 PE=1 S	sp|P21981|TGM2_MOUSE	77 kDa	55	14
Beta-enolase OS=Mus musculus GN=Eno3 PE=1 SV=3	sp|P21550|ENOB_MOUSE	47 kDa	8	64
COMM domain-containing protein 4 OS=Mus musculus GN=Commd4 PE=2 SV=1	sp|Q9CQ02|COMD4_MOUSE	22 kDa	4	0
DnaJ homolog subfamily A member 2 OS=Mus musculus GN=Dnaja2 PE=1 SV=1	sp|Q9QYJ0|DNJA2_MOUSE	46 kDa	39	32
Spectrin beta chain, brain 1 OS=Mus musculus GN=Sptbn1 PE=1 SV=2	sp|Q62261|SPTB2_MOUSE	274 kDa	43	29
Myc box-dependent-interacting protein 1 OS=Mus musculus GN=Bin1 PE=1 SV=1	sp|O08539|BIN1_MOUSE	64 kDa	40	30
40S ribosomal protein S11 OS=Homo sapiens GN=RPS11 PE=1 SV=3	sp|P62280|RS11_HUMAN (+1)	18 kDa	55	13
Coatomer subunit beta' OS=Mus musculus GN=Copb2 PE=2 SV=2	sp|O55029|COPB2_MOUSE	102 kDa	39	31
Isoleucyl-tRNA synthetase, cytoplasmic OS=Mus musculus GN=Iars PE=2 SV=2	sp|Q8BU30|SYIC_MOUSE	144 kDa	43	26
60S ribosomal protein L8 OS=Homo sapiens GN=RPL8 PE=1 SV=2	sp|P62917|RL8_HUMAN (+1)	28 kDa	61	3
Myosin-binding protein H OS=Mus musculus GN=Mybph PE=1 SV=2	sp|P70402|MYBPH_MOUSE	53 kDa	44	22
60 kDa heat shock protein, mitochondrial OS=Mus musculus GN=Hspd1 PE=1 SV=1	sp|P63038|CH60_MOUSE	61 kDa	22	42
Leucine-rich PPR motif-containing protein, mitochondrial OS=Mus musculus GN=Lrpp	sp|Q6PB66|LPPRC_MOUSE	157 kDa	58	6
Eukaryotic translation elongation factor 1 epsilon-1 OS=Mus musculus GN=Eef1e1 PE	sp|Q9D1M4|MCA3_MOUSE	20 kDa	4	0
60S ribosomal protein L5 OS=Mus musculus GN=Rpl5 PE=1 SV=3	sp|P47962|RL5_MOUSE	34 kDa	50	13
Troponin T, fast skeletal muscle OS=Mus musculus GN=Tnnt3 PE=1 SV=3	sp|Q9QZ47|TNNT3_MOUSE	32 kDa	14	51
Sarcalumenin OS=Mus musculus GN=Srl PE=1 SV=1	sp|Q7TQ48|SRCA_MOUSE	99 kDa	30	36
40S ribosomal protein S8 OS=Homo sapiens GN=RPS8 PE=1 SV=2	sp|P62241|RS8_HUMAN (+1)	24 kDa	62	2
D-3-phosphoglycerate dehydrogenase OS=Mus musculus GN=Phgdh PE=1 SV=3	sp|Q61753|SERA_MOUSE	57 kDa	28	36
Spectrin alpha chain, brain OS=Mus musculus GN=Sptan1 PE=1 SV=4	sp|P16546|SPTA2_MOUSE	285 kDa	31	33
Cysteine desulfurase, mitochondrial OS=Mus musculus GN=Nfs1 PE=2 SV=2	sp|Q9Z1J3|NFS1_MOUSE	50 kDa	4	0
Annexin A6 OS=Mus musculus GN=Anxa6 PE=1 SV=3	sp|P14824|ANXA6_MOUSE	76 kDa	2	60
Polymerase I and transcript release factor OS=Mus musculus GN=Ptrf PE=1 SV=1	sp|O54724|PTRF_MOUSE	44 kDa	49	14
40S ribosomal protein S16 OS=Mus musculus GN=Rps16 PE=2 SV=4	sp|P14131|RS16_MOUSE (+1)	16 kDa	45	15
60S ribosomal protein L10a OS=Homo sapiens GN=RPL10A PE=1 SV=2	sp|P62906|RL10A_HUMAN	25 kDa	50	11
40S ribosomal protein S13 OS=Homo sapiens GN=RPS13 PE=1 SV=2	sp|P62277|RS13_HUMAN (+1)	17 kDa	44	14
40S ribosomal protein S9 OS=Homo sapiens GN=RPS9 PE=1 SV=3	sp|P46781|RS9_HUMAN (+1)	23 kDa	59	4
Eukaryotic translation initiation factor 2 subunit 3, X-linked OS=Mus musculus GN=Eif	sp|Q9Z0N1|IF2G_MOUSE	51 kDa	25	36
Leucyl-tRNA synthetase, cytoplasmic OS=Mus musculus GN=Lars PE=2 SV=2	sp|Q8BMJ2|SYLC_MOUSE	134 kDa	32	31
DNA-binding protein A OS=Mus musculus GN=Csda PE=1 SV=2	sp|Q9JKB3|DBPA_MOUSE	39 kDa	52	9
60S ribosomal protein L18a OS=Mus musculus GN=Rpl18a PE=1 SV=1	sp|P62717|RL18A_MOUSE	21 kDa	55	5
60S ribosomal protein L10 OS=Homo sapiens GN=RPL10 PE=1 SV=4	sp|P27635|RL10_HUMAN (+1)	25 kDa	49	8
Actin-related protein 2/3 complex subunit 1B OS=Mus musculus GN=Arpc1b PE=1 SV	sp|Q9WV32|ARC1B_MOUSE	41 kDa	31	30
Myosin light chain 6B OS=Mus musculus GN=Myl6b PE=2 SV=1	sp|Q8CI43|MYL6B_MOUSE	23 kDa	12	45
60S ribosomal protein L14 OS=Mus musculus GN=Rpl14 PE=2 SV=3	sp|Q9CR57|RL14_MOUSE	24 kDa	52	7
Heterogeneous nuclear ribonucleoprotein K OS=Homo sapiens GN=HNRNPK PE=1 SV	sp|P61978|HNRPK_HUMAN (+1)	51 kDa	45	17
Splicing factor, proline- and glutamine-rich OS=Mus musculus GN=Sfpq PE=1 SV=1	sp|Q8VIJ6|SFPQ_MOUSE	75 kDa	38	19
Heterogeneous nuclear ribonucleoprotein U OS=Mus musculus GN=Hnrnpu PE=1 SV=	sp|Q8VEK3|HNRPU_MOUSE	88 kDa	53	5
Guanine nucleotide-binding protein subunit beta-2-like 1 OS=Homo sapiens GN=GNB	sp|P63244|GBLP_HUMAN (+1)	35 kDa	35	22
CAD protein OS=Homo sapiens GN=CAD PE=1 SV=3	sp|P27708|PYR1_HUMAN	243 kDa	41	25
Heterogeneous nuclear ribonucleoprotein F OS=Mus musculus GN=Hnrnpf PE=1 SV=3	sp|Q9Z2X1|HNRPF_MOUSE	46 kDa	53	23
Peptidyl-prolyl cis-trans isomerase A OS=Mus musculus GN=Ppia PE=1 SV=2	sp|P17742|PPIA_MOUSE	18 kDa	13	43
Ryanodine receptor 1 OS=Homo sapiens GN=RYR1 PE=1 SV=3	sp|P21817|RYR1_HUMAN	565 kDa	28	26
Heterogeneous nuclear ribonucleoprotein M OS=Mus musculus GN=Hnrnpm PE=1 SV	sp|Q9D0E1|HNRPM_MOUSE	78 kDa	41	14
Probable ATP-dependent RNA helicase DDX17 OS=Mus musculus GN=Ddx17 PE=2 SV	sp|Q501J6|DDX17_MOUSE (+1)	72 kDa	67	12
Major vault protein OS=Mus musculus GN=Mvp PE=1 SV=4	sp|Q9EQK5|MVP_MOUSE	96 kDa	11	44
Actin-related protein 2/3 complex subunit 2 OS=Mus musculus GN=Arpc2 PE=1 SV=3	sp|Q9CVB6|ARPC2_MOUSE	34 kDa	26	30
T-complex protein 1 subunit epsilon OS=Mus musculus GN=Cct5 PE=1 SV=1	sp|P80316|TCPE_MOUSE	60 kDa	29	27
Eukaryotic initiation factor 4A-I OS=Homo sapiens GN=EIF4A1 PE=1 SV=1	sp|P60842|IF4A1_HUMAN (+1)	46 kDa	24	30
Angiomotin OS=Mus musculus GN=Amot PE=1 SV=3	sp|Q8VHG2|AMOT_MOUSE	121 kDa	0	55
Desmoplakin OS=Homo sapiens GN=DSP PE=1 SV=3	sp|P15924|DESP_HUMAN	332 kDa	18	28
Non-POU domain-containing octamer-binding protein OS=Homo sapiens GN=NONO	sp|Q15233|NONO_HUMAN (+1)	54 kDa	37	21
Fructose-bisphosphate aldolase A OS=Mus musculus GN=Aldoa PE=1 SV=2	sp|P05064|ALDOA_MOUSE	39 kDa	16	36
26S proteasome non-ATPase regulatory subunit 2 OS=Homo sapiens GN=PSMD2 PE=	sp|Q13200|PSMD2_HUMAN (+1)	100 kDa	33	18
Actin-related protein 2 OS=Homo sapiens GN=ACTR2 PE=1 SV=1	sp|P61160|ARP2_HUMAN (+1)	45 kDa	34	28
Tripartite motif-containing protein 54 OS=Mus musculus GN=Trim54 PE=1 SV=1	sp|Q9ERP3|TRI54_MOUSE	41 kDa	9	42
Aspartyl-tRNA synthetase, cytoplasmic OS=Mus musculus GN=Dars PE=2 SV=2	sp|Q922B2|SYDC_MOUSE	57 kDa	37	19
Alpha-enolase OS=Mus musculus GN=Eno1 PE=1 SV=3	sp|P17182|ENOA_MOUSE	47 kDa	13	61
Deoxyuridine 5'-triphosphate nucleotidohydrolase, mitochondrial OS=Homo sapiens	sp|P33316|DUT_HUMAN	27 kDa	4	0
E3 ubiquitin-protein ligase UBR4 OS=Mus musculus GN=Ubr4 PE=1 SV=1	sp|A2AN08|UBR4_MOUSE	572 kDa	33	16
Myosin regulatory light chain 12A OS=Homo sapiens GN=MYL12A PE=1 SV=2	sp|P19105|ML12A_HUMAN	20 kDa	14	39
DnaJ homolog subfamily A member 1 OS=Mus musculus GN=Dnaja1 PE=1 SV=1	sp|P63037|DNJA1_MOUSE	45 kDa	28	25
60S ribosomal protein L35a OS=Mus musculus GN=Rpl35a PE=2 SV=2	sp|O55142|RL35A_MOUSE (+1)	13 kDa	27	22
60S ribosomal protein L26 OS=Homo sapiens GN=RPL26 PE=1 SV=1	sp|P61254|RL26_HUMAN (+1)	17 kDa	49	4
Alpha-actinin-2 OS=Homo sapiens GN=ACTN2 PE=1 SV=1	sp|P35609|ACTN2_HUMAN	104 kDa	5	47
60S ribosomal protein L13a OS=Mus musculus GN=Rpl13a PE=1 SV=4	sp|P19253|RL13A_MOUSE	23 kDa	47	2
Apoptosis-inducing factor 1, mitochondrial OS=Mus musculus GN=Aifm1 PE=1 SV=1	sp|Q9Z0X1|AIFM1_MOUSE	67 kDa	23	25
Actin-related protein 3 OS=Mus musculus GN=Actr3 PE=1 SV=3	sp|Q99JY9|ARP3_MOUSE	47 kDa	23	27
14-3-3 protein epsilon OS=Homo sapiens GN=YWHAE PE=1 SV=1	sp|P62258|1433E_HUMAN (+1)	29 kDa	19	28

Coatomer subunit beta OS=Mus musculus GN=Copb1 PE=1 SV=1	sp|Q9JIF7|COPB_MOUSE	107 kDa	30	18
26S protease regulatory subunit 10B OS=Homo sapiens GN=PSMC6 PE=1 SV=1	sp|P62333|PRS10_HUMAN (+1)	44 kDa	32	15
Obscurin OS=Mus musculus GN=Obscn PE=2 SV=2	sp|A2AAJ9|OBSCN_MOUSE	966 kDa	27	21
Trifunctional enzyme subunit beta, mitochondrial OS=Mus musculus GN=Hadhb PE=1	sp|Q99JY0|ECHB_MOUSE	51 kDa	27	21
60S acidic ribosomal protein P0 OS=Mus musculus GN=Rplp0 PE=1 SV=3	sp|P14869|RLA0_MOUSE	34 kDa	22	26
Peroxiredoxin-1 OS=Mus musculus GN=Prdx1 PE=1 SV=1	sp|P35700|PRDX1_MOUSE	22 kDa	23	23
Fatty acid synthase OS=Mus musculus GN=Fasn PE=1 SV=2	sp|P19096|FAS_MOUSE	272 kDa	26	25
Galectin-1 OS=Mus musculus GN=Lgals1 PE=1 SV=3	sp|P16045|LEG1_MOUSE	15 kDa	21	25
60S ribosomal protein L27 OS=Homo sapiens GN=RPL27 PE=1 SV=2	sp|P61353|RL27_HUMAN (+1)	16 kDa	30	17
Hemoglobin subunit beta-2 OS=Mus musculus GN=Hbb-b2 PE=1 SV=2	sp|P02089|HBB2_MOUSE	16 kDa	19	23
Troponin I, fast skeletal muscle OS=Mus musculus GN=Tnni2 PE=1 SV=2	sp|P13412|TNNI2_MOUSE	21 kDa	11	28
ATP synthase subunit O, mitochondrial OS=Mus musculus GN=Atp5o PE=1 SV=1	sp|Q9DB20|ATPO_MOUSE	23 kDa	18	27
Cullin-7 OS=Mus musculus GN=Cul7 PE=1 SV=2	sp|Q8VE73|CUL7_MOUSE	192 kDa	31	15
Poly(rC)-binding protein 1 OS=Mus musculus GN=Pcbp1 PE=1 SV=1	sp|P60335|PCBP1_MOUSE (+1)	37 kDa	35	13
Heat shock protein HSP 90-alpha OS=Homo sapiens GN=HSP90AA1 PE=1 SV=5	sp|P07900|HS90A_HUMAN	85 kDa	104	56
Eukaryotic translation initiation factor 2 subunit 1 OS=Mus musculus GN=Eif2s1 PE=1	sp|Q6ZWX6|IF2A_MOUSE	36 kDa	27	17
Eukaryotic translation initiation factor 3 subunit D OS=Mus musculus GN=Eif3d PE=1	sp|O70194|EIF3D_MOUSE	64 kDa	30	11
Endoplasmin OS=Mus musculus GN=Hsp90b1 PE=1 SV=2	sp|P08113|ENPL_MOUSE	92 kDa	19	30
ADP-ribosylation factor 1 OS=Homo sapiens GN=ARF1 PE=1 SV=2	sp|P84077|ARF1_HUMAN (+1)	21 kDa	15	27
Aconitate hydratase, mitochondrial OS=Mus musculus GN=Aco2 PE=1 SV=1	sp|Q99KI0|ACON_MOUSE	85 kDa	6	37
L-lactate dehydrogenase A chain OS=Mus musculus GN=Ldha PE=1 SV=3	sp|P06151|LDHA_MOUSE	36 kDa	14	27
T-complex protein 1 subunit eta OS=Mus musculus GN=Cct7 PE=1 SV=1	sp|P80313|TCPH_MOUSE	60 kDa	18	26
Coronin-1C OS=Mus musculus GN=Coro1c PE=1 SV=2	sp|Q9WUM4|COR1C_MOUSE	53 kDa	0	43
40S ribosomal protein S7 OS=Homo sapiens GN=RPS7 PE=1 SV=1	sp|P62081|RS7_HUMAN (+1)	22 kDa	37	6
PRA1 family protein 3 OS=Mus musculus GN=Arl6ip5 PE=1 SV=2	sp|Q8R5J9|PRAF3_MOUSE	22 kDa	17	24
Pre-B-cell leukemia transcription factor-interacting protein 1 OS=Mus musculus GN=P	sp|Q3TVI8|PBIP1_MOUSE	81 kDa	24	13
ATP-dependent RNA helicase A OS=Homo sapiens GN=DHX9 PE=1 SV=4	sp|Q08211|DHX9_HUMAN	141 kDa	38	4
Pyruvate dehydrogenase E1 component subunit alpha, somatic form, mitochondrial (	sp|P35486|ODPA_MOUSE	43 kDa	16	26
T-complex protein 1 subunit zeta OS=Mus musculus GN=Cct6a PE=1 SV=3	sp|P80317|TCPZ_MOUSE	58 kDa	13	28
Carbamoyl-phosphate synthase [ammonia], mitochondrial OS=Mus musculus GN=Cp:	sp|Q8C196|CPSM_MOUSE	165 kDa	14	25
60S ribosomal protein L12 OS=Homo sapiens GN=RPL12 PE=1 SV=1	sp|P30050|RL12_HUMAN (+1)	18 kDa	26	15
Phosphate carrier protein, mitochondrial OS=Mus musculus GN=Slc25a3 PE=1 SV=1	sp|Q8VEM8|MPCP_MOUSE	40 kDa	10	27
Tubulin beta chain OS=Homo sapiens GN=TUBB PE=1 SV=2	sp|P07437|TBB5_HUMAN (+1)	50 kDa	148	134
Reticulon-4 OS=Mus musculus GN=Rtn4 PE=1 SV=2	sp|Q99P72|RTN4_MOUSE	127 kDa	25	13
Triosephosphate isomerase OS=Mus musculus GN=Tpi1 PE=1 SV=3	sp|P17751|TPIS_MOUSE	27 kDa	11	29
Heat shock protein beta-8 OS=Mus musculus GN=Hspb8 PE=1 SV=1	sp|Q9JK92|HSPB8_MOUSE	22 kDa	35	4
40S ribosomal protein S14 OS=Homo sapiens GN=RPS14 PE=1 SV=3	sp|P62263|RS14_HUMAN (+1)	16 kDa	29	10
28S ribosomal protein S26, mitochondrial OS=Mus musculus GN=Mrps26 PE=2 SV=1	sp|Q80ZS3|RT26_MOUSE	23 kDa	4	0
T-complex protein 1 subunit alpha OS=Mus musculus GN=Tcp1 PE=1 SV=3	sp|P11983|TCPA_MOUSE	60 kDa	18	22
Isocitrate dehydrogenase [NAD] subunit alpha, mitochondrial OS=Mus musculus GN=	sp|Q9D6R2|IDH3A_MOUSE	40 kDa	19	21
60S ribosomal protein L23 OS=Homo sapiens GN=RPL23 PE=1 SV=1	sp|P62829|RL23_HUMAN (+1)	15 kDa	19	19
40S ribosomal protein S19 OS=Mus musculus GN=Rps19 PE=1 SV=3	sp|Q9CZX8|RS19_MOUSE	16 kDa	20	20
Alpha-2-macroglobulin OS=Homo sapiens GN=A2M PE=1 SV=3	sp|P01023|A2MG_HUMAN	163 kDa	19	15
26S protease regulatory subunit 6A OS=Mus musculus GN=Psmc3 PE=2 SV=2	sp|O88685|PRS6A_MOUSE	50 kDa	24	14
Polyadenylate-binding protein 4 OS=Homo sapiens GN=PABPC4 PE=1 SV=1	sp|Q13310|PABP4_HUMAN	71 kDa	65	13
Probable ubiquitin carboxyl-terminal hydrolase FAF-X OS=Mus musculus GN=Usp9x P	sp|P70398|USP9X_MOUSE	291 kDa	19	12
Tripartite motif-containing protein 72 OS=Mus musculus GN=Trim72 PE=1 SV=1	sp|Q1XH17|TRI72_MOUSE	53 kDa	13	26
Heterogeneous nuclear ribonucleoproteins A2/B1 OS=Mus musculus GN=Hnrnpa2b1	sp|O88569|ROA2_MOUSE (+1)	37 kDa	25	13
Asparaginyl-tRNA synthetase, cytoplasmic OS=Mus musculus GN=Nars PE=1 SV=2	sp|Q8BP47|SYNC_MOUSE	64 kDa	24	15
Tropomodulin-1 OS=Mus musculus GN=Tmod1 PE=2 SV=2	sp|P49813|TMOD1_MOUSE	40 kDa	0	39
Stromal cell-derived factor 2 OS=Mus musculus GN=Sdf2 PE=2 SV=1	sp|Q9DCT5|SDF2_MOUSE	23 kDa	20	17
Kelch repeat and BTB domain-containing protein 5 OS=Mus musculus GN=Kbtbd5 PE:	sp|Q9D783|KBTB5_MOUSE	70 kDa	19	18
40S ribosomal protein S15 OS=Homo sapiens GN=RPS15 PE=1 SV=2	sp|P62841|RS15_HUMAN (+1)	17 kDa	25	13
Heat shock protein 75 kDa, mitochondrial OS=Mus musculus GN=Trap1 PE=1 SV=1	sp|Q9CQN1|TRAP1_MOUSE	80 kDa	22	18
T-complex protein 1 subunit theta OS=Mus musculus GN=Cct8 PE=1 SV=3	sp|P42932|TCPQ_MOUSE	60 kDa	13	25
60S ribosomal protein L30 OS=Homo sapiens GN=RPL30 PE=1 SV=2	sp|P62888|RL30_HUMAN (+1)	13 kDa	21	17
Malate dehydrogenase, mitochondrial OS=Mus musculus GN=Mdh2 PE=1 SV=3	sp|P08249|MDHM_MOUSE	36 kDa	8	28
14-3-3 protein zeta/delta OS=Mus musculus GN=Ywhaz PE=1 SV=1	sp|P63101|1433Z_MOUSE (+1)	28 kDa	14	27
40S ribosomal protein S15a OS=Homo sapiens GN=RPS15A PE=1 SV=2	sp|P62244|RS15A_HUMAN (+1)	15 kDa	21	16
Junction plakoglobin OS=Homo sapiens GN=JUP PE=1 SV=3	sp|P14923|PLAK_HUMAN (+1)	82 kDa	8	28
Calpain-2 catalytic subunit OS=Mus musculus GN=Capn2 PE=2 SV=4	sp|O08529|CAN2_MOUSE	80 kDa	24	12
Ig heavy chain V region 5-76 OS=Mus musculus PE=1 SV=1	sp|P18529|HVM58_MOUSE	13 kDa	7	9
Dihydrolipoyl dehydrogenase, mitochondrial OS=Mus musculus GN=Dld PE=1 SV=2	sp|O08749|DLDH_MOUSE	54 kDa	16	17
60S ribosomal protein L24 OS=Homo sapiens GN=RPL24 PE=1 SV=1	sp|P83731|RL24_HUMAN (+1)	18 kDa	30	5
Alpha-actinin-1 OS=Mus musculus GN=Actn1 PE=1 SV=1	sp|Q7TPR4|ACTN1_MOUSE	103 kDa	15	42
40S ribosomal protein S18 OS=Homo sapiens GN=RPS18 PE=1 SV=3	sp|P62269|RS18_HUMAN (+1)	18 kDa	23	12
ADP/ATP translocase 1 OS=Mus musculus GN=Slc25a4 PE=1 SV=4	sp|P48962|ADT1_MOUSE	33 kDa	0	76
Hemoglobin subunit alpha OS=Mus musculus GN=Hba PE=1 SV=2	sp|P01942|HBA_MOUSE	15 kDa	14	17
T-complex protein 1 subunit beta OS=Mus musculus GN=Cct2 PE=1 SV=4	sp|P80314|TCPB_MOUSE	57 kDa	14	20
Myosin light polypeptide 6 OS=Homo sapiens GN=MYL6 PE=1 SV=2	sp|P60660|MYL6_HUMAN (+1)	17 kDa	21	55
60S ribosomal protein L28 OS=Mus musculus GN=Rpl28 PE=1 SV=2	sp|P41105|RL28_MOUSE	16 kDa	26	8
Annexin A2 OS=Mus musculus GN=Anxa2 PE=1 SV=2	sp|P07356|ANXA2_MOUSE	39 kDa	7	28
40S ribosomal protein S20 OS=Homo sapiens GN=RPS20 PE=1 SV=1	sp|P60866|RS20_HUMAN (+1)	13 kDa	21	13
Tubulin alpha-1A chain OS=Mus musculus GN=Tuba1a PE=1 SV=1	sp|P68369|TBA1A_MOUSE (+1)	50 kDa	131	95
ATP synthase subunit gamma, mitochondrial OS=Mus musculus GN=Atp5c1 PE=1 SV=	sp|Q91VR2|ATPG_MOUSE	33 kDa	15	18
Aldehyde dehydrogenase, mitochondrial OS=Mus musculus GN=Aldh2 PE=1 SV=1	sp|P47738|ALDH2_MOUSE	57 kDa	16	14
Serine/threonine-protein phosphatase 2A 65 kDa regulatory subunit A alpha isoform	sp|P30153|2AAA_HUMAN (+1)	65 kDa	16	15

2-oxoglutarate dehydrogenase, mitochondrial OS=Mus musculus GN=Ogdh PE=1 SV=	sp|Q60597|ODO1_MOUSE	116 kDa	14	19
ATP-dependent RNA helicase DDX1 OS=Mus musculus GN=Ddx1 PE=1 SV=1	sp|Q91VR5|DDX1_MOUSE	83 kDa	21	10
Creatine kinase M-type OS=Mus musculus GN=Ckm PE=1 SV=1	sp|P07310|KCRM_MOUSE	43 kDa	0	31
Importin subunit beta-1 OS=Mus musculus GN=Kpnb1 PE=1 SV=2	sp|P70168|IMB1_MOUSE	97 kDa	14	14
Mitochondrial import inner membrane translocase subunit TIM50 OS=Mus musculus	sp|Q9D880|TIM50_MOUSE	40 kDa	14	17
Troponin I, slow skeletal muscle OS=Mus musculus GN=Tnni1 PE=2 SV=3	sp|Q9WUZ5|TNNI1_MOUSE	22 kDa	16	21
Cofilin-1 OS=Mus musculus GN=Cfl1 PE=1 SV=3	sp|P18760|COF1_MOUSE	19 kDa	13	17
Succinyl-CoA ligase [GDP-forming] subunit alpha, mitochondrial OS=Mus musculus GI	sp|Q9WUM5|SUCA_MOUSE	36 kDa	21	9
Ubiquitin-like modifier-activating enzyme 1 OS=Mus musculus GN=Uba1 PE=1 SV=1	sp|Q02053|UBA1_MOUSE	118 kDa	7	24
Lysyl-tRNA synthetase OS=Mus musculus GN=Kars PE=1 SV=1	sp|Q99MN1|SYK_MOUSE	68 kDa	18	12
Phosphoserine aminotransferase OS=Mus musculus GN=Psat1 PE=1 SV=1	sp|Q99K85|SERC_MOUSE	40 kDa	13	18
Glycyl-tRNA synthetase OS=Mus musculus GN=Gars PE=1 SV=1	sp|Q9CZD3|SYG_MOUSE	82 kDa	14	17
Myosin-14 OS=Homo sapiens GN=MYH14 PE=1 SV=1	sp|Q7Z406|MYH14_HUMAN	228 kDa	95	164
Serum albumin OS=Mus musculus GN=Alb PE=1 SV=3	sp|P07724|ALBU_MOUSE	69 kDa	9	19
Argininosuccinate synthase OS=Mus musculus GN=Ass1 PE=1 SV=1	sp|P16460|ASSY_MOUSE	47 kDa	8	22
T-complex protein 1 subunit gamma OS=Mus musculus GN=Cct3 PE=1 SV=1	sp|P80318|TCPG_MOUSE	61 kDa	14	15
Dihydrolipoyllysine-residue succinyltransferase component of 2-oxoglutarate dehydr	sp|Q9D2G2|ODO2_MOUSE	49 kDa	16	10
Ubiquitin thioesterase OTUB1 OS=Mus musculus GN=Otub1 PE=1 SV=2	sp|Q7TQI3|OTUB1_MOUSE (+1)	31 kDa	4	0
Neuroblast differentiation-associated protein AHNAK OS=Homo sapiens GN=AHNAK I	sp|Q09666|AHNK_HUMAN	629 kDa	4	17
Annexin A7 OS=Mus musculus GN=Anxa7 PE=2 SV=2	sp|Q07076|ANXA7_MOUSE	50 kDa	7	19
26S protease regulatory subunit 8 OS=Homo sapiens GN=PSMC5 PE=1 SV=1	sp|P62195|PRS8_HUMAN (+1)	46 kDa	19	9
40S ribosomal protein S12 OS=Homo sapiens GN=RPS12 PE=1 SV=3	sp|P25398|RS12_HUMAN	15 kDa	21	7
Protein unc-45 homolog B OS=Mus musculus GN=Unc45b PE=1 SV=1	sp|Q8CGY6|UN45B_MOUSE	104 kDa	6	21
Nucleoside diphosphate kinase A OS=Mus musculus GN=Nme1 PE=1 SV=1	sp|P15532|NDKA_MOUSE	17 kDa	10	19
T-complex protein 1 subunit delta OS=Mus musculus GN=Cct4 PE=1 SV=3	sp|P80315|TCPD_MOUSE	58 kDa	11	21
Dolichyl-diphosphooligosaccharide--protein glycosyltransferase subunit STT3A OS=M	sp|P46978|STT3A_MOUSE	81 kDa	16	11
26S protease regulatory subunit 6B OS=Homo sapiens GN=PSMC4 PE=1 SV=2	sp|P43686|PRS6B_HUMAN (+1)	47 kDa	21	9
Heterogeneous nuclear ribonucleoprotein A3 OS=Homo sapiens GN=HNRNPA3 PE=1	sp|P51991|ROA3_HUMAN (+1)	40 kDa	22	7
40S ribosomal protein S10 OS=Mus musculus GN=Rps10 PE=1 SV=1	sp|P63325|RS10_MOUSE	19 kDa	15	14
Aldose reductase OS=Mus musculus GN=Akr1b1 PE=1 SV=3	sp|P45376|ALDR_MOUSE	36 kDa	7	20
Dihydropyrimidinase-related protein 3 OS=Mus musculus GN=Dpysl3 PE=1 SV=1	sp|Q62188|DPYL3_MOUSE	62 kDa	18	10
60S ribosomal protein L11 OS=Homo sapiens GN=RPL11 PE=1 SV=2	sp|P62913|RL11_HUMAN (+1)	20 kDa	21	7
Heat shock 70 kDa protein 4 OS=Mus musculus GN=Hspa4 PE=1 SV=1	sp|Q61316|HSP74_MOUSE	94 kDa	0	26
40S ribosomal protein S17 OS=Homo sapiens GN=RPS17 PE=1 SV=2	sp|P08708|RS17_HUMAN (+1)	16 kDa	18	11
Cullin-5 OS=Mus musculus GN=Cul5 PE=1 SV=3	sp|Q9D5V5|CUL5_MOUSE	91 kDa	0	28
Heterogeneous nuclear ribonucleoprotein H OS=Mus musculus GN=Hnrnph1 PE=1 SV	sp|O35737|HNRH1_MOUSE	49 kDa	57	38
Histone-arginine methyltransferase CARM1 OS=Homo sapiens GN=CARM1 PE=1 SV=3	sp|Q86X55|CARM1_HUMAN (+1)	66 kDa	22	4
TAR DNA-binding protein 43 OS=Mus musculus GN=Tardbp PE=1 SV=1	sp|Q921F2|TADBP_MOUSE	45 kDa	19	9
Troponin T, cardiac muscle OS=Mus musculus GN=Tnnt2 PE=2 SV=2	sp|P50752|TNNT2_MOUSE	36 kDa	4	26
5'-AMP-activated protein kinase subunit gamma-1 OS=Mus musculus GN=Prkag1 PE=	sp|O54950|AAKG1_MOUSE	38 kDa	4	0
CDGSH iron-sulfur domain-containing protein 3, mitochondrial OS=Mus musculus GN	sp|B1AR13|CISD3_MOUSE	16 kDa	12	15
60S ribosomal protein L22 OS=Homo sapiens GN=RPL22 PE=1 SV=2	sp|P35268|RL22_HUMAN (+1)	15 kDa	17	10
Profilin-1 OS=Mus musculus GN=Pfn1 PE=1 SV=2	sp|P62962|PROF1_MOUSE	15 kDa	9	18
Tubulin beta-2C chain OS=Homo sapiens GN=TUBB2C PE=1 SV=1	sp|P68371|TBB2C_HUMAN (+1)	50 kDa	161	122
Phospholipase A-2-activating protein OS=Mus musculus GN=Plaa PE=2 SV=4	sp|P27612|PLAP_MOUSE	87 kDa	14	12
Heterogeneous nuclear ribonucleoprotein Q OS=Mus musculus GN=Syncrip PE=1 SV=	sp|Q7TMK9|HNRPQ_MOUSE	70 kDa	23	3
40S ribosomal protein S23 OS=Homo sapiens GN=RPS23 PE=1 SV=3	sp|P62266|RS23_HUMAN (+1)	16 kDa	18	9
Small nuclear ribonucleoprotein G-like protein OS=Homo sapiens PE=3 SV=2	sp|A8MWD9|RUXGL_HUMAN (+2)	9 kDa	4	0
Acetyl-CoA carboxylase 1 OS=Mus musculus GN=Acaca PE=1 SV=1	sp|Q5SWU9|ACACA_MOUSE	265 kDa	0	24
Long-chain specific acyl-CoA dehydrogenase, mitochondrial OS=Mus musculus GN=Ac	sp|P51174|ACADL_MOUSE	48 kDa	12	14
Transcription factor A, mitochondrial OS=Mus musculus GN=Tfam PE=1 SV=2	sp|P40630|TFAM_MOUSE	28 kDa	19	7
Actin-related protein 2/3 complex subunit 4 OS=Homo sapiens GN=ARPC4 PE=1 SV=3	sp|P59998|ARPC4_HUMAN (+1)	20 kDa	9	18
Protein transport protein Sec23A OS=Mus musculus GN=Sec23a PE=1 SV=2	sp|Q01405|SC23A_MOUSE	86 kDa	17	10
Keratin, type I cytoskeletal 18 OS=Homo sapiens GN=KRT18 PE=1 SV=2	sp|P05783|K1C18_HUMAN	48 kDa	16	12
Heterogeneous nuclear ribonucleoprotein D0 OS=Homo sapiens GN=HNRNPD PE=1 S	sp|Q14103|HNRPD_HUMAN (+1)	38 kDa	14	12
Protein-glutamine gamma-glutamyltransferase E OS=Homo sapiens GN=TGM3 PE=1 S	sp|Q08188|TGM3_HUMAN	77 kDa	4	0
Puromycin-sensitive aminopeptidase OS=Mus musculus GN=Npepps PE=1 SV=2	sp|Q11011|PSA_MOUSE	103 kDa	14	11
Phosphoglycerate kinase 1 OS=Mus musculus GN=Pgk1 PE=1 SV=4	sp|P09411|PGK1_MOUSE	45 kDa	4	22
GTP-binding nuclear protein Ran OS=Homo sapiens GN=RAN PE=1 SV=3	sp|P62826|RAN_HUMAN (+1)	24 kDa	17	7
60S ribosomal protein L23a OS=Homo sapiens GN=RPL23A PE=1 SV=1	sp|P62750|RL23A_HUMAN (+1)	18 kDa	19	5
Serine hydroxymethyltransferase, mitochondrial OS=Homo sapiens GN=SHMT2 PE=1	sp|P34897|GLYM_HUMAN	56 kDa	10	15
Endoplasmic reticulum-Golgi intermediate compartment protein 1 OS=Mus musculus	sp|Q9DC16|ERGI1_MOUSE	33 kDa	9	16
DDB1- and CUL4-associated factor 8 OS=Mus musculus GN=Dcaf8 PE=1 SV=1	sp|Q8N7N5|DCAF8_MOUSE	66 kDa	16	8
Isocitrate dehydrogenase [NAD] subunit beta, mitochondrial OS=Homo sapiens GN=I	sp|O43837|IDH3B_HUMAN	42 kDa	12	13
Transcription elongation factor B polypeptide 2 OS=Mus musculus GN=Tceb2 PE=1 S\	sp|P62869|ELOB_MOUSE	13 kDa	11	14
Single-stranded DNA-binding protein, mitochondrial OS=Mus musculus GN=Ssbp1 PE	sp|Q9CYR0|SSBP_MOUSE	17 kDa	9	16
Succinyl-CoA ligase [ADP-forming] subunit beta, mitochondrial OS=Mus musculus GN	sp|Q9Z2I9|SUCB1_MOUSE	50 kDa	11	14
Leiomodin-3 OS=Homo sapiens GN=LMOD3 PE=2 SV=1	sp|Q0VAK6|LMOD3_HUMAN	65 kDa	12	11
Protein arginine N-methyltransferase 1 OS=Homo sapiens GN=PRMT1 PE=1 SV=2	sp|Q99873|ANM1_HUMAN (+1)	42 kDa	16	7
Alpha-actinin-3 OS=Mus musculus GN=Actn3 PE=2 SV=1	sp|O88990|ACTN3_MOUSE	103 kDa	9	32
Leiomodin-2 OS=Mus musculus GN=Lmod2 PE=1 SV=1	sp|Q3UHZ5|LMOD2_MOUSE	62 kDa	3	20
Nuclease-sensitive element-binding protein 1 OS=Mus musculus GN=Ybx1 PE=1 SV=3	sp|P62960|YBOX1_MOUSE	36 kDa	37	10
DnaJ homolog subfamily A member 3, mitochondrial OS=Mus musculus GN=Dnaja3 F	sp|Q99M87|DNJA3_MOUSE	52 kDa	12	12
Dihydrolipoyllysine-residue acetyltransferase component of pyruvate dehydrogenase	sp|Q8BMF4|ODP2_MOUSE	68 kDa	7	16
Coronin-6 OS=Mus musculus GN=Coro6 PE=2 SV=1	sp|Q920M5|CORO6_MOUSE	53 kDa	0	23
Heterogeneous nuclear ribonucleoprotein A1 OS=Homo sapiens GN=HNRNPA1 PE=1	sp|P09651|ROA1_HUMAN (+1)	39 kDa	22	10
Calcium-binding mitochondrial carrier protein Aralar1 OS=Homo sapiens GN=SLC25A	sp|O75746|CMC1_HUMAN (+1)	75 kDa	0	24

Translationally-controlled tumor protein OS=Mus musculus GN=Tpt1 PE=1 SV=1	sp|P63028|TCTP_MOUSE	19 kDa	10	14
Transcription elongation factor B polypeptide 1 OS=Mus musculus GN=Tceb1 PE=1 S\	sp|P83940|ELOC_MOUSE (+1)	12 kDa	13	11
Cullin-associated NEDD8-dissociated protein 1 OS=Mus musculus GN=Cand1 PE=2 SV	sp|Q6ZQ38|CAND1_MOUSE	136 kDa	15	9
Ribonuclease inhibitor OS=Mus musculus GN=Rnh1 PE=1 SV=1	sp|Q91VI7|RINI_MOUSE	50 kDa	17	7
ATP synthase subunit d, mitochondrial OS=Mus musculus GN=Atp5h PE=1 SV=3	sp|Q9DCX2|ATP5H_MOUSE	19 kDa	7	17
Phosphoenolpyruvate carboxykinase [GTP], mitochondrial OS=Mus musculus GN=Pck	sp|Q8BH04|PCKGM_MOUSE	71 kDa	22	2
Glucose-6-phosphate isomerase OS=Mus musculus GN=Gpi PE=1 SV=4	sp|P06745|G6PI_MOUSE	63 kDa	0	24
Protein disulfide-isomerase A3 OS=Mus musculus GN=Pdia3 PE=1 SV=2	sp|P27773|PDIA3_MOUSE	57 kDa	0	24
Lamin-B2 OS=Mus musculus GN=Lmnb2 PE=1 SV=2	sp|P21619|LMNB2_MOUSE (+1)	67 kDa	4	0
Trans-2,3-enoyl-CoA reductase OS=Mus musculus GN=Tecr PE=1 SV=1	sp|Q9CY27|TECR_MOUSE	36 kDa	18	3
40S ribosomal protein SA OS=Homo sapiens GN=RPSA PE=1 SV=4	sp|P08865|RSSA_HUMAN (+1)	33 kDa	16	7
Golgi apparatus protein 1 OS=Mus musculus GN=Glg1 PE=1 SV=1	sp|Q61543|GSLG1_MOUSE (+1)	134 kDa	3	0
60S ribosomal protein L9 OS=Mus musculus GN=Rpl9 PE=2 SV=2	sp|P51410|RL9_MOUSE	22 kDa	21	2
Citrate synthase, mitochondrial OS=Mus musculus GN=Cs PE=1 SV=1	sp|Q9CZU6|CISY_MOUSE	52 kDa	2	20
Ras suppressor protein 1 OS=Mus musculus GN=Rsu1 PE=2 SV=3	sp|Q01730|RSU1_MOUSE	32 kDa	13	9
Annexin A1 OS=Mus musculus GN=Anxa1 PE=1 SV=2	sp|P10107|ANXA1_MOUSE	39 kDa	5	20
NADH-cytochrome b5 reductase 3 OS=Mus musculus GN=Cyb5r3 PE=1 SV=3	sp|Q9DCN2|NB5R3_MOUSE	34 kDa	9	13
Threonyl-tRNA synthetase, cytoplasmic OS=Mus musculus GN=Tars PE=1 SV=2	sp|Q9D0R2|SYTC_MOUSE	83 kDa	2	20
Vacuolar protein sorting-associated protein 28 homolog OS=Mus musculus GN=Vps2ℓ	sp|Q9D1C8|VPS28_MOUSE (+1)	25 kDa	3	0
Cytoplasmic dynein 1 intermediate chain 2 OS=Mus musculus GN=Dync1i2 PE=2 SV=1	sp|O88487|DC1I2_MOUSE	68 kDa	0	23
Plectin OS=Homo sapiens GN=PLEC PE=1 SV=3	sp|Q15149|PLEC_HUMAN	532 kDa	307	608
Nucleosome assembly protein 1-like 1 OS=Mus musculus GN=Nap1l1 PE=1 SV=2	sp|P28656|NP1L1_MOUSE	45 kDa	14	7
Surfeit locus protein 4 OS=Homo sapiens GN=SURF4 PE=1 SV=3	sp|O15260|SURF4_HUMAN (+1)	30 kDa	7	11
Dolichyl-diphosphooligosaccharide--protein glycosyltransferase 48 kDa subunit OS=M	sp|O54734|OST48_MOUSE	49 kDa	6	13
Succinate dehydrogenase [ubiquinone] flavoprotein subunit, mitochondrial OS=Mus	sp|Q8K2B3|DHSA_MOUSE	73 kDa	5	17
F-actin-capping protein subunit beta OS=Mus musculus GN=Capzb PE=1 SV=3	sp|P47757|CAPZB_MOUSE	31 kDa	10	12
Extended synaptotagmin-1 OS=Mus musculus GN=Esyt1 PE=2 SV=2	sp|Q3U7R1|ESYT1_MOUSE	122 kDa	6	15
Tripartite motif-containing protein 55 OS=Homo sapiens GN=TRIM55 PE=1 SV=2	sp|Q9BYV6|TRI55_HUMAN	60 kDa	0	31
Aldehyde dehydrogenase family 1 member L2, mitochondrial OS=Mus musculus GN=	sp|Q8K009|AL1L2_MOUSE	102 kDa	12	10
Prolyl 4-hydroxylase subunit alpha-1 OS=Mus musculus GN=P4ha1 PE=2 SV=2	sp|Q60715|P4HA1_MOUSE	61 kDa	17	5
Cytoskeleton-associated protein 4 OS=Mus musculus GN=Ckap4 PE=2 SV=2	sp|Q8BMK4|CKAP4_MOUSE	64 kDa	2	20
Aspartate aminotransferase, mitochondrial OS=Mus musculus GN=Got2 PE=1 SV=1	sp|P05202|AATM_MOUSE	47 kDa	0	20
Sodium/potassium-transporting ATPase subunit alpha-1 OS=Mus musculus GN=Atp1a	sp|Q8VDN2|AT1A1_MOUSE	113 kDa	9	8
Serine/threonine-protein phosphatase PGAM5, mitochondrial OS=Mus musculus GN	sp|Q8BX10|PGAM5_MOUSE	32 kDa	16	5
40S ribosomal protein S27 OS=Homo sapiens GN=RPS27 PE=1 SV=3	sp|P42677|RS27_HUMAN (+1)	9 kDa	11	8
Pyruvate dehydrogenase E1 component subunit beta, mitochondrial OS=Mus muscul	sp|Q9D051|ODPB_MOUSE	39 kDa	11	9
26S protease regulatory subunit 7 OS=Homo sapiens GN=PSMC2 PE=1 SV=3	sp|P35998|PRS7_HUMAN (+1)	49 kDa	16	6
Keratinocyte proline-rich protein OS=Homo sapiens GN=KPRP PE=1 SV=1	sp|Q5T749|KPRP_HUMAN	64 kDa	7	12
Replication protein A 70 kDa DNA-binding subunit OS=Mus musculus GN=Rpa1 PE=2	sp|Q8VEE4|RFA1_MOUSE	69 kDa	4	17
40S ribosomal protein S5 OS=Mus musculus GN=Rps5 PE=2 SV=3	sp|P97461|RS5_MOUSE	23 kDa	10	10
60S ribosomal protein L37a OS=Homo sapiens GN=RPL37A PE=1 SV=2	sp|P61513|RL37A_HUMAN (+1)	10 kDa	12	9
Activator of 90 kDa heat shock protein ATPase homolog 1 OS=Mus musculus GN=Ahs	sp|Q8BK64|AHSA1_MOUSE	38 kDa	13	7
Putative pre-mRNA-splicing factor ATP-dependent RNA helicase DHX15 OS=Mus mus	sp|O35286|DHX15_MOUSE (+1)	91 kDa	16	3
HCLS1-associated protein X-1 OS=Mus musculus GN=Hax1 PE=1 SV=1	sp|O35387|HAX1_MOUSE	32 kDa	3	0
Glyceraldehyde-3-phosphate dehydrogenase OS=Homo sapiens GN=GAPDH PE=1 SV=	sp|P04406|G3P_HUMAN	36 kDa	122	26
Ras-related protein Rab-1A OS=Homo sapiens GN=RAB1A PE=1 SV=3	sp|P62820|RAB1A_HUMAN (+1)	23 kDa	11	7
ER lumen protein retaining receptor 1 OS=Homo sapiens GN=KDELR1 PE=1 SV=1	sp|P24390|ERD21_HUMAN (+3)	25 kDa	4	6
cAMP-dependent protein kinase catalytic subunit alpha OS=Mus musculus GN=Prkac	sp|P05132|KAPCA_MOUSE	41 kDa	7	13
Tropomodulin-3 OS=Mus musculus GN=Tmod3 PE=1 SV=1	sp|Q9JHJ0|TMOD3_MOUSE	40 kDa	0	21
Tubulin alpha-4A chain OS=Homo sapiens GN=TUBA4A PE=1 SV=1	sp|P68366|TBA4A_HUMAN (+1)	50 kDa	141	89
Poly(rC)-binding protein 2 OS=Homo sapiens GN=PCBP2 PE=1 SV=1	sp|Q15366|PCBP2_HUMAN (+1)	39 kDa	20	8
Calpain small subunit 1 OS=Mus musculus GN=Capns1 PE=2 SV=1	sp|O88456|CPNS1_MOUSE	28 kDa	12	6
Galectin-3 OS=Mus musculus GN=Lgals3 PE=1 SV=3	sp|P16110|LEG3_MOUSE	28 kDa	8	11
Adenosylhomocysteinase OS=Mus musculus GN=Ahcy PE=1 SV=3	sp|P50247|SAHH_MOUSE	48 kDa	3	15
26S protease regulatory subunit 4 OS=Homo sapiens GN=PSMC1 PE=1 SV=1	sp|P62191|PRS4_HUMAN (+1)	49 kDa	12	7
Hexokinase-1 OS=Mus musculus GN=Hk1 PE=1 SV=3	sp|P17710|HXK1_MOUSE	108 kDa	11	19
Arginyl-tRNA synthetase, cytoplasmic OS=Mus musculus GN=Rars PE=2 SV=2	sp|Q9D0I9|SYRC_MOUSE	76 kDa	11	8
14-3-3 protein theta OS=Mus musculus GN=Ywhaq PE=1 SV=1	sp|P68254|1433T_MOUSE	28 kDa	17	17
Actin, alpha cardiac muscle 1 OS=Homo sapiens GN=ACTC1 PE=1 SV=1	sp|P68032|ACTC_HUMAN (+1)	42 kDa	72	168
Elongation factor 1-gamma OS=Homo sapiens GN=EEF1G PE=1 SV=3	sp|P26641|EF1G_HUMAN	50 kDa	10	8
Nephronectin OS=Homo sapiens GN=NPNT PE=2 SV=3	sp|Q6UXI9|NPNT_HUMAN (+1)	62 kDa	3	0
Dynein light chain 1, cytoplasmic OS=Homo sapiens GN=DYNLL1 PE=1 SV=1	sp|P63167|DYL1_HUMAN (+1)	10 kDa	12	8
Calreticulin OS=Mus musculus GN=Calr PE=1 SV=1	sp|P14211|CALR_MOUSE	48 kDa	0	19
3-beta-hydroxysteroid-Delta(8),Delta(7)-isomerase OS=Mus musculus GN=Ebp PE=1 S	sp|P70245|EBP_MOUSE	26 kDa	12	4
Myosin-1 OS=Homo sapiens GN=MYH1 PE=1 SV=3	sp|P12882|MYH1_HUMAN	223 kDa	0	826
Dolichyl-diphosphooligosaccharide--protein glycosyltransferase subunit 2 OS=Mus m	sp|Q9DBG6|RPN2_MOUSE	69 kDa	0	14
Actin, cytoplasmic 1 OS=Homo sapiens GN=ACTB PE=1 SV=1	sp|P60709|ACTB_HUMAN (+1)	42 kDa	122	177
KH domain-containing, RNA-binding, signal transduction-associated protein 1 OS=Ho	sp|Q07666|KHDR1_HUMAN (+1)	48 kDa	12	4
Enoyl-CoA delta isomerase 1, mitochondrial OS=Mus musculus GN=Eci1 PE=2 SV=1	sp|P42125|ECI1_MOUSE	32 kDa	14	3
Polypyrimidine tract-binding protein 1 OS=Mus musculus GN=Ptbp1 PE=1 SV=2	sp|P17225|PTBP1_MOUSE	56 kDa	10	6
RNA binding protein fox-1 homolog 2 OS=Homo sapiens GN=RBFOX2 PE=1 SV=3	sp|O43251|RFOX2_HUMAN (+3)	41 kDa	12	6
tRNA-splicing ligase RtcB homolog OS=Mus musculus GN=D10Wsu52e PE=2 SV=1	sp|Q99LF4|RTCB_MOUSE	55 kDa	9	9
Interleukin enhancer-binding factor 2 OS=Homo sapiens GN=ILF2 PE=1 SV=2	sp|Q12905|ILF2_HUMAN (+1)	43 kDa	11	7
Vacuolar protein sorting-associated protein 29 OS=Homo sapiens GN=VPS29 PE=1 SV	sp|Q9UBQ0|VPS29_HUMAN	21 kDa	13	5
Delta-1-pyrroline-5-carboxylate synthase OS=Mus musculus GN=Aldh18a1 PE=2 SV=2	sp|Q9Z110|P5CS_MOUSE	87 kDa	7	10
Poly [ADP-ribose] polymerase 1 OS=Homo sapiens GN=PARP1 PE=1 SV=4	sp|P09874|PARP1_HUMAN	113 kDa	12	6
Pyruvate carboxylase, mitochondrial OS=Mus musculus GN=Pc PE=1 SV=1	sp|Q05920|PYC_MOUSE	130 kDa	12	6

Protein disulfide-isomerase A6 OS=Mus musculus GN=Pdia6 PE=1 SV=3	sp|Q922R8|PDIA6_MOUSE	48 kDa	2	16
Thimet oligopeptidase OS=Mus musculus GN=Thop1 PE=1 SV=1	sp|Q8C1A5|THOP1_MOUSE	78 kDa	0	18
Sarcoplasmic/endoplasmic reticulum calcium ATPase 1 OS=Homo sapiens GN=ATP2A	sp|O14983|AT2A1_HUMAN	110 kDa	83	123
Troponin C, slow skeletal and cardiac muscles OS=Mus musculus GN=Tnnc1 PE=2 SV=	sp|P19123|TNNC1_MOUSE (+1)	18 kDa	6	11
Reticulocalbin-2 OS=Mus musculus GN=Rcn2 PE=2 SV=1	sp|Q8BP92|RCN2_MOUSE	37 kDa	13	4
Polymerase delta-interacting protein 2 OS=Mus musculus GN=Poldip2 PE=2 SV=1	sp|Q91VA6|PDIP2_MOUSE (+1)	42 kDa	9	6
Heterogeneous nuclear ribonucleoprotein L OS=Mus musculus GN=Hnrnpl PE=1 SV=2	sp|Q8R081|HNRPL_MOUSE	64 kDa	12	7
Acyl-coenzyme A thioesterase 9, mitochondrial OS=Mus musculus GN=Acot9 PE=1 SV	sp|Q9R0X4|ACOT9_MOUSE	51 kDa	7	10
60S ribosomal protein L31 OS=Homo sapiens GN=RPL31 PE=1 SV=1	sp|P62899|RL31_HUMAN (+1)	14 kDa	13	4
Isocitrate dehydrogenase [NADP], mitochondrial OS=Mus musculus GN=Idh2 PE=1 SV	sp|P54071|IDHP_MOUSE	51 kDa	0	17
Myosin-4 OS=Homo sapiens GN=MYH4 PE=1 SV=2	sp|Q9Y623|MYH4_HUMAN	223 kDa	132	387
Glutathione S-transferase P 1 OS=Mus musculus GN=Gstp1 PE=1 SV=2	sp|P19157|GSTP1_MOUSE	24 kDa	7	7
Nodal modulator 1 OS=Mus musculus GN=Nomo1 PE=1 SV=1	sp|Q6GQT9|NOMO1_MOUSE	133 kDa	8	6
Electron transfer flavoprotein subunit alpha, mitochondrial OS=Mus musculus GN=Et	sp|Q99LC5|ETFA_MOUSE	35 kDa	7	7
1-acyl-sn-glycerol-3-phosphate acyltransferase gamma OS=Mus musculus GN=Agpat3	sp|Q9D517|PLCC_MOUSE	43 kDa	6	9
Alpha-2-HS-glycoprotein OS=Homo sapiens GN=AHSG PE=1 SV=1	sp|P02765|FETUA_HUMAN	39 kDa	5	10
Coatomer subunit delta OS=Mus musculus GN=Arcn1 PE=2 SV=2	sp|Q5XJY5|COPD_MOUSE	57 kDa	5	9
60S ribosomal protein L34 OS=Homo sapiens GN=RPL34 PE=1 SV=3	sp|P49207|RL34_HUMAN (+1)	13 kDa	11	3
DNA topoisomerase 2-beta OS=Homo sapiens GN=TOP2B PE=1 SV=3	sp|Q02880|TOP2B_HUMAN (+1)	183 kDa	13	3
UDP-N-acetylglucosamine--peptide N-acetylglucosaminyltransferase 110 kDa subunit	sp|Q8CGY8|OGT1_MOUSE	117 kDa	11	5
Myosin-Ie OS=Homo sapiens GN=MYO1E PE=1 SV=2	sp|Q12965|MYO1E_HUMAN	127 kDa	7	9
F-actin-capping protein subunit alpha-2 OS=Mus musculus GN=Capza2 PE=1 SV=3	sp|P47754|CAZA2_MOUSE	33 kDa	7	9
6-phosphofructokinase type C OS=Mus musculus GN=Pfkp PE=1 SV=1	sp|Q9WUA3|K6PP_MOUSE	85 kDa	14	10
1-phosphatidylinositol-4,5-bisphosphate phosphodiesterase beta-4 OS=Homo sapien	sp|Q15147|PLCB4_HUMAN	134 kDa	5	2
Tyrosyl-tRNA synthetase, cytoplasmic OS=Mus musculus GN=Yars PE=2 SV=3	sp|Q91WQ3|SYYC_MOUSE	59 kDa	6	9
Tropomyosin alpha-3 chain OS=Mus musculus GN=Tpm3 PE=1 SV=2	sp|P21107|TPM3_MOUSE	33 kDa	0	72
S-adenosylmethionine synthase isoform type-2 OS=Homo sapiens GN=MAT2A PE=1 S	sp|P31153|METK2_HUMAN (+1)	44 kDa	7	9
Actin-related protein 2/3 complex subunit 3 OS=Mus musculus GN=Arpc3 PE=1 SV=3	sp|Q9JM76|ARPC3_MOUSE	21 kDa	8	8
Fascin OS=Mus musculus GN=Fscn1 PE=1 SV=4	sp|Q61553|FSCN1_MOUSE	55 kDa	2	14
Kelch-like protein 31 OS=Mus musculus GN=Klhl31 PE=1 SV=1	sp|Q8BWA5|KLH31_MOUSE	70 kDa	3	0
Target of EGR1 protein 1 OS=Mus musculus GN=Toe1 PE=2 SV=1	sp|Q9D2E2|TOE1_MOUSE	57 kDa	3	0
Creatine kinase B-type OS=Mus musculus GN=Ckb PE=1 SV=1	sp|Q04447|KCRB_MOUSE	43 kDa	0	18
Stress-induced-phosphoprotein 1 OS=Mus musculus GN=Stip1 PE=1 SV=1	sp|Q60864|STIP1_MOUSE	63 kDa	0	15
Histone H4 OS=Homo sapiens GN=HIST1H4A PE=1 SV=2	sp|P62805|H4_HUMAN (+1)	11 kDa	11	3
V-type proton ATPase 116 kDa subunit a isoform 1 OS=Homo sapiens GN=ATP6V0A1	sp|Q93050|VPP1_HUMAN (+1)	96 kDa	0	12
Collagen alpha-1(V) chain OS=Mus musculus GN=Col5a1 PE=2 SV=2	sp|O88207|CO5A1_MOUSE	184 kDa	4	9
Histone H3.1 OS=Homo sapiens GN=HIST1H3A PE=1 SV=2	sp|P68431|H31_HUMAN (+6)	15 kDa	10	3
Eukaryotic translation initiation factor 5A-1 OS=Homo sapiens GN=EIF5A PE=1 SV=2	sp|P63241|IF5A1_HUMAN (+1)	17 kDa	6	9
Actin-related protein 2/3 complex subunit 5-like protein OS=Mus musculus GN=Arpc5	sp|Q9D898|ARP5L_MOUSE	17 kDa	6	9
Ras-related protein Rab-2A OS=Mus musculus GN=Rab2a PE=1 SV=1	sp|P53994|RAB2A_MOUSE (+1)	24 kDa	3	0
Adenylate kinase isoenzyme 1 OS=Mus musculus GN=Ak1 PE=1 SV=1	sp|Q9R0Y5|KAD1_MOUSE	22 kDa	5	9
Glutamate dehydrogenase 1, mitochondrial OS=Mus musculus GN=Glud1 PE=1 SV=1	sp|P26443|DHE3_MOUSE	61 kDa	0	13
RuvB-like 1 OS=Mus musculus GN=Ruvbl1 PE=1 SV=1	sp|P60122|RUVB1_MOUSE (+1)	50 kDa	10	4
Myosin-Va OS=Homo sapiens GN=MYO5A PE=1 SV=2	sp|Q9Y4I1|MYO5A_HUMAN	215 kDa	0	15
Troponin C, skeletal muscle OS=Mus musculus GN=Tnnc2 PE=1 SV=2	sp|P20801|TNNC2_MOUSE	18 kDa	4	11
28S ribosomal protein S6, mitochondrial OS=Mus musculus GN=Mrps6 PE=2 SV=3	sp|P58064|RT06_MOUSE	14 kDa	7	8
Transferrin receptor protein 1 OS=Mus musculus GN=Tfrc PE=1 SV=1	sp|Q62351|TFR1_MOUSE	86 kDa	5	11
Mitochondrial fission 1 protein OS=Mus musculus GN=Fis1 PE=1 SV=1	sp|Q9CQ92|FIS1_MOUSE	17 kDa	6	9
Leukocyte elastase inhibitor A OS=Mus musculus GN=Serpinb1a PE=1 SV=1	sp|Q9D154|ILEUA_MOUSE	43 kDa	3	12
E3 ubiquitin-protein ligase RBX1 OS=Homo sapiens GN=RBX1 PE=1 SV=1	sp|P62877|RBX1_HUMAN (+1)	12 kDa	5	10
Seryl-tRNA synthetase, cytoplasmic OS=Mus musculus GN=Sars PE=2 SV=3	sp|P26638|SYSC_MOUSE	58 kDa	2	13
ATPase family AAA domain-containing protein 3 OS=Mus musculus GN=Atad3 PE=1 S	sp|Q925I1|ATAD3_MOUSE	67 kDa	0	14
ADP-ribosylation factor-like protein 1 OS=Homo sapiens GN=ARL1 PE=1 SV=1	sp|P40616|ARL1_HUMAN (+1)	20 kDa	7	2
Electron transfer flavoprotein subunit beta OS=Mus musculus GN=Etfb PE=1 SV=3	sp|Q9DCW4|ETFB_MOUSE	28 kDa	7	5
14-3-3 protein gamma OS=Homo sapiens GN=YWHAG PE=1 SV=2	sp|P61981|1433G_HUMAN (+1)	28 kDa	7	19
Elongation factor 1-gamma OS=Mus musculus GN=Eef1g PE=1 SV=3	sp|Q9D8N0|EF1G_MOUSE	50 kDa	9	18
Signal transducer and activator of transcription 3 OS=Homo sapiens GN=STAT3 PE=1	sp|P40763|STAT3_HUMAN (+1)	88 kDa	11	3
Adenylosuccinate synthetase isozyme 1 OS=Mus musculus GN=Adssl1 PE=1 SV=2	sp|P28650|PURA1_MOUSE	50 kDa	6	7
COMM domain-containing protein 2 OS=Mus musculus GN=Commd2 PE=1 SV=1	sp|Q8BXC6|COMD2_MOUSE	23 kDa	3	0
Phosphoglycerate mutase 2 OS=Mus musculus GN=Pgam2 PE=1 SV=3	sp|O70250|PGAM2_MOUSE	29 kDa	0	13
ATP-citrate synthase OS=Mus musculus GN=Acly PE=1 SV=1	sp|Q91V92|ACLY_MOUSE	120 kDa	7	5
Ribose-phosphate pyrophosphokinase 1 OS=Homo sapiens GN=PRPS1 PE=1 SV=2	sp|P60891|PRPS1_HUMAN (+1)	35 kDa	10	3
Heterogeneous nuclear ribonucleoproteins C1/C2 OS=Homo sapiens GN=HNRNPC PE	sp|P07910|HNRPC_HUMAN	34 kDa	7	7
4F2 cell-surface antigen heavy chain OS=Mus musculus GN=Slc3a2 PE=1 SV=1	sp|P10852|4F2_MOUSE	58 kDa	4	10
Proliferation-associated protein 2G4 OS=Mus musculus GN=Pa2g4 PE=1 SV=3	sp|P50580|PA2G4_MOUSE (+1)	44 kDa	6	8
60S ribosomal protein L38 OS=Homo sapiens GN=RPL38 PE=1 SV=2	sp|P63173|RL38_HUMAN (+1)	8 kDa	9	5
Eukaryotic translation initiation factor 3 subunit I OS=Homo sapiens GN=EIF3I PE=1 S	sp|Q13347|EIF3I_HUMAN	37 kDa	7	7
Glycerol-3-phosphate dehydrogenase 1-like protein OS=Mus musculus GN=Gpd1l PE=	sp|Q3ULJ0|GPD1L_MOUSE	38 kDa	10	4
Methionyl-tRNA synthetase, cytoplasmic OS=Mus musculus GN=Mars PE=2 SV=1	sp|Q68FL6|SYMC_MOUSE	101 kDa	7	7
NADH dehydrogenase [ubiquinone] iron-sulfur protein 2, mitochondrial OS=Mus mus	sp|Q91WD5|NDUS2_MOUSE	53 kDa	5	10
Eukaryotic translation initiation factor 2 subunit 2 OS=Mus musculus GN=Eif2s2 PE=1	sp|Q99L45|IF2B_MOUSE	38 kDa	7	7
cAMP-dependent protein kinase type I-alpha regulatory subunit OS=Mus musculus G	sp|Q9DBC7|KAP0_MOUSE	43 kDa	7	7
Serine protease HTRA2, mitochondrial OS=Mus musculus GN=Htra2 PE=1 SV=2	sp|Q9JIY5|HTRA2_MOUSE	49 kDa	8	6
Dynamin-2 OS=Mus musculus GN=Dnm2 PE=1 SV=2	sp|P39054|DYN2_MOUSE	98 kDa	4	12
Spliceosome RNA helicase BAT1 OS=Homo sapiens GN=BAT1 PE=1 SV=1	sp|Q13838|UAP56_HUMAN (+1)	49 kDa	5	9
WD repeat-containing protein 1 OS=Mus musculus GN=Wdr1 PE=1 SV=3	sp|O88342|WDR1_MOUSE	66 kDa	0	14
SRA stem-loop-interacting RNA-binding protein, mitochondrial OS=Mus musculus GN	sp|Q9D8T7|SLIRP_MOUSE	13 kDa	11	3

Cytochrome b-c1 complex subunit 2, mitochondrial OS=Mus musculus GN=Uqcrc2 PE	sp|Q9DB77|QCR2_MOUSE	48 kDa	0	13
AH receptor-interacting protein OS=Homo sapiens GN=AIP PE=1 SV=2	sp|O00170|AIP_HUMAN (+1)	38 kDa	3	0
Dynactin subunit 5 OS=Homo sapiens GN=DCTN5 PE=1 SV=1	sp|Q9BTE1|DCTN5_HUMAN (+1)	20 kDa	3	0
Mitochondrial inner membrane protein OS=Mus musculus GN=Immt PE=1 SV=1	sp|Q8CAQ8|IMMT_MOUSE	84 kDa	0	14
UBX domain-containing protein 4 OS=Mus musculus GN=Ubxn4 PE=1 SV=1	sp|Q8VCH8|UBXN4_MOUSE	56 kDa	3	0
Hemoglobin subunit beta-1 OS=Mus musculus GN=Hbb-b1 PE=1 SV=2	sp|P02088|HBB1_MOUSE	16 kDa	12	9
Xin actin-binding repeat-containing protein 1 OS=Mus musculus GN=Xirp1 PE=1 SV=2	sp|O70373|XIRP1_MOUSE	123 kDa	5	8
Actin-related protein 2/3 complex subunit 1A OS=Mus musculus GN=Arpc1a PE=1 SV	sp|Q9R0Q6|ARC1A_MOUSE	42 kDa	5	8
Peroxiredoxin-2 OS=Mus musculus GN=Prdx2 PE=1 SV=3	sp|Q61171|PRDX2_MOUSE	22 kDa	9	7
Serine/threonine-protein phosphatase 6 regulatory subunit 3 OS=Mus musculus GN=	sp|Q922D4|PP6R3_MOUSE	95 kDa	9	4
Glutathione S-transferase Mu 1 OS=Mus musculus GN=Gstm1 PE=1 SV=2	sp|P10649|GSTM1_MOUSE	26 kDa	6	5
Galactokinase OS=Mus musculus GN=Galk1 PE=2 SV=1	sp|Q9R0N0|GALK1_MOUSE	42 kDa	8	4
Fumarate hydratase, mitochondrial OS=Mus musculus GN=Fh PE=1 SV=3	sp|P97807|FUMH_MOUSE	54 kDa	0	11
ADP-ribosylation factor-like protein 8B OS=Mus musculus GN=Arl8b PE=2 SV=1	sp|Q9CQW2|ARL8B_MOUSE (+1)	22 kDa	8	3
Guanine nucleotide-binding protein G(I)/G(S)/G(T) subunit beta-2 OS=Homo sapiens	sp|P62879|GBB2_HUMAN (+1)	37 kDa	11	3
Cysteine and histidine-rich domain-containing protein 1 OS=Homo sapiens GN=CHOR	sp|Q9UHD1|CHRD1_HUMAN	37 kDa	4	7
Vacuolar protein-sorting-associated protein 25 OS=Homo sapiens GN=VPS25 PE=1 SV	sp|Q9BRG1|VPS25_HUMAN (+1)	21 kDa	3	0
Eukaryotic translation initiation factor 3 subunit E OS=Homo sapiens GN=EIF3E PE=1	sp|P60228|EIF3E_HUMAN (+1)	52 kDa	9	4
NADH dehydrogenase [ubiquinone] 1 alpha subcomplex subunit 4 OS=Mus musculus	sp|Q62425|NDUA4_MOUSE	9 kDa	6	7
Farnesyl pyrophosphate synthase OS=Mus musculus GN=Fdps PE=2 SV=1	sp|Q920E5|FPPS_MOUSE	41 kDa	8	5
Voltage-dependent anion-selective channel protein 2 OS=Homo sapiens GN=VDAC2 I	sp|P45880|VDAC2_HUMAN	32 kDa	9	4
Annexin A5 OS=Mus musculus GN=Anxa5 PE=1 SV=1	sp|P48036|ANXA5_MOUSE	36 kDa	2	11
Serine/threonine-protein phosphatase 6 catalytic subunit OS=Homo sapiens GN=PPP	sp|O00743|PPP6_HUMAN (+1)	35 kDa	13	3
Protein FAM161A OS=Homo sapiens GN=FAM161A PE=2 SV=2	sp|Q3B820|F161A_HUMAN	77 kDa	2	2
Ig gamma-3 chain C region OS=Mus musculus PE=1 SV=2	sp|P03987|IGHG3_MOUSE	44 kDa	0	2
Alanyl-tRNA synthetase, cytoplasmic OS=Mus musculus GN=Aars PE=1 SV=1	sp|Q8BGQ7|SYAC_MOUSE	107 kDa	0	12
BAG family molecular chaperone regulator 5 OS=Mus musculus GN=Bag5 PE=1 SV=1	sp|Q8CI32|BAG5_MOUSE	51 kDa	3	0
Adenylyl cyclase-associated protein 1 OS=Mus musculus GN=Cap1 PE=1 SV=4	sp|P40124|CAP1_MOUSE	52 kDa	0	13
ATP synthase subunit b, mitochondrial OS=Mus musculus GN=Atp5f1 PE=1 SV=1	sp|Q9CQQ7|AT5F1_MOUSE	29 kDa	5	6
Casein kinase II subunit beta OS=Homo sapiens GN=CSNK2B PE=1 SV=1	sp|P67870|CSK2B_HUMAN (+1)	25 kDa	3	0
DnaJ homolog subfamily C member 13 OS=Homo sapiens GN=DNAJC13 PE=1 SV=5	sp|O75165|DJC13_HUMAN	254 kDa	7	4
Ubiquitin carboxyl-terminal hydrolase 5 OS=Homo sapiens GN=USP5 PE=1 SV=2	sp|P45974|UBP5_HUMAN (+1)	96 kDa	5	7
Importin-7 OS=Homo sapiens GN=IPO7 PE=1 SV=1	sp|O95373|IPO7_HUMAN (+1)	120 kDa	8	4
Heterogeneous nuclear ribonucleoprotein U-like protein 1 OS=Mus musculus GN=Hn	sp|Q8VDM6|HNRL1_MOUSE (+1)	96 kDa	11	3
Importin-9 OS=Mus musculus GN=Ipo9 PE=1 SV=3	sp|Q91YE6|IPO9_MOUSE (+1)	116 kDa	7	5
Leucine-rich repeat-containing protein 59 OS=Mus musculus GN=Lrrc59 PE=2 SV=1	sp|Q922Q8|LRC59_MOUSE	35 kDa	9	2
Serine/threonine-protein phosphatase PP1-beta catalytic subunit OS=Homo sapiens	sp|P62140|PP1B_HUMAN (+1)	37 kDa	8	3
Heterogeneous nuclear ribonucleoprotein A/B OS=Mus musculus GN=Hnrnpab PE=1	sp|Q99020|ROAA_MOUSE	31 kDa	5	8
AP-4 complex subunit mu-1 OS=Homo sapiens GN=AP4M1 PE=1 SV=2	sp|O00189|AP4M1_HUMAN (+1)	50 kDa	3	0
Chaperone activity of bc1 complex-like, mitochondrial OS=Mus musculus GN=Adck3 I	sp|Q60936|ADCK3_MOUSE	72 kDa	9	2
Coiled-coil domain-containing protein 47 OS=Mus musculus GN=Ccdc47 PE=2 SV=2	sp|Q9D024|CC47_MOUSE	56 kDa	3	8
Alpha-actinin-4 OS=Homo sapiens GN=ACTN4 PE=1 SV=2	sp|O43707|ACTN4_HUMAN	105 kDa	0	40
L-lactate dehydrogenase B chain OS=Homo sapiens GN=LDHB PE=1 SV=2	sp|P07195|LDHB_HUMAN (+1)	37 kDa	8	9
ADP-ribosylation factor 4 OS=Homo sapiens GN=ARF4 PE=1 SV=3	sp|P18085|ARF4_HUMAN	21 kDa	6	13
Ubiquitin-conjugating enzyme E2 N OS=Homo sapiens GN=UBE2N PE=1 SV=1	sp|P61088|UBE2N_HUMAN (+1)	17 kDa	4	8
Synaptojanin-2-binding protein OS=Mus musculus GN=Synj2bp PE=1 SV=1	sp|Q9D6K5|SYJ2B_MOUSE	16 kDa	3	9
Prohibitin-2 OS=Mus musculus GN=Phb2 PE=1 SV=1	sp|O35129|PHB2_MOUSE (+1)	33 kDa	2	10
V-type proton ATPase subunit d 1 OS=Mus musculus GN=Atp6v0d1 PE=1 SV=2	sp|P51863|VA0D1_MOUSE (+1)	40 kDa	2	10
Glycine amidinotransferase, mitochondrial OS=Mus musculus GN=Gatm PE=1 SV=1	sp|Q9D964|GATM_MOUSE	48 kDa	10	2
Hsc70-interacting protein OS=Mus musculus GN=St13 PE=2 SV=1	sp|Q99L47|F10A1_MOUSE	42 kDa	0	11
Asparagine synthetase [glutamine-hydrolyzing] OS=Mus musculus GN=Asns PE=2 SV=	sp|Q61024|ASNS_MOUSE	64 kDa	0	11
Rab5 GDP/GTP exchange factor OS=Mus musculus GN=Rabgef1 PE=1 SV=1	sp|Q9JM13|RABX5_MOUSE	57 kDa	3	0
Leucine-rich repeat flightless-interacting protein 2 OS=Homo sapiens GN=LRRFIP2 PE	sp|Q9Y608|LRRF2_HUMAN	82 kDa	0	3
Protein TANC1 OS=Mus musculus GN=Tanc1 PE=1 SV=2	sp|Q0VGY8|TANC1_MOUSE	201 kDa	3	0
Microtubule-associated serine/threonine-protein kinase 4 OS=Homo sapiens GN=MA	sp|O15021|MAST4_HUMAN (+1)	284 kDa	3	0
Rab GDP dissociation inhibitor beta OS=Mus musculus GN=Gdi2 PE=1 SV=1	sp|Q61598|GDIB_MOUSE	51 kDa	0	12
AFG3-like protein 2 OS=Mus musculus GN=Afg3l2 PE=1 SV=1	sp|Q8JZQ2|AFG32_MOUSE	90 kDa	0	12
Tubulin beta-4 chain OS=Homo sapiens GN=TUBB4 PE=1 SV=2	sp|P04350|TBB4_HUMAN (+1)	50 kDa	49	43
Collagen alpha-1(I) chain OS=Homo sapiens GN=COL1A1 PE=1 SV=5	sp|P02452|CO1A1_HUMAN (+1)	139 kDa	2	5
Protein S100-A8 OS=Homo sapiens GN=S100A8 PE=1 SV=1	sp|P05109|S10A8_HUMAN	11 kDa	4	7
Four and a half LIM domains protein 2 OS=Mus musculus GN=Fhl2 PE=1 SV=1	sp|O70433|FHL2_MOUSE	32 kDa	7	2
Importin subunit alpha-4 OS=Mus musculus GN=Kpna4 PE=2 SV=1	sp|O35343|IMA4_MOUSE	58 kDa	6	2
Protein transport protein Sec61 subunit alpha isoform 1 OS=Homo sapiens GN=SEC6	sp|P61619|S61A1_HUMAN (+3)	52 kDa	3	2
Heterogeneous nuclear ribonucleoprotein G OS=Mus musculus GN=Rbmx PE=1 SV=1	sp|O35479|HNRPG_MOUSE (+1)	42 kDa	6	3
Septin-11 OS=Mus musculus GN=Sept11 PE=1 SV=4	sp|Q8C1B7|SEP11_MOUSE (+1)	50 kDa	3	8
Alpha-centractin OS=Homo sapiens GN=ACTR1A PE=1 SV=1	sp|P61163|ACTZ_HUMAN (+1)	43 kDa	6	11
Skin-specific protein 32 OS=Homo sapiens GN=XP32 PE=1 SV=1	sp|Q5T750|XP32_HUMAN	26 kDa	5	5
Heat shock protein 105 kDa OS=Mus musculus GN=Hsph1 PE=1 SV=2	sp|Q61699|HS105_MOUSE	96 kDa	6	9
Pentatricopeptide repeat-containing protein 3, mitochondrial OS=Mus musculus GN=	sp|Q14C51|PTCD3_MOUSE	78 kDa	8	3
Importin subunit alpha-1 OS=Homo sapiens GN=KPNA1 PE=1 SV=3	sp|P52294|IMA1_HUMAN (+1)	60 kDa	8	3
Beta-2-microglobulin OS=Mus musculus GN=B2m PE=1 SV=1	sp|P01887|B2MG_MOUSE	14 kDa	2	9
Protein FAM115C OS=Mus musculus GN=Fam115c PE=2 SV=1	sp|Q921K8|F115C_MOUSE	102 kDa	8	3
Cullin-4A OS=Mus musculus GN=Cul4a PE=1 SV=1	sp|Q3TCH7|CUL4A_MOUSE	88 kDa	3	8
Myotubularin-related protein 1 OS=Mus musculus GN=Mtmr1 PE=1 SV=1	sp|Q9Z2C4|MTMR1_MOUSE	75 kDa	3	0
SWI/SNF-related matrix-associated actin-dependent regulator of chromatin subfamil	sp|O60264|SMCA5_HUMAN (+1)	122 kDa	3	0
Staphylococcal nuclease domain-containing protein 1 OS=Mus musculus GN=Snd1 PE	sp|Q78PY7|SND1_MOUSE	102 kDa	0	10
Bifunctional purine biosynthesis protein PURH OS=Mus musculus GN=Atic PE=1 SV=2	sp|Q9CWJ9|PUR9_MOUSE	64 kDa	0	10

COP9 signalosome complex subunit 6 OS=Mus musculus GN=Cops6 PE=1 SV=1	sp|O88545|CSN6_MOUSE (+1)	36 kDa	3	0
Calnexin OS=Mus musculus GN=Canx PE=1 SV=1	sp|P35564|CALX_MOUSE	67 kDa	0	11
Drebrin OS=Homo sapiens GN=DBN1 PE=1 SV=4	sp|Q16643|DREB_HUMAN (+1)	71 kDa	0	11
Transaldolase OS=Mus musculus GN=Taldo1 PE=1 SV=2	sp|Q93092|TALDO_MOUSE	37 kDa	0	11
Cytoplasmic protein NCK1 OS=Homo sapiens GN=NCK1 PE=1 SV=1	sp|P16333|NCK1_HUMAN	43 kDa	3	0
Cytochrome c1, heme protein, mitochondrial OS=Mus musculus GN=Cyc1 PE=1 SV=1	sp|Q9D0M3|CY1_MOUSE	35 kDa	0	11
Coatomer subunit gamma OS=Mus musculus GN=Copg PE=2 SV=1	sp|Q9QZE5|COPG_MOUSE	98 kDa	0	11
Protein transport protein Sec31A OS=Mus musculus GN=Sec31a PE=1 SV=2	sp|Q3UPL0|SC31A_MOUSE	134 kDa	6	2
RNA-binding protein EWS OS=Homo sapiens GN=EWSR1 PE=1 SV=1	sp|Q01844|EWS_HUMAN (+1)	68 kDa	5	2
Carbonic anhydrase 3 OS=Mus musculus GN=Ca3 PE=1 SV=3	sp|P16015|CAH3_MOUSE	29 kDa	3	5
Peroxiredoxin-1 OS=Homo sapiens GN=PRDX1 PE=1 SV=1	sp|Q06830|PRDX1_HUMAN	22 kDa	13	10
Protein FAM54B OS=Mus musculus GN=Fam54b PE=1 SV=1	sp|Q9CWE0|FA54B_MOUSE	32 kDa	5	4
Protein S100-A4 OS=Mus musculus GN=S100a4 PE=1 SV=1	sp|P07091|S10A4_MOUSE	12 kDa	6	4
40S ribosomal protein S26 OS=Homo sapiens GN=RPS26 PE=1 SV=3	sp|P62854|RS26_HUMAN (+2)	13 kDa	6	2
Translation initiation factor eIF-2B subunit epsilon OS=Mus musculus GN=Eif2b5 PE=1	sp|Q8CHW4|EI2BE_MOUSE	80 kDa	3	5
Ras-related C3 botulinum toxin substrate 1 OS=Homo sapiens GN=RAC1 PE=1 SV=1	sp|P63000|RAC1_HUMAN (+1)	21 kDa	3	7
Leucine-rich repeat flightless-interacting protein 1 OS=Mus musculus GN=Lrrfip1 PE=1	sp|Q3UZ39|LRRF1_MOUSE	79 kDa	2	8
Peroxiredoxin-6 OS=Homo sapiens GN=PRDX6 PE=1 SV=3	sp|P30041|PRDX6_HUMAN	25 kDa	7	3
Apoptosis-inducing factor 2 OS=Mus musculus GN=Aifm2 PE=2 SV=1	sp|Q8BUE4|AIFM2_MOUSE	41 kDa	3	0
Calpain-1 catalytic subunit OS=Mus musculus GN=Capn1 PE=2 SV=1	sp|O35350|CAN1_MOUSE	82 kDa	3	8
Superoxide dismutase [Cu-Zn] OS=Mus musculus GN=Sod1 PE=1 SV=2	sp|P08228|SODC_MOUSE	16 kDa	0	8
26S proteasome non-ATPase regulatory subunit 8 OS=Mus musculus GN=Psmd8 PE=2	sp|Q9CX56|PSMD8_MOUSE	40 kDa	2	7
LIM domain and actin-binding protein 1 OS=Mus musculus GN=Lima1 PE=1 SV=3	sp|Q9ERG0|LIMA1_MOUSE	84 kDa	0	9
Succinate dehydrogenase [ubiquinone] iron-sulfur subunit, mitochondrial OS=Mus m	sp|Q9CQA3|DHSB_MOUSE	32 kDa	5	4
Vesicle-trafficking protein SEC22b OS=Mus musculus GN=Sec22b PE=1 SV=3	sp|O08547|SC22B_MOUSE	25 kDa	5	5
26S proteasome non-ATPase regulatory subunit 3 OS=Mus musculus GN=Psmd3 PE=2	sp|P14685|PSMD3_MOUSE	61 kDa	4	6
Glutaminyl-tRNA synthetase OS=Homo sapiens GN=QARS PE=1 SV=1	sp|P47897|SYQ_HUMAN	88 kDa	5	5
Fructose-bisphosphate aldolase B OS=Mus musculus GN=Aldob PE=1 SV=3	sp|Q91Y97|ALDOB_MOUSE	40 kDa	6	6
Actin-related protein 2/3 complex subunit 5 OS=Homo sapiens GN=ARPC5 PE=1 SV=3	sp|O15511|ARPC5_HUMAN (+1)	16 kDa	4	6
MOSC domain-containing protein 2, mitochondrial OS=Mus musculus GN=Mosc2 PE=	sp|Q922Q1|MOSC2_MOUSE	38 kDa	3	7
V-type proton ATPase catalytic subunit A OS=Mus musculus GN=Atp6v1a PE=1 SV=2	sp|P50516|VATA_MOUSE	68 kDa	5	5
Mitotic checkpoint protein BUB3 OS=Homo sapiens GN=BUB3 PE=1 SV=1	sp|O43684|BUB3_HUMAN (+1)	37 kDa	8	3
NADH dehydrogenase [ubiquinone] 1 alpha subcomplex subunit 5 OS=Mus musculus	sp|Q9CPP6|NDUA5_MOUSE	13 kDa	3	7
Cullin-associated NEDD8-dissociated protein 2 OS=Mus musculus GN=Cand2 PE=1 SV	sp|Q6ZQ73|CAND2_MOUSE	136 kDa	9	4
OTU domain-containing protein 4 OS=Mus musculus GN=Otud4 PE=1 SV=1	sp|B2RRE7|OTUD4_MOUSE (+1)	123 kDa	3	0
Methylcrotonoyl-CoA carboxylase subunit alpha, mitochondrial OS=Mus musculus GN	sp|Q99MR8|MCCA_MOUSE	79 kDa	0	9
Prolyl endopeptidase OS=Mus musculus GN=Prep PE=2 SV=1	sp|Q9QUR6|PPCE_MOUSE	81 kDa	0	9
Thioredoxin reductase 1, cytoplasmic OS=Mus musculus GN=Txnrd1 PE=1 SV=3	sp|Q9JMH6|TRXR1_MOUSE	67 kDa	0	2
Advillin OS=Mus musculus GN=Avil PE=1 SV=2	sp|O88398|AVIL_MOUSE	92 kDa	0	9
Aspartate aminotransferase, cytoplasmic OS=Mus musculus GN=Got1 PE=1 SV=3	sp|P05201|AATC_MOUSE	46 kDa	0	10
DNA replication licensing factor MCM3 OS=Homo sapiens GN=MCM3 PE=1 SV=3	sp|P25205|MCM3_HUMAN (+1)	91 kDa	3	0
6-phosphogluconate dehydrogenase, decarboxylating OS=Mus musculus GN=Pgd PE=	sp|Q9DCD0|6PGD_MOUSE	53 kDa	0	10
Probable ATP-dependent RNA helicase DHX36 OS=Mus musculus GN=Dhx36 PE=2 SV	sp|Q8VHK9|DHX36_MOUSE	114 kDa	3	0
N-alpha-acetyltransferase 10, NatA catalytic subunit OS=Homo sapiens GN=NAA10 PE	sp|P41227|NAA10_HUMAN (+2)	26 kDa	3	0
NAD(P) transhydrogenase, mitochondrial OS=Homo sapiens GN=NNT PE=1 SV=3	sp|Q13423|NNTM_HUMAN (+1)	114 kDa	2	5
Protein piccolo OS=Homo sapiens GN=PCLO PE=1 SV=3	sp|Q9Y6V0|PCLO_HUMAN	567 kDa	0	2
Histone H2B type 1-K OS=Homo sapiens GN=HIST1H2BK PE=1 SV=3	sp|O60814|H2B1K_HUMAN (+16)	14 kDa	7	2
NADH dehydrogenase [ubiquinone] iron-sulfur protein 8, mitochondrial OS=Mus mus	sp|Q8K3J1|NDUS8_MOUSE	24 kDa	6	2
Chloride intracellular channel protein 4 OS=Mus musculus GN=Clic4 PE=1 SV=3	sp|Q9QYB1|CLIC4_MOUSE (+1)	29 kDa	2	6
RNA-binding Raly-like protein OS=Homo sapiens GN=RALYL PE=1 SV=2	sp|Q86SE5|RALYL_HUMAN (+1)	32 kDa	0	2
Endophilin-B1 OS=Mus musculus GN=Sh3glb1 PE=1 SV=1	sp|Q9JK48|SHLB1_MOUSE	41 kDa	2	5
Isocitrate dehydrogenase [NAD] subunit gamma 1, mitochondrial OS=Mus musculus (	sp|P70404|IDHG1_MOUSE	43 kDa	3	6
Collagen alpha-2(V) chain OS=Mus musculus GN=Col5a2 PE=1 SV=1	sp|Q3U962|CO5A2_MOUSE	145 kDa	5	3
Emerin OS=Mus musculus GN=Emd PE=1 SV=1	sp|O08579|EMD_MOUSE	29 kDa	5	4
Translocon-associated protein subunit delta OS=Mus musculus GN=Ssr4 PE=2 SV=1	sp|Q62186|SSRD_MOUSE	19 kDa	3	6
39S ribosomal protein L14, mitochondrial OS=Mus musculus GN=Mrpl14 PE=2 SV=1	sp|Q9D1I6|RM14_MOUSE	16 kDa	5	4
BolA-like protein 2 OS=Homo sapiens GN=BOLA2 PE=1 SV=1	sp|Q9H3K6|BOLA2_HUMAN	10 kDa	4	5
Cullin-3 OS=Mus musculus GN=Cul3 PE=1 SV=1	sp|Q9JLV5|CUL3_MOUSE	89 kDa	5	5
26S proteasome non-ATPase regulatory subunit 11 OS=Homo sapiens GN=PSMD11 P	sp|O00231|PSD11_HUMAN (+1)	47 kDa	3	6
Betaine--homocysteine S-methyltransferase 1 OS=Mus musculus GN=Bhmt PE=2 SV=	sp|O35490|BHMT1_MOUSE	45 kDa	3	6
Protein SEC13 homolog OS=Mus musculus GN=Sec13 PE=2 SV=3	sp|Q9D1M0|SEC13_MOUSE	36 kDa	5	4
Ubiquitin-conjugating enzyme E2 D3 OS=Homo sapiens GN=UBE2D3 PE=1 SV=1	sp|P61077|UB2D3_HUMAN (+1)	17 kDa	4	5
10 kDa heat shock protein, mitochondrial OS=Mus musculus GN=Hspe1 PE=1 SV=2	sp|Q64433|CH10_MOUSE	11 kDa	0	8
ATP synthase subunit f, mitochondrial OS=Mus musculus GN=Atp5j2 PE=1 SV=3	sp|P56135|ATPK_MOUSE	10 kDa	0	8
Septin-2 OS=Mus musculus GN=Sept2 PE=1 SV=2	sp|P42208|SEPT2_MOUSE	42 kDa	0	8
Septin-9 OS=Mus musculus GN=Sept9 PE=1 SV=1	sp|Q80UG5|SEPT9_MOUSE	66 kDa	0	8
NADH dehydrogenase [ubiquinone] 1 alpha subcomplex subunit 13 OS=Mus musculu	sp|Q9ERS2|NDUAD_MOUSE	17 kDa	0	7
Protein-L-isoaspartate O-methyltransferase domain-containing protein 1 OS=Mus mu	sp|P59913|PCMD1_MOUSE (+1)	41 kDa	0	9
Cytochrome c, somatic OS=Mus musculus GN=Cycs PE=1 SV=2	sp|P62897|CYC_MOUSE	12 kDa	0	9
28S ribosomal protein S17, mitochondrial OS=Mus musculus GN=Mrps17 PE=2 SV=1	sp|Q9CQE3|RT17_MOUSE	13 kDa	3	0
Acetyl-CoA acetyltransferase, mitochondrial OS=Mus musculus GN=Acat1 PE=1 SV=1	sp|Q8QZT1|THIL_MOUSE	45 kDa	0	9
NADH-ubiquinone oxidoreductase 75 kDa subunit, mitochondrial OS=Mus musculus (	sp|Q91VD9|NDUS1_MOUSE	80 kDa	0	9
Small nuclear ribonucleoprotein E OS=Homo sapiens GN=SNRPE PE=1 SV=1	sp|P62304|RUXE_HUMAN (+2)	11 kDa	3	0
Adenylyl cyclase-associated protein 2 OS=Mus musculus GN=Cap2 PE=1 SV=1	sp|Q9CYT6|CAP2_MOUSE	53 kDa	0	11
Solute carrier family 2, facilitated glucose transporter member 3 OS=Homo sapiens G	sp|P11169|GTR3_HUMAN (+1)	54 kDa	4	2
Retinoid-inducible serine carboxypeptidase OS=Mus musculus GN=Scpep1 PE=2 SV=2	sp|Q920A5|RISC_MOUSE	51 kDa	4	2
THO complex subunit 4 OS=Mus musculus GN=Thoc4 PE=1 SV=3	sp|O08583|THOC4_MOUSE (+1)	27 kDa	3	0

ADP-ribosylation factor 5 OS=Mus musculus GN=Arf5 PE=2 SV=2	sp|P84084|ARF5_MOUSE (+1)	21 kDa	6	17
EF-hand domain-containing family member A1 OS=Mus musculus GN=Efha1 PE=2 SV	sp|Q8CD10|EFHA1_MOUSE	49 kDa	6	2
Platelet-activating factor acetylhydrolase IB subunit alpha OS=Homo sapiens GN=PAF	sp|P43034|LIS1_HUMAN (+1)	47 kDa	2	5
Troponin T, slow skeletal muscle OS=Mus musculus GN=Tnnt1 PE=2 SV=3	sp|O88346|TNNT1_MOUSE	31 kDa	5	7
DnaJ homolog subfamily C member 10 OS=Mus musculus GN=Dnajc10 PE=1 SV=2	sp|Q9DC23|DJC10_MOUSE	91 kDa	4	3
Glutathione S-transferase A3 OS=Mus musculus GN=Gsta3 PE=1 SV=2	sp|P30115|GSTA3_MOUSE	25 kDa	3	4
Mannose-1-phosphate guanyltransferase alpha OS=Mus musculus GN=Gmppa PE=2 S	sp|Q922H4|GMPPA_MOUSE	46 kDa	3	0
Annexin A11 OS=Mus musculus GN=Anxa11 PE=1 SV=2	sp|P97384|ANX11_MOUSE	54 kDa	0	7
Alpha-mannosidase 2C1 OS=Mus musculus GN=Man2c1 PE=1 SV=1	sp|Q91W89|MA2C1_MOUSE	116 kDa	5	2
Serine/threonine-protein phosphatase 2B catalytic subunit beta isoform OS=Homo sa	sp|P16298|PP2BB_HUMAN (+1)	59 kDa	4	4
NADH dehydrogenase [ubiquinone] 1 alpha subcomplex subunit 12 OS=Mus musculu	sp|Q7TMF3|NDUAC_MOUSE	17 kDa	3	5
Phenylalanyl-tRNA synthetase beta chain OS=Mus musculus GN=Farsb PE=2 SV=2	sp|Q9WUA2|SYFB_MOUSE	66 kDa	4	4
60S ribosomal protein L22-like 1 OS=Homo sapiens GN=RPL22L1 PE=1 SV=2	sp|Q6P5R6|RL22L_HUMAN (+1)	15 kDa	9	6
Short-chain specific acyl-CoA dehydrogenase, mitochondrial OS=Mus musculus GN=A	sp|Q07417|ACADS_MOUSE	45 kDa	4	4
GTP-binding protein SAR1a OS=Homo sapiens GN=SAR1A PE=1 SV=1	sp|Q9NR31|SAR1A_HUMAN	22 kDa	5	3
Calmodulin OS=Homo sapiens GN=CALM1 PE=1 SV=2	sp|P62158|CALM_HUMAN (+1)	17 kDa	3	7
Valyl-tRNA synthetase OS=Mus musculus GN=Vars PE=2 SV=1	sp|Q9Z1Q9|SYVC_MOUSE	140 kDa	4	4
UDP-glucose 6-dehydrogenase OS=Mus musculus GN=Ugdh PE=1 SV=1	sp|O70475|UGDH_MOUSE	55 kDa	5	3
Procollagen galactosyltransferase 1 OS=Mus musculus GN=Glt25d1 PE=1 SV=2	sp|Q8K297|GT251_MOUSE	71 kDa	4	4
Cullin-1 OS=Homo sapiens GN=CUL1 PE=1 SV=2	sp|Q13616|CUL1_HUMAN (+1)	90 kDa	6	3
Fatty acid-binding protein, liver OS=Mus musculus GN=Fabp1 PE=1 SV=2	sp|P12710|FABPL_MOUSE	14 kDa	3	5
Procollagen-lysine,2-oxoglutarate 5-dioxygenase 2 OS=Homo sapiens GN=PLOD2 PE=	sp|O00469|PLOD2_HUMAN	85 kDa	22	18
Malectin OS=Mus musculus GN=Mlec PE=2 SV=2	sp|Q6ZQI3|MLEC_MOUSE	32 kDa	2	6
40S ribosomal protein S21 OS=Mus musculus GN=Rps21 PE=2 SV=1	sp|Q9CQR2|RS21_MOUSE	9 kDa	6	2
Caveolin-3 OS=Mus musculus GN=Cav3 PE=1 SV=1	sp|P51637|CAV3_MOUSE	17 kDa	3	5
Annexin A3 OS=Mus musculus GN=Anxa3 PE=1 SV=4	sp|O35639|ANXA3_MOUSE	36 kDa	0	10
5'-nucleotidase domain-containing protein 2 OS=Homo sapiens GN=NT5DC2 PE=1 SV	sp|Q9H857|NT5D2_HUMAN	61 kDa	3	0
X-ray repair cross-complementing protein 6 OS=Mus musculus GN=Xrcc6 PE=1 SV=5	sp|P23475|XRCC6_MOUSE	69 kDa	3	0
26S proteasome non-ATPase regulatory subunit 12 OS=Mus musculus GN=Psmd12 PE	sp|Q9D8W5|PSD12_MOUSE	53 kDa	2	5
Alcohol dehydrogenase 1 OS=Mus musculus GN=Adh1 PE=2 SV=2	sp|P00329|ADH1_MOUSE	40 kDa	3	0
Ubiquitin-conjugating enzyme E2 D1 OS=Homo sapiens GN=UBE2D1 PE=1 SV=1	sp|P51668|UB2D1_HUMAN (+1)	17 kDa	3	0
26S proteasome non-ATPase regulatory subunit 13 OS=Mus musculus GN=Psmd13 PE	sp|Q9WVJ2|PSD13_MOUSE	43 kDa	0	7
Metalloproteinase inhibitor 2 OS=Mus musculus GN=Timp2 PE=1 SV=2	sp|P25785|TIMP2_MOUSE	24 kDa	0	7
Cytospin-A OS=Mus musculus GN=Specc1l PE=1 SV=1	sp|Q2KN98|CYTSA_MOUSE	124 kDa	0	7
Barrier-to-autointegration factor OS=Mus musculus GN=Banf1 PE=1 SV=1	sp|O54962|BAF_MOUSE	10 kDa	3	0
WD and tetratricopeptide repeats protein 1 OS=Mus musculus GN=Wdtc1 PE=1 SV=1	sp|Q80ZK9|WDTC1_MOUSE (+1)	76 kDa	0	7
Serine/arginine-rich splicing factor 5 OS=Mus musculus GN=Srsf5 PE=1 SV=1	sp|O35326|SRSF5_MOUSE (+1)	31 kDa	3	0
Calumenin OS=Mus musculus GN=Calu PE=1 SV=1	sp|O35887|CALU_MOUSE	37 kDa	0	8
Very long-chain specific acyl-CoA dehydrogenase, mitochondrial OS=Mus musculus G	sp|P50544|ACADV_MOUSE	71 kDa	0	8
Synaptic vesicle membrane protein VAT-1 homolog OS=Mus musculus GN=Vat1 PE=1	sp|Q62465|VAT1_MOUSE	43 kDa	0	8
39S ribosomal protein L3, mitochondrial OS=Mus musculus GN=Mrpl3 PE=2 SV=1	sp|Q99N95|RM03_MOUSE	39 kDa	3	0
60S acidic ribosomal protein P1 OS=Mus musculus GN=Rplp1 PE=1 SV=1	sp|P47955|RLA1_MOUSE	11 kDa	3	0
Proteasomal ubiquitin receptor ADRM1 OS=Mus musculus GN=Adrm1 PE=1 SV=2	sp|Q9JKV1|ADRM1_MOUSE	42 kDa	0	8
Peptidyl-tRNA hydrolase 2, mitochondrial OS=Mus musculus GN=Ptrh2 PE=2 SV=1	sp|Q8R2Y8|PTH2_MOUSE	20 kDa	3	0
U6 snRNA-associated Sm-like protein LSm4 OS=Homo sapiens GN=LSM4 PE=1 SV=1	sp|Q9Y4Z0|LSM4_HUMAN	15 kDa	3	0
Putative adenosylhomocysteinase 2 OS=Homo sapiens GN=AHCYL1 PE=1 SV=2	sp|O43865|SAHH2_HUMAN (+1)	59 kDa	3	0
AP-1 complex subunit beta-1 OS=Homo sapiens GN=AP1B1 PE=1 SV=2	sp|Q10567|AP1B1_HUMAN	105 kDa	0	3
Serine/threonine-protein kinase SRPK2 OS=Mus musculus GN=Srpk2 PE=1 SV=2	sp|O54781|SRPK2_MOUSE (+1)	77 kDa	3	0
Trimeric intracellular cation channel type A OS=Mus musculus GN=Tmem38a PE=1 S\	sp|Q3TMP8|TM38A_MOUSE	33 kDa	3	2
Cornifin-B OS=Homo sapiens GN=SPRR1B PE=1 SV=2	sp|P22528|SPR1B_HUMAN (+1)	10 kDa	0	4
Collagen alpha-2(I) chain OS=Homo sapiens GN=COL1A2 PE=1 SV=7	sp|P08123|CO1A2_HUMAN	129 kDa	0	2
Hemopexin OS=Homo sapiens GN=HPX PE=1 SV=2	sp|P02790|HEMO_HUMAN	52 kDa	2	3
28S ribosomal protein S23, mitochondrial OS=Mus musculus GN=Mrps23 PE=2 SV=1	sp|Q8VE22|RT23_MOUSE	20 kDa	3	0
Lactoylglutathione lyase OS=Mus musculus GN=Glo1 PE=1 SV=3	sp|Q9CPU0|LGUL_MOUSE	21 kDa	0	5
RuvB-like 2 OS=Mus musculus GN=Ruvbl2 PE=2 SV=3	sp|Q9WTM5|RUVB2_MOUSE	51 kDa	4	2
Glycogen debranching enzyme OS=Homo sapiens GN=AGL PE=1 SV=3	sp|P35573|GDE_HUMAN	175 kDa	2	4
Myb-binding protein 1A OS=Mus musculus GN=Mybbp1a PE=1 SV=2	sp|Q7TPV4|MBB1A_MOUSE	152 kDa	3	0
N-alpha-acetyltransferase 15, NatA auxiliary subunit OS=Mus musculus GN=Naa15 PE	sp|Q80UM3|NAA15_MOUSE	101 kDa	4	2
Low molecular weight phosphotyrosine protein phosphatase OS=Homo sapiens GN=/	sp|P24666|PPAC_HUMAN (+1)	18 kDa	3	2
Malonyl-CoA-acyl carrier protein transacylase, mitochondrial OS=Mus musculus GN=I	sp|Q8R3F5|FABD_MOUSE	42 kDa	3	0
Thioredoxin-dependent peroxide reductase, mitochondrial OS=Mus musculus GN=Pr	sp|P20108|PRDX3_MOUSE	28 kDa	4	3
DNA-directed RNA polymerases I, II, and III subunit RPABC3 OS=Homo sapiens GN=P(	sp|P52434|RPAB3_HUMAN (+1)	17 kDa	3	4
IgG receptor FcRn large subunit p51 OS=Mus musculus GN=Fcgrt PE=1 SV=1	sp|Q61559|FCGRN_MOUSE	40 kDa	0	7
40S ribosomal protein S28 OS=Homo sapiens GN=RPS28 PE=1 SV=1	sp|P62857|RS28_HUMAN (+1)	8 kDa	3	4
CDP-diacylglycerol--inositol 3-phosphatidyltransferase OS=Mus musculus GN=Cdipt P	sp|Q8VDP6|CDIPT_MOUSE	24 kDa	3	4
Dynein light chain Tctex-type 1 OS=Mus musculus GN=Dynlt1 PE=1 SV=1	sp|P51807|DYLT1_MOUSE	12 kDa	3	4
Peptidyl-prolyl cis-trans isomerase B OS=Mus musculus GN=Ppib PE=2 SV=2	sp|P24369|PPIB_MOUSE	24 kDa	3	6
Allograft inflammatory factor 1-like OS=Mus musculus GN=Aif1l PE=2 SV=1	sp|Q9EQX4|AIF1L_MOUSE	17 kDa	3	4
Developmentally-regulated GTP-binding protein 1 OS=Mus musculus GN=Drg1 PE=1 S	sp|P32233|DRG1_MOUSE (+1)	41 kDa	4	3
Prolactin regulatory element-binding protein OS=Mus musculus GN=Preb PE=1 SV=1	sp|Q9WUQ2|PREB_MOUSE	45 kDa	2	5
Alpha-globin transcription factor CP2 OS=Mus musculus GN=Tcfcp2 PE=1 SV=1	sp|Q9ERA0|TFCP2_MOUSE	57 kDa	4	4
Eukaryotic translation initiation factor 3 subunit M OS=Homo sapiens GN=EIF3M PE=	sp|Q7L2H7|EIF3M_HUMAN (+1)	43 kDa	3	3
Eukaryotic translation initiation factor 1A OS=Mus musculus GN=Eif1a PE=2 SV=3	sp|Q60872|IF1A_MOUSE	17 kDa	0	6
Eukaryotic translation initiation factor 3 subunit F OS=Homo sapiens GN=EIF3F PE=1 S	sp|O00303|EIF3F_HUMAN (+1)	38 kDa	5	2
Prolyl 3-hydroxylase 1 OS=Mus musculus GN=Lepre1 PE=2 SV=2	sp|Q3V1T4|P3H1_MOUSE	84 kDa	0	5
Tryptophanyl-tRNA synthetase, cytoplasmic OS=Mus musculus GN=Wars PE=1 SV=2	sp|P32921|SYWC_MOUSE	54 kDa	0	6
Cofilin-2 OS=Mus musculus GN=Cfl2 PE=1 SV=1	sp|P45591|COF2_MOUSE (+1)	19 kDa	0	8

Peroxiredoxin-5, mitochondrial OS=Mus musculus GN=Prdx5 PE=1 SV=2	sp|P99029|PRDX5_MOUSE	22 kDa	0	6
Reticulon-2 OS=Mus musculus GN=Rtn2 PE=1 SV=1	sp|O70622|RTN2_MOUSE	51 kDa	0	6
Ubiquitin-like protein 5 OS=Homo sapiens GN=UBL5 PE=1 SV=1	sp|Q9BZL1|UBL5_HUMAN (+1)	9 kDa	3	0
Integrin beta-1 OS=Mus musculus GN=Itgb1 PE=1 SV=1	sp|P09055|ITB1_MOUSE	88 kDa	0	6
Glycerol-3-phosphate dehydrogenase, mitochondrial OS=Mus musculus GN=Gpd2 PE	sp|Q64521|GPDM_MOUSE	81 kDa	0	6
39S ribosomal protein L11, mitochondrial OS=Mus musculus GN=Mrpl11 PE=2 SV=1	sp|Q9CQF0|RM11_MOUSE	21 kDa	3	0
Multifunctional protein ADE2 OS=Mus musculus GN=Paics PE=1 SV=4	sp|Q9DCL9|PUR6_MOUSE	47 kDa	0	6
Proteasome subunit beta type-7 OS=Mus musculus GN=Psmb7 PE=1 SV=1	sp|P70195|PSB7_MOUSE	30 kDa	0	2
Acyl-CoA dehydrogenase family member 10 OS=Mus musculus GN=Acad10 PE=2 SV=	sp|Q8K370|ACD10_MOUSE	119 kDa	3	0
UTP--glucose-1-phosphate uridylyltransferase OS=Homo sapiens GN=UGP2 PE=1 SV=	sp|Q16851|UGPA_HUMAN (+1)	57 kDa	0	7
ATP-dependent RNA helicase DDX50 OS=Mus musculus GN=Ddx50 PE=2 SV=1	sp|Q99MJ9|DDX50_MOUSE (+1)	82 kDa	3	0
Probable arginyl-tRNA synthetase, mitochondrial OS=Mus musculus GN=Rars2 PE=2 S	sp|Q3U186|SYRM_MOUSE	65 kDa	3	0
Serotransferrin OS=Mus musculus GN=Tf PE=1 SV=1	sp|Q921I1|TRFE_MOUSE	77 kDa	0	8
Phosphoglucomutase-1 OS=Mus musculus GN=Pgm1 PE=1 SV=4	sp|Q9D0F9|PGM1_MOUSE	61 kDa	0	8
Aminoacyl tRNA synthase complex-interacting multifunctional protein 2 OS=Mus mu	sp|Q8R010|AIMP2_MOUSE	35 kDa	3	0
39S ribosomal protein L43, mitochondrial OS=Mus musculus GN=Mrpl43 PE=2 SV=1	sp|Q99N89|RM43_MOUSE	20 kDa	3	0
NADH dehydrogenase [ubiquinone] iron-sulfur protein 5 OS=Mus musculus GN=Nduf	sp|Q99LY9|NDUS5_MOUSE	13 kDa	0	7
Ubiquitin-conjugating enzyme E2 variant 1 OS=Mus musculus GN=Ube2v1 PE=1 SV=1	sp|Q9CZY3|UB2V1_MOUSE	16 kDa	0	7
rRNA 2'-O-methyltransferase fibrillarin OS=Homo sapiens GN=FBL PE=1 SV=2	sp|P22087|FBRL_HUMAN (+1)	34 kDa	3	0
Serpin H1 OS=Homo sapiens GN=SERPINH1 PE=1 SV=2	sp|P50454|SERPH_HUMAN	46 kDa	20	21
Integrin alpha-7 OS=Mus musculus GN=Itga7 PE=1 SV=3	sp|Q61738|ITA7_MOUSE	129 kDa	0	4
Transgelin-2 OS=Mus musculus GN=Tagln2 PE=1 SV=4	sp|Q9WVA4|TAGL2_MOUSE	22 kDa	0	5
Ferrochelatase, mitochondrial OS=Mus musculus GN=Fech PE=1 SV=2	sp|P22315|HEMH_MOUSE	47 kDa	2	3
Elongation factor 1-delta OS=Mus musculus GN=Eef1d PE=1 SV=3	sp|P57776|EF1D_MOUSE	31 kDa	0	4
Filaggrin-2 OS=Homo sapiens GN=FLG2 PE=1 SV=1	sp|Q5D862|FILA2_HUMAN	248 kDa	0	4
Sphingosine-1-phosphate lyase 1 OS=Mus musculus GN=Sgpl1 PE=2 SV=1	sp|Q8R0X7|SGPL1_MOUSE	64 kDa	3	2
Baculoviral IAP repeat-containing protein 6 OS=Homo sapiens GN=BIRC6 PE=1 SV=2	sp|Q9NR09|BIRC6_HUMAN	530 kDa	4	2
Transmembrane 9 superfamily member 2 OS=Mus musculus GN=Tm9sf2 PE=2 SV=1	sp|P58021|TM9S2_MOUSE	75 kDa	0	2
39S ribosomal protein L39, mitochondrial OS=Mus musculus GN=Mrpl39 PE=2 SV=4	sp|Q9JKF7|RM39_MOUSE	39 kDa	3	0
Phosphoribosyl pyrophosphate synthase-associated protein 1 OS=Homo sapiens GN=	sp|Q14558|KPRA_HUMAN (+1)	39 kDa	3	0
Far upstream element-binding protein 2 OS=Mus musculus GN=Khsrp PE=1 SV=2	sp|Q3U0V1|FUBP2_MOUSE	77 kDa	2	3
SET and MYND domain-containing protein 1 OS=Mus musculus GN=Smyd1 PE=1 SV=3	sp|P97443|SMYD1_MOUSE	56 kDa	3	0
Stromal cell-derived factor 2-like protein 1 OS=Mus musculus GN=Sdf2l1 PE=2 SV=2	sp|Q9ESP1|SDF2L_MOUSE	24 kDa	0	2
Cytoplasmic dynein 1 light intermediate chain 1 OS=Mus musculus GN=Dync1li1 PE=1	sp|Q8R1Q8|DC1L1_MOUSE	57 kDa	2	3
Four and a half LIM domains protein 1 OS=Mus musculus GN=Fhl1 PE=2 SV=3	sp|P97447|FHL1_MOUSE	32 kDa	3	3
ADP-ribosylation factor 6 OS=Homo sapiens GN=ARF6 PE=1 SV=2	sp|P62330|ARF6_HUMAN (+1)	20 kDa	5	3
Proteasome assembly chaperone 3 OS=Mus musculus GN=Psmg3 PE=1 SV=1	sp|Q9CZH3|PSMG3_MOUSE	13 kDa	3	3
Small nuclear ribonucleoprotein Sm D2 OS=Homo sapiens GN=SNRPD2 PE=1 SV=1	sp|P62316|SMD2_HUMAN (+1)	14 kDa	2	4
Acyl-CoA dehydrogenase family member 9, mitochondrial OS=Mus musculus GN=Aca	sp|Q8JZN5|ACAD9_MOUSE	69 kDa	2	4
Probable saccharopine dehydrogenase OS=Mus musculus GN=Sccpdh PE=2 SV=1	sp|Q8R127|SCPDH_MOUSE	47 kDa	2	4
Coactosin-like protein OS=Homo sapiens GN=COTL1 PE=1 SV=3	sp|Q14019|COTL1_HUMAN (+1)	16 kDa	2	4
Arginase-1 OS=Mus musculus GN=Arg1 PE=1 SV=1	sp|Q61176|ARGI1_MOUSE	35 kDa	4	2
Small nuclear ribonucleoprotein Sm D3 OS=Homo sapiens GN=SNRPD3 PE=1 SV=1	sp|P62318|SMD3_HUMAN (+1)	14 kDa	3	3
28S ribosomal protein S25, mitochondrial OS=Mus musculus GN=Mrps25 PE=1 SV=1	sp|Q9D125|RT25_MOUSE	20 kDa	3	0
DnaJ homolog subfamily B member 12 OS=Mus musculus GN=Dnajb12 PE=2 SV=2	sp|Q9QYI4|DJB12_MOUSE	42 kDa	4	2
Zinc finger protein 622 OS=Mus musculus GN=Znf622 PE=2 SV=1	sp|Q91VY9|ZN622_MOUSE	53 kDa	3	0
Nucleoside diphosphate kinase B OS=Mus musculus GN=Nme2 PE=1 SV=1	sp|Q01768|NDKB_MOUSE	17 kDa	9	17
Ankycorbin OS=Mus musculus GN=Rai14 PE=1 SV=1	sp|Q9EP71|RAI14_MOUSE	109 kDa	3	3
Heterogeneous nuclear ribonucleoprotein D-like OS=Homo sapiens GN=HNRPDL PE=	sp|O14979|HNRDL_HUMAN (+1)	46 kDa	6	4
Trafficking protein particle complex subunit 3 OS=Mus musculus GN=Trappc3 PE=1 S\	sp|O55013|TPPC3_MOUSE	20 kDa	3	3
28S ribosomal protein S12, mitochondrial OS=Homo sapiens GN=MRPS12 PE=1 SV=1	sp|O15235|RT12_HUMAN (+1)	15 kDa	4	2
Translocon-associated protein subunit alpha OS=Homo sapiens GN=SSR1 PE=1 SV=3	sp|P43307|SSRA_HUMAN (+1)	32 kDa	3	3
Programmed cell death protein 2 OS=Mus musculus GN=Pdcd2 PE=2 SV=2	sp|P46718|PDCD2_MOUSE	38 kDa	4	2
Cleavage and polyadenylation specificity factor subunit 6 OS=Homo sapiens GN=CPSF	sp|Q16630|CPSF6_HUMAN (+1)	59 kDa	3	0
Polyadenylate-binding protein-interacting protein 1 OS=Mus musculus GN=Paip1 PE=	sp|Q8VE62|PAIP1_MOUSE (+1)	46 kDa	3	0
Nardilysin OS=Mus musculus GN=Nrd1 PE=1 SV=1	sp|Q8BHG1|NRDC_MOUSE	133 kDa	4	2
Synaptogyrin-1 OS=Homo sapiens GN=SYNGR1 PE=1 SV=2	sp|O43759|SNG1_HUMAN (+1)	25 kDa	0	5
40S ribosomal protein S27-like OS=Mus musculus GN=Rps27l PE=2 SV=3	sp|Q6ZWY3|RS27L_MOUSE (+1)	9 kDa	0	6
3-hydroxyacyl-CoA dehydratase 3 OS=Homo sapiens GN=PTPLAD1 PE=1 SV=2	sp|Q9P035|HACD3_HUMAN	43 kDa	3	0
Mitotic spindle assembly checkpoint protein MAD2A OS=Homo sapiens GN=MAD2L1	sp|Q13257|MD2L1_HUMAN	24 kDa	3	0
Pre-mRNA-splicing factor 38B OS=Mus musculus GN=Prpf38b PE=1 SV=1	sp|Q80SY5|PR38B_MOUSE	64 kDa	3	0
Exportin-2 OS=Mus musculus GN=Cse1l PE=2 SV=1	sp|Q9ERK4|XPO2_MOUSE	110 kDa	0	5
Loricrin OS=Homo sapiens GN=LOR PE=1 SV=2	sp|P23490|LORI_HUMAN	26 kDa	0	5
RNA 3'-terminal phosphate cyclase OS=Mus musculus GN=Rtcd1 PE=2 SV=2	sp|Q9D7H3|RTC1_MOUSE	39 kDa	0	5
28S ribosomal protein S10, mitochondrial OS=Mus musculus GN=Mrps10 PE=2 SV=1	sp|Q80ZK0|RT10_MOUSE	19 kDa	4	2
Protein kinase C and casein kinase substrate in neurons protein 2 OS=Mus musculus (	sp|Q9WVE8|PACN2_MOUSE	56 kDa	0	5
Methylthioribose-1-phosphate isomerase OS=Mus musculus GN=Mri1 PE=2 SV=1	sp|Q9CQT1|MTNA_MOUSE	39 kDa	0	5
Abhydrolase domain-containing protein 1 OS=Mus musculus GN=Abhd1 PE=2 SV=2	sp|Q9QZC8|ABHD1_MOUSE	46 kDa	3	0
FACT complex subunit SSRP1 OS=Mus musculus GN=Ssrp1 PE=1 SV=2	sp|Q08943|SSRP1_MOUSE (+1)	81 kDa	3	0
39S ribosomal protein L17, mitochondrial OS=Mus musculus GN=Mrpl17 PE=1 SV=1	sp|Q9D8P4|RM17_MOUSE	20 kDa	3	0
Cytosol aminopeptidase OS=Mus musculus GN=Lap3 PE=1 SV=3	sp|Q9CPY7|AMPL_MOUSE	56 kDa	0	5
60S ribosomal protein L37 OS=Homo sapiens GN=RPL37 PE=1 SV=2	sp|P61927|RL37_HUMAN (+1)	11 kDa	3	0
Fatty acid-binding protein, heart OS=Mus musculus GN=Fabp3 PE=1 SV=5	sp|P11404|FABPH_MOUSE	15 kDa	0	6
Peptidyl-prolyl cis-trans isomerase H OS=Homo sapiens GN=PPIH PE=1 SV=1	sp|O43447|PPIH_HUMAN	19 kDa	3	0
Probable ATP-dependent RNA helicase YTHDC2 OS=Mus musculus GN=Ythdc2 PE=2 S	sp|B2RR83|YTDC2_MOUSE (+1)	161 kDa	3	0
ERI1 exoribonuclease 3 OS=Homo sapiens GN=ERI3 PE=1 SV=2	sp|O43414|ERI3_HUMAN (+1)	37 kDa	3	0
Dipeptidyl peptidase 3 OS=Mus musculus GN=Dpp3 PE=2 SV=2	sp|Q99KK7|DPP3_MOUSE	83 kDa	0	6

Mitochondrial 2-oxoglutarate/malate carrier protein OS=Homo sapiens GN=SLC25A1	sp|Q02978|M2OM_HUMAN	34 kDa	2	2
39S ribosomal protein L21, mitochondrial OS=Mus musculus GN=Mrpl21 PE=2 SV=1	sp|Q9D1N9|RM21_MOUSE	23 kDa	3	0
Lactotransferrin OS=Homo sapiens GN=LTF PE=1 SV=6	sp|P02788|TRFL_HUMAN	78 kDa	2	3
Small proline-rich protein 2E OS=Homo sapiens GN=SPRR2E PE=2 SV=2	sp|P22531|SPR2E_HUMAN (+4)	8 kDa	0	3
X-ray repair cross-complementing protein 5 OS=Mus musculus GN=Xrcc5 PE=2 SV=4	sp|P27641|XRCC5_MOUSE	83 kDa	3	0
SH3 and cysteine-rich domain-containing protein 3 OS=Mus musculus GN=Stac3 PE=2	sp|Q8BZ71|STAC3_MOUSE (+1)	41 kDa	2	2
Cat eye syndrome critical region protein 2 OS=Homo sapiens GN=CECR2 PE=1 SV=2	sp|Q9BXF3|CECR2_HUMAN	164 kDa	2	0
SLIT-ROBO Rho GTPase-activating protein 3 OS=Mus musculus GN=Srgap3 PE=1 SV=1	sp|Q812A2|SRGP2_MOUSE	124 kDa	2	0
Protein S100-A9 OS=Homo sapiens GN=S100A9 PE=1 SV=1	sp|P06702|S10A9_HUMAN	13 kDa	0	3
Probable ATP-dependent RNA helicase DDX6 OS=Homo sapiens GN=DDX6 PE=1 SV=2	sp|P26196|DDX6_HUMAN (+1)	54 kDa	3	2
Eukaryotic translation initiation factor 3 subunit H OS=Mus musculus GN=Eif3h PE=1	sp|Q91WK2|EIF3H_MOUSE	40 kDa	2	3
Septin-7 OS=Mus musculus GN=Sept7 PE=1 SV=1	sp|O55131|SEPT7_MOUSE (+1)	51 kDa	3	4
Methylcrotonoyl-CoA carboxylase beta chain, mitochondrial OS=Mus musculus GN=N	sp|Q3ULD5|MCCB_MOUSE	61 kDa	2	3
Dynactin subunit 2 OS=Mus musculus GN=Dctn2 PE=1 SV=3	sp|Q99KJ8|DCTN2_MOUSE	44 kDa	3	2
DnaJ homolog subfamily A member 4 OS=Mus musculus GN=Dnaja4 PE=2 SV=1	sp|Q9JMC3|DNJA4_MOUSE	45 kDa	3	4
Zyxin OS=Mus musculus GN=Zyx PE=1 SV=2	sp|Q62523|ZYX_MOUSE	61 kDa	2	3
Protein TFG OS=Homo sapiens GN=TFG PE=1 SV=2	sp|Q92734|TFG_HUMAN	43 kDa	2	3
Nuclear factor NF-kappa-B p100 subunit OS=Mus musculus GN=Nfkb2 PE=1 SV=1	sp|Q9WTK5|NFKB2_MOUSE	97 kDa	2	0
DNA replication licensing factor MCM5 OS=Mus musculus GN=Mcm5 PE=2 SV=1	sp|P49718|MCM5_MOUSE	82 kDa	3	2
Ataxin-10 OS=Mus musculus GN=Atxn10 PE=1 SV=2	sp|P28658|ATX10_MOUSE	54 kDa	3	2
Histidine triad nucleotide-binding protein 1 OS=Mus musculus GN=Hint1 PE=1 SV=3	sp|P70349|HINT1_MOUSE	14 kDa	2	3
Alpha-1-antitrypsin OS=Homo sapiens GN=SERPINA1 PE=1 SV=3	sp|P01009|A1AT_HUMAN	47 kDa	2	3
Monofunctional C1-tetrahydrofolate synthase, mitochondrial OS=Mus musculus GN=	sp|Q3V3R1|C1TM_MOUSE	106 kDa	4	2
Replication protein A 32 kDa subunit OS=Mus musculus GN=Rpa2 PE=1 SV=1	sp|Q62193|RFA2_MOUSE	30 kDa	3	2
Transmembrane emp24 domain-containing protein 2 OS=Homo sapiens GN=TMED2	sp|Q15363|TMED2_HUMAN (+1)	23 kDa	0	3
LSM domain-containing protein 1 OS=Mus musculus GN=Lsmd1 PE=2 SV=1	sp|Q9D2U5|LSMD1_MOUSE	13 kDa	0	4
Peptidyl-prolyl cis-trans isomerase FKBP1A OS=Mus musculus GN=Fkbp1a PE=2 SV=2	sp|P26883|FKB1A_MOUSE (+1)	12 kDa	0	4
Macrophage migration inhibitory factor OS=Mus musculus GN=Mif PE=1 SV=2	sp|P34884|MIF_MOUSE	13 kDa	0	4
Elongation factor 1-beta OS=Mus musculus GN=Eef1b PE=1 SV=5	sp|O70251|EF1B_MOUSE	25 kDa	2	3
Pregnancy zone protein OS=Homo sapiens GN=PZP PE=1 SV=4	sp|P20742|PZP_HUMAN	164 kDa	2	0
Succinyl-CoA ligase [GDP-forming] subunit beta, mitochondrial OS=Mus musculus GN	sp|Q9Z2I8|SUCB2_MOUSE	47 kDa	0	5
Hydroxyacyl-coenzyme A dehydrogenase, mitochondrial OS=Mus musculus GN=Hadh	sp|Q61425|HCDH_MOUSE	34 kDa	0	4
Heat shock protein HSP 90-alpha OS=Mus musculus GN=Hsp90aa1 PE=1 SV=4	sp|P07901|HS90A_MOUSE	85 kDa	0	55
Hepatocyte growth factor-regulated tyrosine kinase substrate OS=Mus musculus GN=	sp|Q99LI8|HGS_MOUSE	86 kDa	2	0
V-type proton ATPase subunit F OS=Mus musculus GN=Atp6v1f PE=1 SV=2	sp|Q9D1K2|VATF_MOUSE	13 kDa	0	4
Phosphatidylethanolamine-binding protein 1 OS=Mus musculus GN=Pebp1 PE=1 SV=	sp|P70296|PEBP1_MOUSE	21 kDa	0	4
NADH dehydrogenase [ubiquinone] 1 alpha subcomplex subunit 8 OS=Mus musculus	sp|Q9DCJ5|NDUA8_MOUSE	20 kDa	0	4
Translocator protein OS=Mus musculus GN=Tspo PE=2 SV=1	sp|P50637|TSPO_MOUSE	19 kDa	0	3
Dynamin-1-like protein OS=Homo sapiens GN=DNM1L PE=1 SV=2	sp|O00429|DNM1L_HUMAN (+1)	82 kDa	0	3
S-formylglutathione hydrolase OS=Mus musculus GN=Esd PE=2 SV=1	sp|Q9R0P3|ESTD_MOUSE	31 kDa	0	3
Calcium/calmodulin-dependent protein kinase type II subunit gamma OS=Homo sapi	sp|Q13555|KCC2G_HUMAN (+1)	63 kDa	0	4
Golgin subfamily B member 1 OS=Homo sapiens GN=GOLGB1 PE=1 SV=2	sp|Q14789|GOGB1_HUMAN	376 kDa	2	0
Proto-oncogene tyrosine-protein kinase Yes OS=Homo sapiens GN=YES1 PE=1 SV=3	sp|P07947|YES_HUMAN (+1)	61 kDa	2	0
Myoglobin OS=Mus musculus GN=Mb PE=1 SV=3	sp|P04247|MYG_MOUSE	17 kDa	0	4
Acyl-coenzyme A thioesterase 13 OS=Mus musculus GN=Acot13 PE=1 SV=1	sp|Q9CQR4|ACO13_MOUSE	15 kDa	0	4
NADH dehydrogenase [ubiquinone] flavoprotein 1, mitochondrial OS=Mus musculus	sp|Q91YT0|NDUV1_MOUSE	51 kDa	0	4
Lipase maturation factor 2 OS=Mus musculus GN=Lmf2 PE=2 SV=1	sp|Q8C3X8|LMF2_MOUSE	80 kDa	2	0
Malate dehydrogenase, cytoplasmic OS=Mus musculus GN=Mdh1 PE=1 SV=3	sp|P14152|MDHC_MOUSE	37 kDa	0	5
Macrophage-capping protein OS=Mus musculus GN=Capg PE=1 SV=2	sp|P24452|CAPG_MOUSE	39 kDa	0	5
Medium-chain specific acyl-CoA dehydrogenase, mitochondrial OS=Mus musculus GN	sp|P45952|ACADM_MOUSE	46 kDa	0	5
26S proteasome non-ATPase regulatory subunit 6 OS=Homo sapiens GN=PSMD6 PE=	sp|Q15008|PSMD6_HUMAN (+1)	46 kDa	0	5
E3 ubiquitin-protein ligase TRIM63 OS=Mus musculus GN=Trim63 PE=1 SV=1	sp|Q38HM4|TRI63_MOUSE	39 kDa	0	11
UDP-N-acetylhexosamine pyrophosphorylase-like protein 1 OS=Mus musculus GN=U	sp|Q3TW96|UAP1L_MOUSE	57 kDa	0	5
Myosin-VI OS=Mus musculus GN=Myo6 PE=1 SV=1	sp|Q64331|MYO6_MOUSE	146 kDa	0	8
Tropomyosin alpha-4 chain OS=Mus musculus GN=Tpm4 PE=2 SV=3	sp|Q6IRU2|TPM4_MOUSE	28 kDa	0	10
Transportin-1 OS=Mus musculus GN=Tnpo1 PE=1 SV=2	sp|Q8BFY9|TNPO1_MOUSE (+1)	102 kDa	0	5
Ran GTPase-activating protein 1 OS=Mus musculus GN=Rangap1 PE=1 SV=2	sp|P46061|RAGP1_MOUSE	64 kDa	2	0
NADH dehydrogenase [ubiquinone] 1 alpha subcomplex subunit 6 OS=Mus musculus	sp|Q9CQZ5|NDUA6_MOUSE	15 kDa	0	5
Cytochrome b-c1 complex subunit 1, mitochondrial OS=Mus musculus GN=Uqcrc1 PE	sp|Q9CZ13|QCR1_MOUSE	53 kDa	0	6
14 kDa phosphohistidine phosphatase OS=Mus musculus GN=Phpt1 PE=1 SV=1	sp|Q9DAK9|PHP14_MOUSE	14 kDa	0	5
NADH dehydrogenase [ubiquinone] 1 beta subcomplex subunit 5, mitochondrial OS=	sp|Q9CQH3|NDUB5_MOUSE	22 kDa	0	5
Rab3 GTPase-activating protein catalytic subunit OS=Mus musculus GN=Rab3gap1 PE	sp|Q80UJ7|RB3GP_MOUSE	110 kDa	2	0
Transmembrane protein 33 OS=Homo sapiens GN=TMEM33 PE=1 SV=2	sp|P57088|TMM33_HUMAN (+1)	28 kDa	2	0
UPF0556 protein C19orf10 homolog OS=Mus musculus GN=D17Wsu104e PE=2 SV=1	sp|Q9CPT4|CS010_MOUSE	18 kDa	0	5
Protein ERGIC-53 OS=Mus musculus GN=Lman1 PE=2 SV=1	sp|Q9D0F3|LMAN1_MOUSE	58 kDa	0	5
Protein disulfide-isomerase A4 OS=Mus musculus GN=Pdia4 PE=1 SV=3	sp|P08003|PDIA4_MOUSE	72 kDa	0	5
NADH dehydrogenase [ubiquinone] iron-sulfur protein 3, mitochondrial OS=Mus mus	sp|Q9DCT2|NDUS3_MOUSE (+1)	30 kDa	0	2
Dynamin-like 120 kDa protein, mitochondrial OS=Homo sapiens GN=OPA1 PE=1 SV=3	sp|O60313|OPA1_HUMAN (+1)	112 kDa	2	0
Smoothelin OS=Mus musculus GN=Smtn PE=2 SV=2	sp|Q921U8|SMTN_MOUSE	100 kDa	0	2
Ras-related protein Rab-21 OS=Mus musculus GN=Rab21 PE=1 SV=4	sp|P35282|RAB21_MOUSE (+1)	24 kDa	3	2
Cell division control protein 42 homolog OS=Mus musculus GN=Cdc42 PE=1 SV=2	sp|P60766|CDC42_MOUSE (+1)	21 kDa	2	2
Dynein light chain roadblock-type 1 OS=Mus musculus GN=Dynlrb1 PE=1 SV=3	sp|P62627|DLRB1_MOUSE	11 kDa	2	2
39S ribosomal protein L55, mitochondrial OS=Homo sapiens GN=MRPL55 PE=1 SV=1	sp|Q7Z7F7|RM55_HUMAN (+1)	15 kDa	2	2
Nicalin OS=Mus musculus GN=Ncln PE=2 SV=2	sp|Q8VCM8|NCLN_MOUSE	63 kDa	2	2
Hypoxanthine-guanine phosphoribosyltransferase OS=Homo sapiens GN=HPRT1 PE=	sp|P00492|HPRT_HUMAN (+1)	25 kDa	2	2
Calcyclin-binding protein OS=Mus musculus GN=Cacybp PE=1 SV=1	sp|Q9CXW3|CYBP_MOUSE	27 kDa	2	2
Upstream-binding protein 1 OS=Mus musculus GN=Ubp1 PE=1 SV=1	sp|Q811S7|UBIP1_MOUSE (+1)	60 kDa	3	4

Acetoacetyl-CoA synthetase OS=Mus musculus GN=Aacs PE=1 SV=1	sp|Q9D2R0|AACS_MOUSE	75 kDa	2	3
Stomatin-like protein 2 OS=Mus musculus GN=Stoml2 PE=1 SV=1	sp|Q99JB2|STML2_MOUSE	38 kDa	2	0
AP-2 complex subunit sigma OS=Homo sapiens GN=AP2S1 PE=1 SV=2	sp|P53680|AP2S1_HUMAN (+1)	17 kDa	2	0
Transforming protein RhoA OS=Homo sapiens GN=RHOA PE=1 SV=1	sp|P61586|RHOA_HUMAN (+1)	22 kDa	0	3
Vesicle-associated membrane protein-associated protein A OS=Homo sapiens GN=VA	sp|Q9P0L0|VAPA_HUMAN (+1)	28 kDa	0	3
Cyclic AMP-responsive element-binding protein 1 OS=Homo sapiens GN=CREB1 PE=1	sp|P16220|CREB1_HUMAN (+1)	37 kDa	0	3
Voltage-dependent calcium channel subunit alpha-2/delta-1 OS=Mus musculus GN=C	sp|O08532|CA2D1_MOUSE (+1)	125 kDa	2	2
Serotransferrin OS=Homo sapiens GN=TF PE=1 SV=3	sp|P02787|TRFE_HUMAN	77 kDa	2	7
Sorting nexin-6 OS=Mus musculus GN=Snx6 PE=1 SV=2	sp|Q6P8X1|SNX6_MOUSE (+1)	47 kDa	2	0
ATP synthase subunit delta, mitochondrial OS=Mus musculus GN=Atp5d PE=1 SV=1	sp|Q9D3D9|ATPD_MOUSE	18 kDa	0	3
Trafficking protein particle complex subunit 6B OS=Mus musculus GN=Trappc6b PE=2	sp|Q9D289|TPC6B_MOUSE	18 kDa	0	3
COP9 signalosome complex subunit 5 OS=Mus musculus GN=Cops5 PE=1 SV=3	sp|O35864|CSN5_MOUSE (+1)	38 kDa	2	0
Rapamycin-insensitive companion of mTOR OS=Mus musculus GN=Rictor PE=1 SV=2	sp|Q6QI06|RICTR_MOUSE (+1)	192 kDa	2	0
Plastin-3 OS=Homo sapiens GN=PLS3 PE=1 SV=4	sp|P13797|PLST_HUMAN (+1)	71 kDa	0	2
Down syndrome critical region protein 3 homolog OS=Mus musculus GN=Dscr3 PE=2	sp|O35075|DSCR3_MOUSE	33 kDa	2	0
Translational activator GCN1 OS=Homo sapiens GN=GCN1L1 PE=1 SV=6	sp|Q92616|GCN1L_HUMAN	293 kDa	0	3
Long-chain-fatty-acid--CoA ligase 4 OS=Homo sapiens GN=ACSL4 PE=1 SV=2	sp|O60488|ACSL4_HUMAN (+1)	79 kDa	0	4
Microsomal glutathione S-transferase 1 OS=Mus musculus GN=Mgst1 PE=1 SV=3	sp|Q91VS7|MGST1_MOUSE	18 kDa	2	2
Microtubule-associated protein 1S OS=Mus musculus GN=Map1s PE=1 SV=2	sp|Q8C052|MAP1S_MOUSE	103 kDa	2	0
Eukaryotic translation initiation factor 4E OS=Homo sapiens GN=EIF4E PE=1 SV=2	sp|P06730|IF4E_HUMAN (+1)	25 kDa	2	2
Glutamine synthetase OS=Mus musculus GN=Glul PE=1 SV=6	sp|P15105|GLNA_MOUSE	42 kDa	0	3
Prostacyclin synthase OS=Mus musculus GN=Ptgis PE=2 SV=1	sp|O35074|PTGIS_MOUSE	57 kDa	0	3
Caveolin-1 OS=Mus musculus GN=Cav1 PE=1 SV=1	sp|P49817|CAV1_MOUSE	21 kDa	0	5
Coatomer subunit zeta-2 OS=Mus musculus GN=Copz2 PE=2 SV=1	sp|Q9JHH9|COPZ2_MOUSE	23 kDa	2	0
Nicastrin OS=Mus musculus GN=Ncstn PE=1 SV=3	sp|P57716|NICA_MOUSE	78 kDa	0	3
Signal recognition particle 54 kDa protein OS=Mus musculus GN=Srp54 PE=1 SV=2	sp|P14576|SRP54_MOUSE (+1)	56 kDa	0	2
DCN1-like protein 5 OS=Mus musculus GN=Dcun1d5 PE=2 SV=1	sp|Q9CXV9|DCNL5_MOUSE	28 kDa	0	2
Sorting nexin-3 OS=Homo sapiens GN=SNX3 PE=1 SV=3	sp|O60493|SNX3_HUMAN (+1)	19 kDa	2	0
Myosin-binding protein H-like OS=Homo sapiens GN=MYBPHL PE=2 SV=2	sp|A2RUH7|MBPHL_HUMAN (+1)	39 kDa	2	0
Cytochrome c oxidase subunit 5A, mitochondrial OS=Homo sapiens GN=COX5A PE=1	sp|P20674|COX5A_HUMAN (+1)	17 kDa	0	2
Protein DJ-1 OS=Mus musculus GN=Park7 PE=1 SV=1	sp|Q99LX0|PARK7_MOUSE	20 kDa	0	3
Synaptopodin-2 OS=Mus musculus GN=Synpo2 PE=1 SV=2	sp|Q91YE8|SYNP2_MOUSE	117 kDa	2	0
COP9 signalosome complex subunit 2 OS=Homo sapiens GN=COPS2 PE=1 SV=1	sp|P61201|CSN2_HUMAN (+1)	52 kDa	2	0
Retinal dehydrogenase 1 OS=Mus musculus GN=Aldh1a1 PE=1 SV=5	sp|P24549|AL1A1_MOUSE	54 kDa	0	4
Laminin subunit gamma-1 OS=Mus musculus GN=Lamc1 PE=1 SV=2	sp|P02468|LAMC1_MOUSE	177 kDa	2	0
Glutamate-rich WD repeat-containing protein 1 OS=Mus musculus GN=Grwd1 PE=2 S	sp|Q810D6|GRWD1_MOUSE	49 kDa	2	0
Thyroid receptor-interacting protein 11 OS=Homo sapiens GN=TRIP11 PE=1 SV=3	sp|Q15643|TRIPB_HUMAN	228 kDa	2	0
TBC1 domain family member 1 OS=Mus musculus GN=Tbc1d1 PE=2 SV=3	sp|Q60949|TBCD1_MOUSE	142 kDa	2	0
Protein pelota homolog OS=Mus musculus GN=Pelo PE=2 SV=3	sp|Q80X73|PELO_MOUSE (+1)	43 kDa	2	0
DnaJ homolog subfamily B member 4 OS=Mus musculus GN=Dnajb4 PE=2 SV=1	sp|Q9D832|DNJB4_MOUSE	38 kDa	2	0
Aquaporin-5 OS=Mus musculus GN=Aqp5 PE=2 SV=1	sp|Q9WTY4|AQP5_MOUSE	28 kDa	2	0
Tropomyosin alpha-4 chain OS=Homo sapiens GN=TPM4 PE=1 SV=3	sp|P67936|TPM4_HUMAN	29 kDa	0	16
Vesicle-fusing ATPase OS=Homo sapiens GN=NSF PE=1 SV=3	sp|P46459|NSF_HUMAN (+1)	83 kDa	0	2
Importin-5 OS=Homo sapiens GN=IPO5 PE=1 SV=4	sp|O00410|IPO5_HUMAN (+1)	124 kDa	0	3
Protein KIAA1967 OS=Homo sapiens GN=KIAA1967 PE=1 SV=2	sp|Q8N163|K1967_HUMAN (+1)	103 kDa	2	0
Thioredoxin OS=Mus musculus GN=Txn PE=1 SV=3	sp|P10639|THIO_MOUSE	12 kDa	0	4
Inosine-5'-monophosphate dehydrogenase 2 OS=Homo sapiens GN=IMPDH2 PE=1 SV	sp|P12268|IMDH2_HUMAN (+1)	56 kDa	0	4
Microtubule-associated protein 4 OS=Mus musculus GN=Map4 PE=1 SV=3	sp|P27546|MAP4_MOUSE	117 kDa	2	0
Serine/threonine-protein kinase 17B OS=Mus musculus GN=Stk17b PE=2 SV=1	sp|Q8BG48|ST17B_MOUSE	42 kDa	2	0
Importin-4 OS=Mus musculus GN=Ipo4 PE=1 SV=1	sp|Q8VI75|IPO4_MOUSE	119 kDa	0	4
Trafficking protein particle complex subunit 2-like protein OS=Mus musculus GN=Tra	sp|Q9JME7|TPC2L_MOUSE	16 kDa	2	0
Abl interactor 1 OS=Mus musculus GN=Abi1 PE=1 SV=3	sp|Q8CBW3|ABI1_MOUSE (+1)	52 kDa	2	0
COP9 signalosome complex subunit 8 OS=Mus musculus GN=Cops8 PE=1 SV=1	sp|Q8VBV7|CSN8_MOUSE	23 kDa	2	0
Isocitrate dehydrogenase [NADP] cytoplasmic OS=Mus musculus GN=Idh1 PE=1 SV=2	sp|O88844|IDHC_MOUSE	47 kDa	0	4
Rho guanine nucleotide exchange factor 10-like protein OS=Mus musculus GN=Arhge	sp|A2AWP8|ARGAL_MOUSE (+1)	140 kDa	2	0
Brain protein 44 OS=Mus musculus GN=Brp44 PE=1 SV=1	sp|Q9D023|BR44_MOUSE	14 kDa	0	4
Succinyl-CoA:3-ketoacid-coenzyme A transferase 1, mitochondrial OS=Mus musculus	sp|Q9D0K2|SCOT1_MOUSE	56 kDa	0	4
Pyruvate dehydrogenase protein X component, mitochondrial OS=Mus musculus GN	sp|Q8BKZ9|ODPX_MOUSE	54 kDa	0	4
Ubiquitin-like protein 4A OS=Mus musculus GN=Ubl4a PE=2 SV=1	sp|P21126|UBL4A_MOUSE	18 kDa	0	4
Striatin-3 OS=Mus musculus GN=Strn3 PE=1 SV=1	sp|Q9ERG2|STRN3_MOUSE	87 kDa	0	4
Non-specific lipid-transfer protein OS=Mus musculus GN=Scp2 PE=1 SV=3	sp|P32020|NLTP_MOUSE	59 kDa	0	4
Cytochrome c oxidase subunit 4 isoform 1, mitochondrial OS=Mus musculus GN=Cox	sp|P19783|COX41_MOUSE	20 kDa	0	4
UPF0366 protein C11orf67 homolog OS=Mus musculus PE=2 SV=1	sp|Q8R0P4|CK067_MOUSE	13 kDa	0	4
Luc7-like protein 3 OS=Homo sapiens GN=LUC7L3 PE=1 SV=2	sp|O95232|LC7L3_HUMAN (+1)	51 kDa	2	0
Cystatin-B OS=Mus musculus GN=Cstb PE=1 SV=1	sp|Q62426|CYTB_MOUSE	11 kDa	0	4
DnaJ homolog subfamily C member 3 OS=Mus musculus GN=Dnajc3 PE=1 SV=1	sp|Q91YW3|DNJC3_MOUSE	57 kDa	0	4
Bis(5'-nucleosyl)-tetraphosphatase [asymmetrical] OS=Mus musculus GN=Nudt2 PE=1	sp|P56380|AP4A_MOUSE	17 kDa	0	4
Complement C1q tumor necrosis factor-related protein 1 OS=Mus musculus GN=C1q	sp|Q9QXP7|C1QT1_MOUSE	32 kDa	2	0
Replication protein A 14 kDa subunit OS=Mus musculus GN=Rpa3 PE=2 SV=1	sp|Q9CQ71|RFA3_MOUSE	14 kDa	0	4
COP9 signalosome complex subunit 1 OS=Homo sapiens GN=GPS1 PE=1 SV=4	sp|Q13098|CSN1_HUMAN (+1)	56 kDa	0	4
Selenoprotein T OS=Homo sapiens GN=SELT PE=2 SV=2	sp|P62341|SELT_HUMAN (+1)	22 kDa	0	4
Glia maturation factor beta OS=Mus musculus GN=Gmfb PE=1 SV=3	sp|Q9CQI3|GMFB_MOUSE	17 kDa	0	4
ATP synthase subunit a OS=Mus musculus GN=Mtatp6 PE=2 SV=1	sp|P00848|ATP6_MOUSE	25 kDa	0	4
DnaJ homolog subfamily B member 5 OS=Homo sapiens GN=DNAJB5 PE=2 SV=1	sp|O75953|DNJB5_HUMAN (+1)	39 kDa	2	0
39S ribosomal protein L30, mitochondrial OS=Mus musculus GN=Mrpl30 PE=2 SV=1	sp|Q9D7N6|RM30_MOUSE	18 kDa	0	4
Uncharacterized protein C2orf47 homolog, mitochondrial OS=Mus musculus PE=2 SV	sp|Q8BHE8|CB047_MOUSE	33 kDa	2	0
NF-kappa-B inhibitor-interacting Ras-like protein 2 OS=Mus musculus GN=Nkiras2 PE	sp|Q9CR56|KBRS2_MOUSE	21 kDa	2	0

Brain protein 44-like protein OS=Mus musculus GN=Brp44l PE=1 SV=1	sp|P63030|BR44L_MOUSE	12 kDa	0	4
Cytochrome c oxidase assembly protein COX16 homolog, mitochondrial OS=Mus mus	sp|Q9CR63|COX16_MOUSE	12 kDa	0	4
Uncharacterized protein C6orf125 homolog OS=Mus musculus PE=2 SV=1	sp|Q9CQY6|CF125_MOUSE	16 kDa	0	4
Uncharacterized protein C17orf85 OS=Homo sapiens GN=C17orf85 PE=1 SV=2	sp|Q53F19|CQ085_HUMAN (+1)	71 kDa	0	2
Ubiquitin carboxyl-terminal hydrolase 34 OS=Mus musculus GN=Usp34 PE=1 SV=3	sp|Q6ZQ93|UBP34_MOUSE (+1)	408 kDa	0	2
Cathepsin D OS=Mus musculus GN=Ctsd PE=1 SV=1	sp|P18242|CATD_MOUSE	45 kDa	0	2
ADP-ribosylation factor 2 OS=Mus musculus GN=Arf2 PE=1 SV=2	sp|Q8BSL7|ARF2_MOUSE	21 kDa	0	15
Dolichyl-diphosphooligosaccharide--protein glycosyltransferase subunit DAD1 OS=Ho	sp|P61803|DAD1_HUMAN (+1)	12 kDa	0	2
Iron-sulfur cluster assembly 1 homolog, mitochondrial OS=Homo sapiens GN=ISCA1 F	sp|Q9BUE6|ISCA1_HUMAN	14 kDa	0	2
Synaptogyrin-2 OS=Mus musculus GN=Syngr2 PE=2 SV=1	sp|O55101|SNG2_MOUSE	25 kDa	2	0
Synaptophysin-like protein 1 OS=Mus musculus GN=Sypl1 PE=1 SV=2	sp|O09117|SYPL1_MOUSE	29 kDa	0	2
Receptor expression-enhancing protein 5 OS=Mus musculus GN=Reep5 PE=1 SV=1	sp|Q60870|REEP5_MOUSE	21 kDa	0	2
Ankyrin repeat domain-containing protein 40 OS=Mus musculus GN=Ankrd40 PE=1 S'	sp|Q5SUE8|ANR40_MOUSE	41 kDa	2	0
Hydroxymethylglutaryl-CoA synthase, mitochondrial OS=Mus musculus GN=Hmgcs2 F	sp|P54869|HMCS2_MOUSE	57 kDa	0	2
Adipocyte enhancer-binding protein 1 OS=Mus musculus GN=Aebp1 PE=1 SV=1	sp|Q640N1|AEBP1_MOUSE	128 kDa	2	0
Peptidyl-prolyl cis-trans isomerase FKBP10 OS=Homo sapiens GN=FKBP10 PE=1 SV=1	sp|Q96AY3|FKB10_HUMAN	64 kDa	0	2
DnaJ homolog subfamily B member 2 OS=Mus musculus GN=Dnajb2 PE=2 SV=2	sp|Q9QYI5|DNJB2_MOUSE	31 kDa	0	3
DnaJ homolog subfamily C member 21 OS=Homo sapiens GN=DNAJC21 PE=1 SV=2	sp|Q5F1R6|DJC21_HUMAN	62 kDa	2	0
Ran-binding protein 10 OS=Mus musculus GN=Ranbp10 PE=1 SV=1	sp|Q6VN19|RBP10_MOUSE (+1)	70 kDa	2	0
Tubulin-specific chaperone E OS=Mus musculus GN=Tbce PE=1 SV=1	sp|Q8CIV8|TBCE_MOUSE	59 kDa	2	0
DnaJ homolog subfamily C member 9 OS=Mus musculus GN=Dnajc9 PE=2 SV=2	sp|Q91WN1|DNJC9_MOUSE	30 kDa	2	0
Transcriptional enhancer factor TEF-3 OS=Mus musculus GN=Tead4 PE=1 SV=2	sp|Q62296|TEAD4_MOUSE	48 kDa	0	2
Nitric oxide synthase-interacting protein OS=Mus musculus GN=Nosip PE=2 SV=1	sp|Q9D6T0|NOSIP_MOUSE	33 kDa	2	0
Recombining binding protein suppressor of hairless OS=Mus musculus GN=Rbpj PE=1	sp|P31266|SUH_MOUSE (+1)	59 kDa	0	2
Phosphatidylinositol-5-phosphate 4-kinase type-2 alpha OS=Mus musculus GN=Pip4k	sp|O70172|PI42A_MOUSE	46 kDa	2	0
Collagen alpha-1(XVIII) chain OS=Mus musculus GN=Col18a1 PE=1 SV=4	sp|P39061|COIA1_MOUSE	182 kDa	0	2
Mannosyl-oligosaccharide glucosidase OS=Mus musculus GN=Mogs PE=2 SV=1	sp|Q80UM7|MOGS_MOUSE	92 kDa	0	2
von Willebrand factor A domain-containing protein 5A OS=Mus musculus GN=Vwa5a	sp|Q99KC8|VMA5A_MOUSE	87 kDa	0	2
F-box/WD repeat-containing protein 8 OS=Mus musculus GN=Fbxw8 PE=1 SV=2	sp|Q8BIA4|FBXW8_MOUSE	68 kDa	0	2
CUGBP Elav-like family member 2 OS=Homo sapiens GN=CELF2 PE=1 SV=1	sp|O95319|CELF2_HUMAN (+1)	54 kDa	0	2
Sorting nexin-24 OS=Mus musculus GN=Snx24 PE=2 SV=1	sp|Q9CRB0|SNX24_MOUSE (+1)	20 kDa	2	0
Proteasome subunit alpha type-1 OS=Homo sapiens GN=PSMA1 PE=1 SV=1	sp|P25786|PSA1_HUMAN (+1)	30 kDa	0	2
GTP-binding protein Rheb OS=Homo sapiens GN=RHEB PE=1 SV=1	sp|Q15382|RHEB_HUMAN (+1)	20 kDa	0	2
Protein disulfide-isomerase TMX3 OS=Homo sapiens GN=TMX3 PE=1 SV=2	sp|Q96JJ7|TMX3_HUMAN	52 kDa	0	4
Derlin-1 OS=Mus musculus GN=Derl1 PE=2 SV=1	sp|Q99J56|DERL1_MOUSE (+1)	29 kDa	0	2
Late cornified envelope protein 1B OS=Homo sapiens GN=LCE1B PE=2 SV=1	sp|Q5T7P3|LCE1B_HUMAN	12 kDa	0	2
Cytochrome b-c1 complex subunit Rieske, mitochondrial OS=Mus musculus GN=Uqcr	sp|Q9CR68|UCRI_MOUSE	29 kDa	0	2
Tripartite motif-containing protein 26 OS=Mus musculus GN=Trim26 PE=2 SV=3	sp|Q99PN3|TRI26_MOUSE	63 kDa	2	0
Caprin-1 OS=Homo sapiens GN=CAPRIN1 PE=1 SV=2	sp|Q14444|CAPR1_HUMAN (+1)	78 kDa	2	0
DEP domain-containing mTOR-interacting protein OS=Mus musculus GN=Deptor PE=	sp|Q570Y9|DPTOR_MOUSE (+1)	46 kDa	2	0
Sphingomyelin phosphodiesterase 2 OS=Mus musculus GN=Smpd2 PE=1 SV=1	sp|O70572|NSMA_MOUSE	47 kDa	2	0
Serine/threonine-protein kinase Nek7 OS=Homo sapiens GN=NEK7 PE=1 SV=1	sp|Q8TDX7|NEK7_HUMAN (+1)	35 kDa	2	0
FAS-associated factor 2 OS=Mus musculus GN=Faf2 PE=2 SV=2	sp|Q3TDN2|FAF2_MOUSE (+1)	52 kDa	0	2
Protein FAM105B OS=Mus musculus GN=Fam105b PE=2 SV=1	sp|Q3UCV8|F105B_MOUSE	40 kDa	2	0
PERQ amino acid-rich with GYF domain-containing protein 2 OS=Mus musculus GN=G	sp|Q6Y7W8|PERQ2_MOUSE	149 kDa	0	2
Uncharacterized protein C7orf30 homolog OS=Mus musculus PE=2 SV=1	sp|Q9CWV0|CG030_MOUSE	26 kDa	2	0
Cyclin-dependent kinase inhibitor 1 OS=Mus musculus GN=Cdkn1a PE=1 SV=4	sp|P39689|CDN1A_MOUSE	18 kDa	2	0
Bleomycin hydrolase OS=Mus musculus GN=Blmh PE=2 SV=1	sp|Q8R016|BLMH_MOUSE	53 kDa	0	2
Proteasome subunit beta type-2 OS=Homo sapiens GN=PSMB2 PE=1 SV=1	sp|P49721|PSB2_HUMAN (+1)	23 kDa	0	3
UPF0598 protein C8orf82 OS=Homo sapiens GN=C8orf82 PE=1 SV=2	sp|Q6P1X6|CH082_HUMAN (+1)	24 kDa	2	0
RAF proto-oncogene serine/threonine-protein kinase OS=Homo sapiens GN=RAF1 PE	sp|P04049|RAF1_HUMAN (+1)	73 kDa	2	0
Long-chain-fatty-acid--CoA ligase 1 OS=Mus musculus GN=Acsl1 PE=1 SV=2	sp|P41216|ACSL1_MOUSE	78 kDa	0	2
DnaJ homolog subfamily C member 16 OS=Mus musculus GN=Dnajc16 PE=1 SV=2	sp|Q80TN4|DJC16_MOUSE	89 kDa	2	0
Protein FAM40A OS=Mus musculus GN=Fam40a PE=1 SV=2	sp|Q8C079|FA40A_MOUSE	96 kDa	2	0
Eukaryotic translation initiation factor 2-alpha kinase 4 OS=Homo sapiens GN=EIF2AK	sp|Q9P2K8|E2AK4_HUMAN	187 kDa	0	2
Superkiller viralicidic activity 2-like 2 OS=Homo sapiens GN=SKIV2L2 PE=1 SV=3	sp|P42285|SK2L2_HUMAN (+1)	118 kDa	2	0
Protein eyes shut homolog OS=Homo sapiens GN=EYS PE=1 SV=4	sp|Q5T1H1|EYS_HUMAN	351 kDa	2	0
Ral GTPase-activating protein subunit alpha-1 OS=Homo sapiens GN=RALGAPA1 PE=1	sp|Q6GYQ0|RGPA1_HUMAN	230 kDa	2	0
Laminin subunit beta-1 OS=Mus musculus GN=Lamb1 PE=1 SV=3	sp|P02469|LAMB1_MOUSE	197 kDa	2	0
SH3 domain-binding glutamic acid-rich-like protein OS=Homo sapiens GN=SH3BGRL F	sp|O75368|SH3L1_HUMAN	13 kDa	0	2
Dynein heavy chain 12, axonemal OS=Homo sapiens GN=DNAH12 PE=1 SV=2	sp|Q6ZR08|DYH12_HUMAN	357 kDa	2	0
Alpha-2-macroglobulin OS=Mus musculus GN=A2m PE=1 SV=3	sp|Q61838|A2M_MOUSE	166 kDa	2	0
Cysteine and glycine-rich protein 3 OS=Mus musculus GN=Csrp3 PE=1 SV=1	sp|P50462|CSRP3_MOUSE	21 kDa	0	2
Proteasome subunit beta type-1 OS=Mus musculus GN=Psmb1 PE=1 SV=1	sp|O09061|PSB1_MOUSE (+1)	26 kDa	0	2
Large neutral amino acids transporter small subunit 1 OS=Mus musculus GN=Slc7a5 F	sp|Q9Z127|LAT1_MOUSE	56 kDa	2	0
Telomerase protein component 1 OS=Mus musculus GN=Tep1 PE=1 SV=1	sp|P97499|TEP1_MOUSE	291 kDa	2	0
Proteasome subunit alpha type-2 OS=Homo sapiens GN=PSMA2 PE=1 SV=2	sp|P25787|PSA2_HUMAN (+1)	26 kDa	0	3
Prohibitin OS=Homo sapiens GN=PHB PE=1 SV=1	sp|P35232|PHB_HUMAN (+1)	30 kDa	0	3
NEDD8-activating enzyme E1 regulatory subunit OS=Mus musculus GN=Nae1 PE=2 SV	sp|Q8VBW6|ULA1_MOUSE	60 kDa	0	3
Histone H2A.Z OS=Homo sapiens GN=H2AFZ PE=1 SV=2	sp|P0C0S5|H2AZ_HUMAN (+3)	14 kDa	2	0
Myosin regulatory light polypeptide 9 OS=Mus musculus GN=Myl9 PE=1 SV=3	sp|Q9CQ19|MYL9_MOUSE	20 kDa	0	18
Cysteinyl-tRNA synthetase, cytoplasmic OS=Mus musculus GN=Cars PE=1 SV=2	sp|Q9ER72|SYCC_MOUSE	95 kDa	0	3
Sterol-4-alpha-carboxylate 3-dehydrogenase, decarboxylating OS=Mus musculus GN=	sp|Q9R1J0|NSDHL_MOUSE	41 kDa	0	3
NADPH--cytochrome P450 reductase OS=Homo sapiens GN=POR PE=1 SV=2	sp|P16435|NCPR_HUMAN (+1)	77 kDa	0	3
Pericentriolar material 1 protein OS=Homo sapiens GN=PCM1 PE=1 SV=4	sp|Q15154|PCM1_HUMAN	229 kDa	2	0
26S proteasome non-ATPase regulatory subunit 5 OS=Mus musculus GN=Psmd5 PE=2	sp|Q8BJY1|PSMD5_MOUSE	56 kDa	2	0
Coronin-1B OS=Homo sapiens GN=CORO1B PE=1 SV=1	sp|Q9BR76|COR1B_HUMAN (+1)	54 kDa	0	4

Erythrocyte band 7 integral membrane protein OS=Mus musculus GN=Stom PE=1 SV=	sp|P54116|STOM_MOUSE	31 kDa	2	0
Neurobeachin-like protein 2 OS=Mus musculus GN=Nbeal2 PE=2 SV=2	sp|Q6ZQA0|NBEL2_MOUSE	302 kDa	2	0
Obg-like ATPase 1 OS=Mus musculus GN=Ola1 PE=1 SV=1	sp|Q9CZ30|OLA1_MOUSE (+1)	45 kDa	0	3
Inactive dual specificity phosphatase 27 OS=Mus musculus GN=Dusp27 PE=2 SV=1	sp|Q148W8|DUS27_MOUSE	129 kDa	2	0
28S ribosomal protein S30, mitochondrial OS=Mus musculus GN=Mrps30 PE=2 SV=1	sp|Q9D0G0|RT30_MOUSE	50 kDa	2	0
Coiled-coil domain-containing protein 56 OS=Mus musculus GN=Ccdc56 PE=2 SV=1	sp|Q9D2R6|CCD56_MOUSE	12 kDa	0	3
Endoribonuclease Dicer OS=Mus musculus GN=Dicer1 PE=1 SV=3	sp|Q8R418|DICER_MOUSE (+1)	217 kDa	2	0
Caspase-3 OS=Mus musculus GN=Casp3 PE=1 SV=1	sp|P70677|CASP3_MOUSE	31 kDa	2	0
Signal recognition particle 72 kDa protein OS=Homo sapiens GN=SRP72 PE=1 SV=3	sp|O76094|SRP72_HUMAN	75 kDa	2	0
Histidine triad nucleotide-binding protein 2, mitochondrial OS=Mus musculus GN=Hi	sp|Q9D0S9|HINT2_MOUSE	17 kDa	0	3
Isovaleryl-CoA dehydrogenase, mitochondrial OS=Mus musculus GN=Ivd PE=1 SV=1	sp|Q9JHI5|IVD_MOUSE	46 kDa	0	3
BRCA1-A complex subunit BRE OS=Mus musculus GN=Bre PE=1 SV=2	sp|Q8K3W0|BRE_MOUSE (+1)	44 kDa	2	0
Fatty acid-binding protein, epidermal OS=Homo sapiens GN=FABP5 PE=1 SV=3	sp|Q01469|FABP5_HUMAN	15 kDa	0	3
ORM1-like protein 3 OS=Homo sapiens GN=ORMDL3 PE=1 SV=1	sp|Q8N138|ORML3_HUMAN (+1)	17 kDa	0	3
RNA-binding protein 14 OS=Mus musculus GN=Rbm14 PE=1 SV=1	sp|Q8C2Q3|RBM14_MOUSE	69 kDa	2	0
Alpha-parvin OS=Mus musculus GN=Parva PE=1 SV=1	sp|Q9EPC1|PARVA_MOUSE (+1)	42 kDa	2	0
Calcium-binding mitochondrial carrier protein SCaMC-1 OS=Mus musculus GN=Slc25a	sp|Q8BMD8|SCMC1_MOUSE	53 kDa	0	3
Hsp70-binding protein 1 OS=Mus musculus GN=Hspbp1 PE=2 SV=1	sp|Q99P31|HPBP1_MOUSE (+1)	39 kDa	0	3
A-kinase anchor protein 8 OS=Mus musculus GN=Akap8 PE=1 SV=1	sp|Q9DBR0|AKAP8_MOUSE	76 kDa	2	0
NADH dehydrogenase [ubiquinone] 1 beta subcomplex subunit 4 OS=Mus musculus (	sp|Q9CQC7|NDUB4_MOUSE	15 kDa	0	3
Cysteine protease ATG4B OS=Mus musculus GN=Atg4b PE=1 SV=2	sp|Q8BGE6|ATG4B_MOUSE (+1)	44 kDa	2	0
Protein-glutamine gamma-glutamyltransferase K OS=Homo sapiens GN=TGM1 PE=1 S	sp|P22735|TGM1_HUMAN	90 kDa	0	3
Coenzyme Q-binding protein COQ10 homolog A, mitochondrial OS=Homo sapiens GN	sp|Q96MF6|CQ10A_HUMAN	28 kDa	2	0
CDGSH iron-sulfur domain-containing protein 1 OS=Mus musculus GN=Cisd1 PE=1 SV	sp|Q91WS0|CISD1_MOUSE	12 kDa	0	3
Ragulator complex protein LAMTOR2 OS=Mus musculus GN=Lamtor2 PE=1 SV=1	sp|Q9JHS3|LTOR2_MOUSE	13 kDa	0	3
Lysosome membrane protein 2 OS=Mus musculus GN=Scarb2 PE=1 SV=3	sp|O35114|SCRB2_MOUSE	54 kDa	0	3
Plakophilin-1 OS=Homo sapiens GN=PKP1 PE=1 SV=2	sp|Q13835|PKP1_HUMAN	83 kDa	0	3
Tubulin--tyrosine ligase-like protein 12 OS=Mus musculus GN=Ttll12 PE=1 SV=1	sp|Q3UDE2|TTL12_MOUSE	74 kDa	0	3
Ubiquitin fusion degradation protein 1 homolog OS=Mus musculus GN=Ufd1l PE=1 S\	sp|P70362|UFD1_MOUSE (+1)	34 kDa	2	0
Proteasome-associated protein ECM29 homolog OS=Homo sapiens GN=ECM29 PE=1	sp|Q5VYK3|ECM29_HUMAN (+1)	204 kDa	2	0
Eukaryotic translation initiation factor 3 subunit C OS=Mus musculus GN=Eif3c PE=1 S	sp|Q8R1B4|EIF3C_MOUSE (+1)	106 kDa	2	0
Leucyl-cystinyl aminopeptidase OS=Mus musculus GN=Lnpep PE=1 SV=1	sp|Q8C129|LCAP_MOUSE	117 kDa	2	0
Sorting and assembly machinery component 50 homolog OS=Mus musculus GN=Sam	sp|Q8BGH2|SAM50_MOUSE	52 kDa	0	3
Nuclear factor NF-kappa-B p105 subunit OS=Homo sapiens GN=NFKB1 PE=1 SV=2	sp|P19838|NFKB1_HUMAN (+1)	105 kDa	0	3
Acyl-coenzyme A thioesterase 2, mitochondrial OS=Mus musculus GN=Acot2 PE=1 SV	sp|Q9QYR9|ACOT2_MOUSE	50 kDa	0	3
Eukaryotic translation initiation factor 3 subunit G OS=Homo sapiens GN=EIF3G PE=1	sp|O75821|EIF3G_HUMAN (+1)	36 kDa	0	3
39S ribosomal protein L41, mitochondrial OS=Mus musculus GN=Mrpl41 PE=2 SV=1	sp|Q9CQN7|RM41_MOUSE	15 kDa	2	0
Sideroflexin-3 OS=Mus musculus GN=Sfxn3 PE=1 SV=1	sp|Q91V61|SFXN3_MOUSE	35 kDa	0	3
NSFL1 cofactor p47 OS=Mus musculus GN=Nsfl1c PE=1 SV=1	sp|Q9CZ44|NSF1C_MOUSE (+1)	41 kDa	0	3
Activated RNA polymerase II transcriptional coactivator p15 OS=Mus musculus GN=S	sp|P11031|TCP4_MOUSE	14 kDa	0	3
Nuclear cap-binding protein subunit 2 OS=Homo sapiens GN=NCBP2 PE=1 SV=1	sp|P52298|NCBP2_HUMAN (+1)	18 kDa	2	0
Signal peptidase complex catalytic subunit SEC11A OS=Homo sapiens GN=SEC11A PE	sp|P67812|SC11A_HUMAN (+1)	21 kDa	0	3
Ubiquitin-like-conjugating enzyme ATG3 OS=Mus musculus GN=Atg3 PE=1 SV=1	sp|Q9CPX6|ATG3_MOUSE	36 kDa	2	0
E3 ubiquitin-protein ligase KCMF1 OS=Mus musculus GN=Kcmf1 PE=2 SV=1	sp|Q80UY2|KCMF1_MOUSE (+1)	42 kDa	2	0
Tax1-binding protein 3 OS=Homo sapiens GN=TAX1BP3 PE=1 SV=2	sp|O14907|TX1B3_HUMAN (+1)	14 kDa	0	3
Small ubiquitin-related modifier 3 OS=Homo sapiens GN=SUMO3 PE=1 SV=2	sp|P55854|SUMO3_HUMAN (+3)	12 kDa	0	3
NADH dehydrogenase [ubiquinone] 1 alpha subcomplex subunit 2 OS=Mus musculus	sp|Q9CQ75|NDUA2_MOUSE	11 kDa	0	3
Desmoglein-1 OS=Homo sapiens GN=DSG1 PE=1 SV=2	sp|Q02413|DSG1_HUMAN	114 kDa	0	3
Pyroglutamyl-peptidase 1 OS=Mus musculus GN=Pgpep1 PE=2 SV=1	sp|Q9ESW8|PGPI_MOUSE (+1)	23 kDa	2	0
Protein SCO2 homolog, mitochondrial OS=Mus musculus GN=Sco2 PE=2 SV=1	sp|Q8VCL2|SCO2_MOUSE	29 kDa	0	3
Basic leucine zipper and W2 domain-containing protein 2 OS=Mus musculus GN=Bzw	sp|Q91VK1|BZW2_MOUSE (+1)	48 kDa	0	3
Chromodomain-helicase-DNA-binding protein 4 OS=Mus musculus GN=Chd4 PE=1 SV	sp|Q6PDQ2|CHD4_MOUSE	218 kDa	2	0
Cysteine and glycine-rich protein 1 OS=Homo sapiens GN=CSRP1 PE=1 SV=3	sp|P21291|CSRP1_HUMAN (+1)	21 kDa	0	3
Probable ATP-dependent RNA helicase DDX46 OS=Homo sapiens GN=DDX46 PE=1 SV	sp|Q7L014|DDX46_HUMAN	117 kDa	2	0
Coiled-coil domain-containing protein 57 OS=Mus musculus GN=Ccdc57 PE=2 SV=1	sp|Q6PHN1|CCD57_MOUSE	116 kDa	0	2
Cytochrome b-c1 complex subunit 7 OS=Mus musculus GN=Uqcrb PE=1 SV=3	sp|Q9D855|QCR7_MOUSE	14 kDa	0	3
Acylamino-acid-releasing enzyme OS=Mus musculus GN=Apeh PE=2 SV=2	sp|Q8R146|APEH_MOUSE	82 kDa	0	3
Large proline-rich protein BAT3 OS=Homo sapiens GN=BAT3 PE=1 SV=2	sp|P46379|BAT3_HUMAN (+1)	119 kDa	2	0
Peroxiredoxin-6 OS=Mus musculus GN=Prdx6 PE=1 SV=3	sp|O08709|PRDX6_MOUSE	25 kDa	0	4
Mitochondrial import inner membrane translocase subunit TIM44 OS=Homo sapiens	sp|O43615|TIM44_HUMAN	51 kDa	0	2
AP-1 complex subunit gamma-1 OS=Homo sapiens GN=AP1G1 PE=1 SV=5	sp|O43747|AP1G1_HUMAN (+1)	91 kDa	0	2
Proteasome subunit beta type-5 OS=Mus musculus GN=Psmb5 PE=1 SV=3	sp|O55234|PSB5_MOUSE (+1)	29 kDa	0	2
E3 ubiquitin-protein ligase listerin OS=Mus musculus GN=Ltn1 PE=1 SV=3	sp|Q6A009|LTN1_MOUSE	199 kDa	2	0
Annexin A6 OS=Homo sapiens GN=ANXA6 PE=1 SV=3	sp|P08133|ANXA6_HUMAN	76 kDa	0	36
39S ribosomal protein L22, mitochondrial OS=Mus musculus GN=Mrpl22 PE=2 SV=1	sp|Q8BU88|RM22_MOUSE	24 kDa	2	0
Proteasome subunit alpha type-4 OS=Homo sapiens GN=PSMA4 PE=1 SV=1	sp|P25789|PSA4_HUMAN (+1)	29 kDa	0	2
26S proteasome non-ATPase regulatory subunit 7 OS=Mus musculus GN=Psmd7 PE=1	sp|P26516|PSD7_MOUSE (+1)	37 kDa	0	2
Proteasome subunit beta type-6 OS=Homo sapiens GN=PSMB6 PE=1 SV=4	sp|P28072|PSB6_HUMAN (+1)	25 kDa	0	2
Extended synaptotagmin-2 OS=Mus musculus GN=Esyt2 PE=1 SV=1	sp|Q3TZZ7|ESYT2_MOUSE	94 kDa	0	2
Methylmalonic aciduria type A homolog, mitochondrial OS=Mus musculus GN=Mmaa	sp|Q8C7H1|MMAA_MOUSE	46 kDa	2	0
Cancer-related nucleoside-triphosphatase homolog OS=Mus musculus GN=Ntpcr PE=	sp|Q9CQA9|NTPCR_MOUSE	21 kDa	2	0
Protein-L-isoaspartate O-methyltransferase domain-containing protein 2 OS=Mus mu	sp|Q8BHD8|PCMD2_MOUSE	41 kDa	0	3
Protein phosphatase 1B OS=Homo sapiens GN=PPM1B PE=1 SV=1	sp|O75688|PPM1B_HUMAN	53 kDa	2	0
Nucleoprotein TPR OS=Homo sapiens GN=TPR PE=1 SV=3	sp|P12270|TPR_HUMAN	267 kDa	2	0
YTH domain-containing protein 1 OS=Homo sapiens GN=YTHDC1 PE=1 SV=3	sp|Q96MU7|YTDC1_HUMAN	85 kDa	2	0
Signal recognition particle 14 kDa protein OS=Mus musculus GN=Srp14 PE=1 SV=1	sp|P16254|SRP14_MOUSE (+1)	13 kDa	2	0
Pre-rRNA-processing protein TSR1 homolog OS=Mus musculus GN=Tsr1 PE=2 SV=1	sp|Q5SWD9|TSR1_MOUSE	92 kDa	2	0

Inositol-3-phosphate synthase 1 OS=Mus musculus GN=Isyna1 PE=2 SV=1	sp|Q9JHU9|INO1_MOUSE (+1)	61 kDa	0	2
Acyl-coenzyme A thioesterase 13 OS=Homo sapiens GN=ACOT13 PE=1 SV=1	sp|Q9NPJ3|ACO13_HUMAN	15 kDa	0	2
RING-box protein 2 OS=Homo sapiens GN=RNF7 PE=1 SV=1	sp|Q9UBF6|RBX2_HUMAN (+1)	13 kDa	0	2
Mitochondrial Rho GTPase 2 OS=Mus musculus GN=Rhot2 PE=2 SV=1	sp|Q8JZN7|MIRO2_MOUSE	69 kDa	0	2
28S ribosomal protein S7, mitochondrial OS=Mus musculus GN=Mrps7 PE=2 SV=1	sp|Q80X85|RT07_MOUSE	28 kDa	2	0
Lipoyltransferase 1, mitochondrial OS=Mus musculus GN=Lipt1 PE=2 SV=1	sp|Q8VCM4|LIPT_MOUSE	42 kDa	2	0
39S ribosomal protein L18, mitochondrial OS=Mus musculus GN=Mrpl18 PE=2 SV=1	sp|Q9CQL5|RM18_MOUSE	21 kDa	2	0
Nuclear migration protein nudC OS=Mus musculus GN=Nudc PE=1 SV=1	sp|O35685|NUDC_MOUSE	38 kDa	0	2
39S ribosomal protein L12, mitochondrial OS=Mus musculus GN=Mrpl12 PE=1 SV=2	sp|Q9DB15|RM12_MOUSE	22 kDa	2	0
Dual specificity mitogen-activated protein kinase kinase 1 OS=Mus musculus GN=Ma	sp|P31938|MP2K1_MOUSE (+1)	43 kDa	0	2
Ras-related protein Rab-11B OS=Mus musculus GN=Rab11b PE=1 SV=3	sp|P46638|RB11B_MOUSE (+3)	24 kDa	0	2
Uridine-cytidine kinase-like 1 OS=Mus musculus GN=Uckl1 PE=1 SV=1	sp|Q91YL3|UCKL1_MOUSE (+1)	61 kDa	0	2
5'-3' exoribonuclease 2 OS=Mus musculus GN=Xrn2 PE=1 SV=1	sp|Q9DBR1|XRN2_MOUSE	109 kDa	2	0
39S ribosomal protein L46, mitochondrial OS=Mus musculus GN=Mrpl46 PE=2 SV=1	sp|Q9EQI8|RM46_MOUSE	32 kDa	2	0
Peptidyl-prolyl cis-trans isomerase D OS=Mus musculus GN=Ppid PE=1 SV=3	sp|Q9CR16|PPID_MOUSE	41 kDa	0	3
WD repeat-containing protein mio OS=Mus musculus GN=Mios PE=2 SV=2	sp|Q8VE19|MIO_MOUSE (+1)	98 kDa	0	2
Bcl-2-like protein 13 OS=Mus musculus GN=Bcl2l13 PE=1 SV=2	sp|P59017|B2L13_MOUSE	47 kDa	2	0
Probable cytosolic iron-sulfur protein assembly protein CIAO1 OS=Homo sapiens GN=	sp|O76071|CIAO1_HUMAN (+1)	38 kDa	2	0
Inosine triphosphate pyrophosphatase OS=Homo sapiens GN=ITPA PE=1 SV=2	sp|Q9BY32|ITPA_HUMAN (+1)	21 kDa	2	0
Serpin B12 OS=Homo sapiens GN=SERPINB12 PE=1 SV=1	sp|Q96P63|SPB12_HUMAN	46 kDa	0	2
Eukaryotic translation initiation factor 5 OS=Homo sapiens GN=EIF5 PE=1 SV=2	sp|P55010|IF5_HUMAN (+1)	49 kDa	0	2
60S acidic ribosomal protein P2 OS=Mus musculus GN=Rplp2 PE=1 SV=3	sp|P99027|RLA2_MOUSE	12 kDa	0	2
Tubulin-specific chaperone A OS=Mus musculus GN=Tbca PE=2 SV=3	sp|P48428|TBCA_MOUSE	13 kDa	0	2
28S ribosomal protein S24, mitochondrial OS=Mus musculus GN=Mrps24 PE=2 SV=1	sp|Q9CQV5|RT24_MOUSE	19 kDa	2	0
Ferritin heavy chain OS=Mus musculus GN=Fth1 PE=1 SV=2	sp|P09528|FRIH_MOUSE	21 kDa	0	2
Proteasome subunit alpha type-3 OS=Mus musculus GN=Psma3 PE=1 SV=3	sp|O70435|PSA3_MOUSE (+1)	28 kDa	0	2
Peptidyl-prolyl cis-trans isomerase FKBP4 OS=Mus musculus GN=Fkbp4 PE=1 SV=5	sp|P30416|FKBP4_MOUSE	52 kDa	0	2
Ubiquitin carboxyl-terminal hydrolase 14 OS=Homo sapiens GN=USP14 PE=1 SV=3	sp|P54578|UBP14_HUMAN (+1)	56 kDa	0	2
DNA replication licensing factor MCM6 OS=Mus musculus GN=Mcm6 PE=1 SV=1	sp|P97311|MCM6_MOUSE (+1)	93 kDa	0	2
ATP synthase subunit e, mitochondrial OS=Mus musculus GN=Atp5i PE=1 SV=2	sp|Q06185|ATP5I_MOUSE	8 kDa	0	2
Xaa-Pro aminopeptidase 1 OS=Mus musculus GN=Xpnpep1 PE=2 SV=1	sp|Q6P1B1|XPP1_MOUSE (+1)	70 kDa	0	2
N-alpha-acetyltransferase 35, NatC auxiliary subunit OS=Homo sapiens GN=NAA35 PE	sp|Q5VZE5|NAA35_HUMAN (+1)	84 kDa	2	0
ATP synthase subunit g, mitochondrial OS=Mus musculus GN=Atp5l PE=1 SV=1	sp|Q9CPQ8|ATP5L_MOUSE	11 kDa	0	2
Uridine-cytidine kinase 1 OS=Mus musculus GN=Uck1 PE=2 SV=2	sp|P52623|UCK1_MOUSE (+1)	31 kDa	2	0
Superoxide dismutase [Mn], mitochondrial OS=Homo sapiens GN=SOD2 PE=1 SV=2	sp|P04179|SODM_HUMAN (+1)	25 kDa	0	2
Ras-related protein Rab-10 OS=Homo sapiens GN=RAB10 PE=1 SV=1	sp|P61026|RAB10_HUMAN (+1)	23 kDa	0	4
Coronin-2B OS=Mus musculus GN=Coro2b PE=2 SV=2	sp|Q8BH44|COR2B_MOUSE	55 kDa	0	3
Steroid hormone receptor ERR1 OS=Mus musculus GN=Esrra PE=1 SV=4	sp|O08580|ERR1_MOUSE	45 kDa	0	2
Dehydrogenase/reductase SDR family member 1 OS=Mus musculus GN=Dhrs1 PE=2 S	sp|Q99L04|DHRS1_MOUSE	34 kDa	2	0
28S ribosomal protein S33, mitochondrial OS=Mus musculus GN=Mrps33 PE=2 SV=1	sp|Q9D2R8|RT33_MOUSE	12 kDa	2	0
Transcription elongation factor SPT5 OS=Homo sapiens GN=SUPT5H PE=1 SV=1	sp|O00267|SPT5H_HUMAN (+1)	121 kDa	2	0
Cartilage-associated protein OS=Homo sapiens GN=CRTAP PE=1 SV=1	sp|O75718|CRTAP_HUMAN (+1)	47 kDa	0	2
Glucose-6-phosphate 1-dehydrogenase OS=Homo sapiens GN=G6PD PE=1 SV=4	sp|P11413|G6PD_HUMAN (+1)	59 kDa	0	2
Glucosylceramidase OS=Mus musculus GN=Gba PE=1 SV=1	sp|P17439|GLCM_MOUSE	58 kDa	0	2
Histidyl-tRNA synthetase, cytoplasmic OS=Mus musculus GN=Hars PE=2 SV=2	sp|Q61035|SYHC_MOUSE	57 kDa	0	2
CDGSH iron-sulfur domain-containing protein 2 OS=Homo sapiens GN=CISD2 PE=1 SV	sp|Q8N5K1|CISD2_HUMAN (+1)	15 kDa	0	2
RING finger protein 126 OS=Mus musculus GN=Rnf126 PE=1 SV=1	sp|Q91YL2|RN126_MOUSE	34 kDa	2	0
Probable E3 ubiquitin-protein ligase HERC4 OS=Homo sapiens GN=HERC4 PE=1 SV=1	sp|Q5GLZ8|HERC4_HUMAN (+1)	119 kDa	2	0
Aminoacyl tRNA synthase complex-interacting multifunctional protein 1 OS=Mus mu:	sp|P31230|AIMP1_MOUSE	34 kDa	0	2
Iron-sulfur protein NUBPL OS=Mus musculus GN=Nubpl PE=2 SV=2	sp|Q9CWD8|NUBPL_MOUSE	34 kDa	2	0
Protein mago nashi homolog OS=Homo sapiens GN=MAGOH PE=1 SV=1	sp|P61326|MGN_HUMAN (+3)	17 kDa	0	2
Glutaredoxin-related protein 5, mitochondrial OS=Mus musculus GN=Glrx5 PE=2 SV=	sp|Q80Y14|GLRX5_MOUSE	16 kDa	0	2
28S ribosomal protein S16, mitochondrial OS=Mus musculus GN=Mrps16 PE=2 SV=1	sp|Q9CPX7|RT16_MOUSE (+1)	15 kDa	2	0
Complex I intermediate-associated protein 30, mitochondrial OS=Mus musculus GN=	sp|Q9CWX2|CIA30_MOUSE	38 kDa	2	0
NADH dehydrogenase [ubiquinone] iron-sulfur protein 7, mitochondrial OS=Mus mu:	sp|Q9DC70|NDUS7_MOUSE	25 kDa	2	0
E3 ubiquitin-protein ligase MIB1 OS=Mus musculus GN=Mib1 PE=1 SV=1	sp|Q80SY4|MIB1_MOUSE	110 kDa	2	0
Trifunctional purine biosynthetic protein adenosine-3 OS=Homo sapiens GN=GART P	sp|P22102|PUR2_HUMAN	108 kDa	0	2
Thioredoxin domain-containing protein 5 OS=Mus musculus GN=Txndc5 PE=1 SV=2	sp|Q91W90|TXND5_MOUSE	46 kDa	0	2
PDZ and LIM domain protein 3 OS=Mus musculus GN=Pdlim3 PE=1 SV=1	sp|O70209|PDLI3_MOUSE	34 kDa	0	2
Sodium/potassium-transporting ATPase subunit beta-3 OS=Mus musculus GN=Atp1b	sp|P97370|AT1B3_MOUSE	32 kDa	0	2
Glucosamine--fructose-6-phosphate aminotransferase [isomerizing] 1 OS=Mus muscu	sp|P47856|GFPT1_MOUSE (+1)	79 kDa	2	0
UV excision repair protein RAD23 homolog B OS=Homo sapiens GN=RAD23B PE=1 SV	sp|P54727|RD23B_HUMAN (+1)	43 kDa	2	0
Protein FAM36A OS=Mus musculus GN=Fam36a PE=2 SV=1	sp|Q9D7J4|FA36A_MOUSE	13 kDa	0	2
Niban-like protein 1 OS=Homo sapiens GN=FAM129B PE=1 SV=2	sp|Q96TA1|NIBL1_HUMAN	83 kDa	0	2
DIS3-like exonuclease 2 OS=Mus musculus GN=Dis3l2 PE=1 SV=1	sp|Q8CI75|DI3L2_MOUSE	98 kDa	2	0
Microtubule-associated proteins 1A/1B light chain 3 beta 2 OS=Homo sapiens GN=M	sp|A6NCE7|MP3B2_HUMAN (+4)	15 kDa	0	2
Protein QIL1 OS=Mus musculus GN=Qil1 PE=2 SV=1	sp|Q8R404|QIL1_MOUSE	13 kDa	0	2
Src substrate cortactin OS=Homo sapiens GN=CTTN PE=1 SV=2	sp|Q14247|SRC8_HUMAN	62 kDa	0	27
Dihydrofolate reductase OS=Homo sapiens GN=DHFR PE=1 SV=2	sp|P00374|DYR_HUMAN	21 kDa	2	0
Lysozyme C-1 OS=Mus musculus GN=Lyz1 PE=1 SV=1	sp|P17897|LYZ1_MOUSE (+1)	17 kDa	0	2
Amine oxidase [flavin-containing] A OS=Mus musculus GN=Maoa PE=1 SV=3	sp|Q64133|AOFA_MOUSE	60 kDa	0	2
Nuclear receptor coactivator 5 OS=Mus musculus GN=Ncoa5 PE=1 SV=1	sp|Q91W39|NCOA5_MOUSE	65 kDa	2	0
Tyrosine-protein phosphatase non-receptor type 9 OS=Mus musculus GN=Ptpn9 PE=:	sp|O35239|PTN9_MOUSE (+1)	68 kDa	2	0
NADH-ubiquinone oxidoreductase chain 1 OS=Mus musculus GN=Mtnd1 PE=1 SV=3	sp|P03888|NU1M_MOUSE	36 kDa	0	2
Transformer-2 protein homolog beta OS=Homo sapiens GN=TRA2B PE=1 SV=1	sp|P62995|TRA2B_HUMAN (+1)	34 kDa	2	0
Nucleoredoxin OS=Mus musculus GN=Nxn PE=1 SV=1	sp|P97346|NXN_MOUSE	48 kDa	2	0
Eukaryotic peptide chain release factor GTP-binding subunit ERF3A OS=Homo sapien:	sp|P15170|ERF3A_HUMAN (+1)	56 kDa	0	2

Protein Mpv17 OS=Mus musculus GN=Mpv17 PE=2 SV=1	sp|P19258|MPV17_MOUSE	20 kDa	0	2
Ribonuclease inhibitor OS=Homo sapiens GN=RNH1 PE=1 SV=2	sp|P13489|RINI_HUMAN	50 kDa	2	0
Bifunctional methylenetetrahydrofolate dehydrogenase/cyclohydrolase, mitochondri	sp|P18155|MTDC_MOUSE	38 kDa	2	0
Activating signal cointegrator 1 complex subunit 1 OS=Mus musculus GN=Ascc1 PE=2	sp|Q9D8Z1|ASCC1_MOUSE	41 kDa	2	0
Epoxide hydrolase 1 OS=Mus musculus GN=Ephx1 PE=1 SV=2	sp|Q9D379|HYEP_MOUSE	53 kDa	0	2
Acidic leucine-rich nuclear phosphoprotein 32 family member A OS=Mus musculus G	sp|O35381|AN32A_MOUSE (+2)	29 kDa	0	2
5'-AMP-activated protein kinase catalytic subunit alpha-1 OS=Homo sapiens GN=PRK.	sp|Q13131|AAPK1_HUMAN (+1)	64 kDa	2	0
Fatty acid desaturase 3 OS=Mus musculus GN=Fads3 PE=2 SV=2	sp|Q9JJE7|FADS3_MOUSE	51 kDa	2	0
DNA-directed RNA polymerases I, II, and III subunit RPABC4 OS=Homo sapiens GN=P(	sp|P53803|RPAB4_HUMAN (+1)	7 kDa	0	2
DCC-interacting protein 13-alpha OS=Mus musculus GN=Appl1 PE=1 SV=1	sp|Q8K3H0|DP13A_MOUSE (+1)	79 kDa	0	2
Translation initiation factor eIF-2B subunit gamma OS=Homo sapiens GN=EIF2B3 PE=	sp|Q9NR50|EI2BG_HUMAN	50 kDa	2	0
Mannose-6-phosphate isomerase OS=Mus musculus GN=Mpi PE=2 SV=1	sp|Q924M7|MPI_MOUSE	47 kDa	0	2
