# Supplementary material for: SH3BP2 deficiency in skeletal muscle leads to neuromuscular junction abnormalities and decreased muscle strength
Source: Front Mol Neurosci. 2026 Oct 5;19:1985823. doi: 10.3389/fnmol.2026.1985823 (PMC13677847; doi:10.3389/fnmol.2026.1985823)
Supplement: Supplementary file 2 [file Data_Sheet_2.docx]

| Antibodies | Source | RRID | Identified | Dilution for IF | Dilution for WB | Link |
| --- | --- | --- | --- | --- | --- | --- |
| anti-SH3BP2 | Abnova | RRID:AB_464258 | H00006452-M01 | 1:200 | 1:3000 | <https://www.abnova.com/en-global/product/detail/H00006452-M01> |
| anti-Synaptophysin 1 | Sysy | N/A | 101004 | 1:50 | N/A | <https://www.sysy.com/product/101004> |
| anti-FLAG | Sigma | RRID:AB_259529 | F3165 | N/A | 1:10000 | <https://www.sigmaaldrich.com/PL/pl/product/sigma/f3165?gclid=EAIaIQobChMIwf_i4sbXggMVe4RoCR00AwkXEAAYASAAEgIrvfD_BwE> |
| anti-GFP | Invitrogen | AB_221568 | A11120 | N/A | 1:3000 | <https://www.thermofisher.com/antibody/product/GFP-Antibody-clone-3E6-Monoclonal/A-11120> |
| anti-GFP | GeneTex | AB_1950371 | GTX113617 | N/A | 1:3000 | <https://www.genetex.com/Product/Detail/GFP-antibody/GTX113617> |
| anti-α-Tubulin | Abcam | RRID:AB_2210057 | ab18251 | N/A | 1:20000 | <https://www.abcam.com/en-pl/products/primary-antibodies/alpha-tubulin-antibody-microtubule-marker-ab18251> |
| anti-c-MYC | Thermofisher scientific | RRID:AB_558470 | MA1-980 clone 9E10 | N/A | 1:3000 | <https://www.thermofisher.com/antibody/product/c-Myc-Antibody-clone-9E10-Monoclonal/MA1-980> |
| anti-GST | Sigma | RRID:AB_259965 | G7781 | N/A | 1:2000 | <https://www.sigmaaldrich.com/PL/pl/product/sigma/g7781> |
| anti-RFP | Rockland | AB_2209751 | 600-401-379 | N/A | 1:1000 | <https://www.rockland.com/categories/primary-antibodies/rfp-antibody-pre-adsorbed-600-401-379/> |
| anti-GAPDH | Sigma | RRID:AB_2107445 | MAB374 | N/A | 1:10000 | <https://www.sigmaaldrich.com/PL/pl/product/mm/mab374> |
| anti-AChRδ | Thermofisher scientific | RRID:AB_2081037 | MA3-043 | N/A | 1:3000 | <https://www.fishersci.com/shop/products/chrna1-monoclonal-antibody-88b-invitrogen/MA3043> |
| anti-AChRα1 | Santa Cruz Biotechnology | AB_2080380 | sc-65829 | N/A | 1:1000 | <https://datasheets.scbt.com/sc-65829.pdf> |
| anti-Neurofilament | Millipore | RRID:AB_91203 | AB1991 | 1:300 | N/A | <https://www.merckmillipore.com/PL/pl/product/Anti-Neurofilament-H-200-kDa-Antibody-lysine-serine-proline-repeat,MM_NF-AB1991> |

**Supplementary Table 2**
